# Integrative omics identifies conserved and pathogen-specific responses of sepsis-causing bacteria

**DOI:** 10.1038/s41467-023-37200-w

**Published:** 2023-03-18

**Authors:** Andre Mu, William P. Klare, Sarah L. Baines, C. N. Ignatius Pang, Romain Guérillot, Nichaela Harbison-Price, Nadia Keller, Jonathan Wilksch, Nguyen Thi Khanh Nhu, Minh-Duy Phan, Bernhard Keller, Brunda Nijagal, Dedreia Tull, Saravanan Dayalan, Hwa Huat Charlie Chua, Dominik Skoneczny, Jason Koval, Abderrahman Hachani, Anup D. Shah, Nitika Neha, Snehal Jadhav, Sally R. Partridge, Amanda J. Cork, Kate Peters, Olivia Bertolla, Stephan Brouwer, Steven J. Hancock, Laura Álvarez-Fraga, David M. P. De Oliveira, Brian Forde, Ashleigh Dale, Warasinee Mujchariyakul, Calum J. Walsh, Ian Monk, Anna Fitzgerald, Mabel Lum, Carolina Correa-Ospina, Piklu Roy Chowdhury, Robert G. Parton, James De Voss, James Beckett, Francois Monty, Jessica McKinnon, Xiaomin Song, John R. Stephen, Marie Everest, Matt I. Bellgard, Matthew Tinning, Michael Leeming, Dianna Hocking, Leila Jebeli, Nancy Wang, Nouri Ben Zakour, Serhat A. Yasar, Stefano Vecchiarelli, Tonia Russell, Thiri Zaw, Tyrone Chen, Don Teng, Zena Kassir, Trevor Lithgow, Adam Jenney, Jason N. Cole, Victor Nizet, Tania C. Sorrell, Anton Y. Peleg, David L. Paterson, Scott A. Beatson, Jemma Wu, Mark P. Molloy, Anna E. Syme, Robert J. A. Goode, Adam A. Hunter, Grahame Bowland, Nicholas P. West, Marc R. Wilkins, Steven P. Djordjevic, Mark R. Davies, Torsten Seemann, Benjamin P. Howden, Dana Pascovici, Sonika Tyagi, Ralf B. Schittenhelm, David P. De Souza, Malcolm J. McConville, Jonathan R. Iredell, Stuart J. Cordwell, Richard A. Strugnell, Timothy P. Stinear, Mark A. Schembri, Mark J. Walker

**Affiliations:** 1 Department of Microbiology and Immunology, The University of Melbourne at the Peter Doherty Institute for Infection and Immunity, Melbourne, VIC Australia; 2 Wellcome Sanger Institute, Hinxton, UK; 3 Charles Perkins Centre and School of Life and Environmental Sciences, The University of Sydney, Sydney, NSW Australia; 4 Ramaciotti Centre for Genomics, School of Biotechnology and Biomolecular Sciences, University of New South Wales, Sydney, NSW Australia; 5 Bioinformatics Group, Children’s Medical Research Institute, Faculty of Medicine and Health, The University of Sydney, Sydney, NSW Australia; 6 Australian Infectious Diseases Research Centre and School of Chemistry and Molecular Biosciences, The University of Queensland, Brisbane, QLD Australia; 7 Institute for Molecular Bioscience, The University of Queensland, Brisbane, QLD Australia; 8 Metabolomics Australia, Bio21 Institute, The University of Melbourne, Melbourne, Australia; 9 Monash Proteomics and Metabolomics Facility, Monash Biomedicine Discovery Institute, Monash University, Melbourne, VIC Australia; 10 Centre for Infectious Diseases and Microbiology, Westmead Hospital/ Westmead Institute, and Sydney ID, University of Sydney, Sydney, NSW Australia; 11 Bioplatforms Australia Ltd., Sydney, NSW Australia; 12 Australian Institute for Microbiology and Infection, University of Technology Sydney, Sydney, NSW Australia; 13 Centre for Microscopy and Microanalysis, The University of Queensland, Brisbane, QLD Australia; 14 Australian Genome Research Facility Ltd., Melbourne, VIC Australia; 15 Australian Proteome Analysis Facility, Macquarie University, Sydney, Australia; 16 Office of eResearch, Queensland University of Technology, Brisbane, QLD Australia; 17 Center for Comparative Genomics, Murdoch University, Perth, WA Australia; 18 Department of Infectious Diseases, The Alfred Hospital and Central Clinical School, Monash University, Melbourne, VIC Australia; 19 Centre to Impact AMR and Infection Program, Monash Biomedicine Discovery Institute and Department of Microbiology, Monash University, Melbourne, VIC Australia; 20 Department of Pediatrics, School of Medicine, University of California at San Diego, La Jolla, CA 92093 USA; 21 Skaggs School of Pharmaceutical Sciences, University of California at San Diego, La Jolla, CA 92093 USA; 22 Centre for Clinical Research, The University of Queensland, Brisbane, QLD Australia; 23 Melbourne Bioinformatics, The University of Melbourne, Melbourne, VIC Australia; 24 Department of Biochemistry and Molecular Biology, Bio21 Molecular Science and Biotechnology Institute, University of Melbourne, Melbourne, VIC Australia; 25 Present Address: Commonwealth Scientific and Industrial Research Organisation, Clayton, VIC Australia

**Keywords:** Preclinical research, Proteomic analysis, Metabolomics, Bacterial infection

## Abstract

Even in the setting of optimal resuscitation in high-income countries severe sepsis and septic shock have a mortality of 20–40%, with antibiotic resistance dramatically increasing this mortality risk. To develop a reference dataset enabling the identification of common bacterial targets for therapeutic intervention, we applied a standardized genomic, transcriptomic, proteomic and metabolomic technological framework to multiple clinical isolates of four sepsis-causing pathogens: *Escherichia coli*, *Klebsiella pneumoniae* species complex, *Staphylococcus aureus* and *Streptococcus pyogenes*. Exposure to human serum generated a sepsis molecular signature containing global increases in fatty acid and lipid biosynthesis and metabolism, consistent with cell envelope remodelling and nutrient adaptation for osmoprotection. In addition, acquisition of cholesterol was identified across the bacterial species. This detailed reference dataset has been established as an open resource to support discovery and translational research.

## Abstract (web summary)

Severe sepsis has a high mortality rate. Here, the authors provide genomic, transcriptomic, proteomic and metabolomic data across four sepsis-causing pathogens and identify a signature of global increase in fatty acid and lipid biosynthesis as well as cholesterol acquisition.

## Introduction

Previously under-recognised, sepsis has only recently become part of the Global Burden of Disease register. Estimated at ~50 million cases with ~11 million deaths in 2017^1^, the World Health Organization (WHO) attributes ~20% of human mortality to sepsis worldwide (https://www.cidrap.umn.edu/news-perspective/2020/09/who-says-sepsis-causes-20-global-deaths), with disease burden borne disproportionately by developing countries (~85%)^2^. Even in developed nations, sepsis causes between one-third and one-half of all in-hospital deaths^3,4^, killing more people than heart attacks, stroke, or cancers of the prostate, breast or colon^5^. Sepsis (infection-associated organ failure)^2^ represents the end stage of an illness continuum and is probably three times more common and at least twice as lethal as previously thought^3,4^. In sepsis survivors, severe physical, cognitive, and psychological sequelae may persist for decades^6^. Among 5,033,257 severe sepsis hospitalisations in the U.S. between 1999 and 2008, 38.5% of cases reported a causative pathogen, with Gram-negative bacteria predominating (51.5%), followed by Gram-positive bacteria (45.6%), anaerobes (1.7%), and fungi (1.2%)^7^.

*Escherichia coli* is responsible for approximately one-quarter of all episodes of blood-stream infections (bacteremia) and ~50% of all Gram-negative bacteremias^8^. The 30-day all-cause mortality rate of *E. coli* bloodstream infection is ~16% and is significantly higher when associated with resistance to extended-spectrum β-lactam antibiotics^9^. Approximately half of all *E. coli* bloodstream infections originate from urinary tract infections, with gastrointestinal foci also common^10^. The majority of *E. coli* bloodstream infections are caused by a small group of high-risk globally dominant clones, with sequence type 131 (ST131) being one of the most common^2,11,12^. Taxonomically diverse *Klebsiella pneumoniae* species complex (KpSC) is a closely related set of species that are a major cause of community-acquired and nosocomial Gram-negative bloodstream infections often characterised by high-level antimicrobial resistance (AMR). Many KpSC (*K. pneumoniae*, *K. quasipneumoniae* subsp. *similipneumoniae, K. quasipneumoniae* subsp. *quasipneumoniae, K. variicola* subsp. *tropica*, *K. variicola* subsp. *variicola*, *K. quasivariicola*, and *K. africana*) with mucoid colony phenotypes due to encapsulation by secreted polysaccharides and at least one iron acquisition system are hyperinvasive and associated with sepsis^13^. KpSC bloodstream infections occur in ~1/10,000 individuals per year, with a case fatality rate of ~20% that varies with the setting, age group and co-morbidities^8,14^.

*Staphylococcus aureus* is both a commensal and an opportunistic Gram-positive human pathogen with multiple mechanisms for evading the host immune response and infecting diverse tissue sites. With more than 10 cases/100,000 population/year and associated mortality of up to 20%^15–17^, invasive *S. aureus* infections have major societal and economic impacts with estimated costs exceeding $2.2 billion annually in the US alone^18^. *Streptococcus pyogenes* is another Gram-positive bacterial pathogen causing severe disease including bloodstream infections, necrotising fasciitis, and puerperal sepsis in pregnant or post-partum women, altogether resulting in 660,000 cases and 160,000 deaths worldwide each year^19,20^. During invasive or bloodstream infection, *S. aureus* and *S. pyogenes* expression of superantigen toxins can lead to toxic shock syndrome and worsening sepsis^15,19^.

**Fig. 1:**
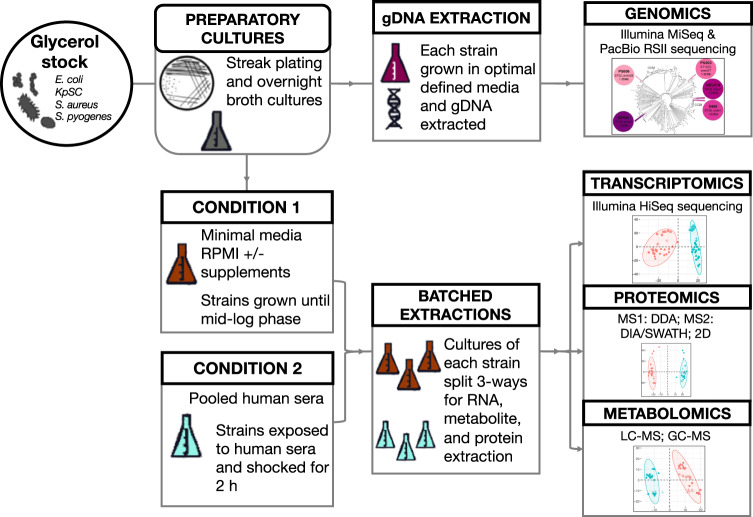
Schematic of the experimental workflow employed in this study. A sample of gDNA extracted from each strain of each species was split in order to prepare parallel sequencing libraries for both long-read (PacBio RSII) and short-read (Illumina MiSeq) sequencing. Biological replicates for transcriptomics, proteomics, and metabolomics were generated from a common glycerol stock for each strain of each species. Samples were batched in such a way that the same culture material was used to extract total RNA, proteins, and metabolites.

Once triggered, a cascade of host responses drives sepsis and early critical care interventions to support organ system function and clear the causative pathogens is essential to patient survival. Among the different types of sepsis, only bacterial infection has clear evidence for time-critical mortality benefits from targeted antibiotic therapy, and these data form the basis for the current international consensus standard of care^21^. However, the detection of the presence and antibiotic susceptibility of a pathogen may be insufficient, since increasing evidence suggests that clinical interventions are best guided by the presence or absence of specific virulence factors^22^. While important genetic determinants not evident in standard test conditions are being discovered in prominent pathogens, high-quality baseline physiological data will be essential to facilitate the future discovery of pathogen-directed diagnostics for sepsis^23^. To model the impact of transition into the human bloodstream during sepsis, we have undertaken a systematic comparison of global responses in representative leading human sepsis pathogens—comparing their growth in a tissue-model medium (RPMI) versus human serum, using integrated genomic, transcriptomic, proteomic and metabolomic methodologies (Fig. 1). This cross-species comparative investigation of multiple clinical sepsis isolates provides a compendium of both conserved and pathogen-specific responses that may provide targets for future therapeutic intervention.

## Results and discussion

### Genome analysis

**Fig. 2:**
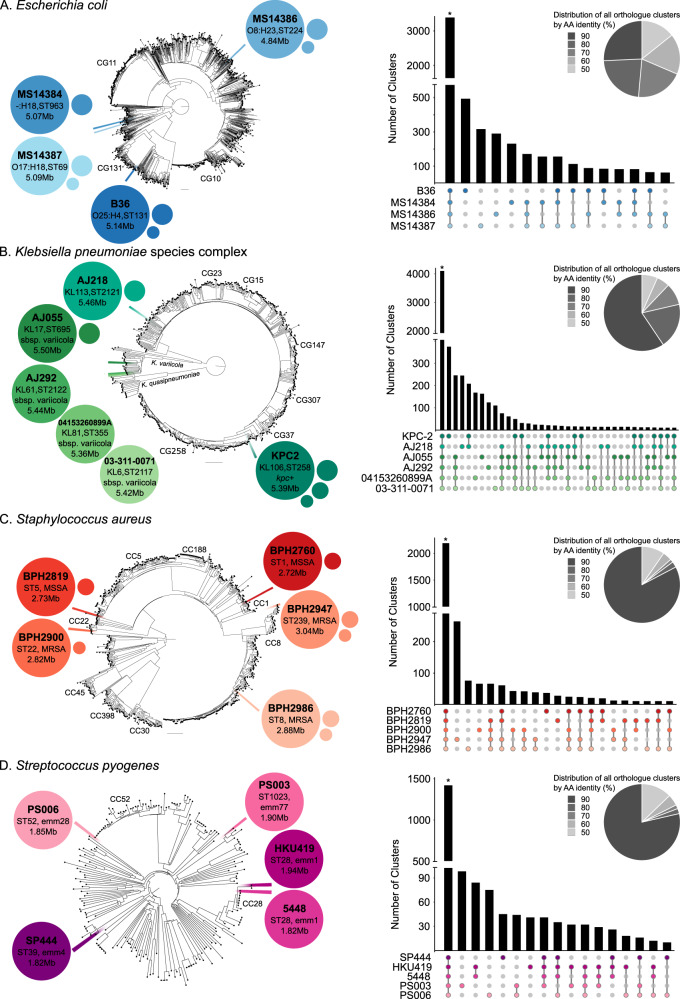
Genomic description, phylogenetic context, and shared protein orthologues of study strains. The 20 complete genomes for **A** *E. coli*; **B** KpSC; **C** *S. aureus*; **D** *S. pyogenes* are illustrated. For each, the composition of the genome is illustrated as bubbles, with the chromosome represented by the largest bubble, with key features annotated, and the smaller bubbles representing plasmids, the size of which is a semi-quantitative representation of the plasmid size. The location of each genome in the respective phylogenetic trees for each species is illustrated; the tree representing all complete genomes for the species available in GenBank as of June 2020, and constructed using Mashree v1.1.2 based on the distance calculated from 50,000 k-mers. Clonal Groups (CG) and Clonal Complexes (CC) of interest for the species are annotated. Adjacent to each tree is an UpSet plot and a pie chart, illustrating the results of hierarchical protein orthologue clustering, performed with pirate v1.0.2. Predicted protein-coding sequences were clustered at five thresholds of amino acid identity (50%, 60%, 70%, 80%, 90%). The distribution of all orthologue clusters represented in a species at these thresholds is indicated in the pie chart. The sharing of orthologue clusters amongst the respective genomes (in various combinations or in isolation) is shown in the UpSet plot, ordered based on the frequency and coloured to match the genomes as illustrated in the phylogenetic trees. Asterisks (*) indicate the orthologue clusters that are conserved within a species (i.e., the ‘core’ cluster for each species). The corresponding list of core orthologue clusters is detailed in Supplementary Data 1 (*E. coli*), 2 (*KpSC*), 3 (*S. aureus*), and 4 (*S. pyogenes*). Combinations with less than ten shared orthologue clusters are not shown.

The human sepsis pathogens investigated in this study and their clinical features of interest are listed in Table 1. Four *E. coli*, six KpSC (two *K. pneumoniae* and four *K. variicola*), five *S. aureus* and five *S. pyogenes* clinical strains were subjected to DNA sequence analysis, with each genome fully assembled and annotated using the approach outlined in Baines et al.^24^ for accurate and reproducible genome reconstruction. The genetic diversity of the strains is illustrated within a phylogenetic context, with each genome placed in the wider population context for the respective species or species complex (Fig. 2).

**Table 1:** Bacterial bacteremia and sepsis pathogens analysed in this study

Species	Strain	Disease	Source	Sample Accession	Multilocus sequence Type^a^ ST (CC)	Additional typing characteristics^b^	Genotypes of Interest^c^	Reference
*Streptococcus pyogenes*	5448	Toxic shock syndrome and necrotising fasciitis	Blood	SAMEA5096233	ST28	M1	Virulence: *hasA, hasB, hasC; speA, speG, speJ*	^78^
*Streptococcus pyogenes*	SP444	Invasive disease	Blood	SAMEA5096234	ST39	M4	Virulence: *speC; ssa*	^79^
*Streptococcus pyogenes*	HKU419	Scarlet fever and bacteremia	Blood	SAMEA5096235	ST28	M1	Acquired AMR: *erm*(B); *tet*(M) Virulence: *hasA, hasB, hasC;speA, speC, speG, speJ, ssa*	^80^
*Streptococcus pyogenes*	PS003	Puerperal sepsis	Vaginal swab	SAMEA5096236	ST1023	M77	Acquired AMR: *tet*(O) Virulence: *hasA, hasB, hasC*	^81^
*Streptococcus pyogenes*	PS006	Puerperal sepsis	Urine	SAMEA5096237	ST52	M28	Virulence: *hasA, hasB, hasC, speC,speG, speJ*	^81^
*Staphylococcus aureus*	BPH2760	Bacteraemia	Blood	SAMEA5099550	ST1 (CC1)	t286	Acquired AMR: *blaZ*	^82^
*Staphylococcus aureus*	BPH2819	Bacteraemia	Blood	SAMEA5099551	ST5 (CC5)	t002	Acquired AMR: *blaZ; fosB*	^65^
*Staphylococcus aureus*	BPH2900	Bacteraemia	Blood	SAMEA5099552	ST22 (CC22)	–	Acquired AMR: *blaZ; erm*(C); *mecA*	^65^
*Staphylococcus aureus*	BPH2947	Bacteraemia	Blood	SAMEA5099553	ST239 (CC8)	t037	Acquired AMR: *aac(6’)-Ie-aph(2”)-Ia; ant(6)-Ia*; *ant(9)-Ia; aph(3’)-IIIa*; *blaZ; dfrG*; *erm*(A); *fosB; mecA; sat; tet*(K); *tet*(M)	^65^
*Staphylococcus aureus*	BPH2986	Bacteraemia	Blood	SAMEA5099554	ST8 (CC8)	t008	Acquired AMR: *ant(6)-Ia; aph(3’)-IIIa*; *blaZ; fosB, mecA; mph*(C); *msr(A); sat* Virulence: *lukFS*	^65^
*Klebsiella pneumoniae*	AJ218	Urinary tract infection	Urine	SAMEA5128437	ST2121	KL113; wzi212	Acquired AMR: *aadA1*; blaSHV-44; *tet*(D); *sul1*	^83^
*Klebsiella pneumoniae*	KPC2	Urosepsis	Blood	SAMEA5128438	ST258	KL106; wzi29	Acquired AMR: *aac(6’)-Ib; aadA2*; *aph(3’)-Ia; bla*_KPC-2_; *bla*_SHV-12_; *catA1*; *dfrA12*; *mph*(A)*; sul1* Virulence: ybt13; ICEKp2	^84^
*Klebsiella variicola*	AJ055	Community-acquired urinary tract infection	Urine	SAMEA5128435	ST695	KL17; wzi252	–	^83^
*Klebsiella variicola*	AJ292	Sepsis	Blood	SAMEA5128436	ST2122	KL61; wzi320	–	^83^
*Klebsiella variicola*	03-311-0071	Bacteremia	Blood	SAMEA5128439	ST2117	KL6; wzi313	–	^83^
*Klebsiella variicola*	04153260899A	Urosepsis and bacteraemia	Blood	SAMEA5128440	ST355	K81; wzi81	–	This study
*Escherichia coli*	B36	Pyelonephritis/urosepsis	Blood	SAMEA5128441	ST131	O25b:H4	Acquired AMR: *aac(6’)-Ib-cr5; aadA5; bla*_CTX-M-15_*; bla*_OXA-1_; *dfrA17; mph*(A)*; sul1; tet*(A) Virulence: *afaD, chuA, fdeC, fepA, fimH, kpsD, kpsM, sat, ybtS*	^85^
*Escherichia coli*	MS14384	Urosepsis	Urine	SAMEA5128444	ST963	OgN12:H18	Acquired AMR: *bla*_CMY-2_ Virulence: *fdeC, fepA, fimH, kpsD, ytbS*	^86^
*Escherichia coli*	MS14386	Bacteremia	Blood	SAMEA5128443	ST224	O8:H23	Acquired AMR: *aac(3)-IId; aadA1; aph(3’)-Ia; bla*_TEM-1b_*; bla*_TEM-215_*; dfrA12; dfrA14; floR; fosA4; mph(*A)*; strAB*; *sul2; sul3* Virulence: *fdeC, fepA, fimH*	This study
*Escherichia coli*	MS14387	Pyelonephritis/urosepsis	Blood	SAMEA5128445	ST69	O17:H18	Acquired AMR: *bla*_TEM-1b_*; strAB; sul2* Virulence: *fdeC, fepA, fimH, iutA, kpsD, papG, sat, ybtS*	^87^

Strain characteristics were determined in silico.

^a^Multilocus sequence types (ST) and clonal complexes (CC) were determined using mlst v2.19.0 (https://github.com/tseemann/mlst; employing the pubMLST schemes^48^).

^b^*emm*-type (M) for *S. pyogenes*, capsule type (KL) for KpSC, spa type (spa) for *S. aureus*, and serotype (O:H) for *E. coli*, were determined using emmtyper v0.1.0 (https://github.com/MDU-PHL/emmtyper), Kleborate v2.0.1^50^, and the Center for Genomic Epidemiology’s (http://www.genomicepidemiology.org/) spaTyper and SeroTypeFinder, respectively.

^c^Aquired antimicrobial resistance (AMR) genes were detected using abriTAMR (https://github.com/MDU-PHL/abritamr) and virulence genes with ABRIcate (https://github.com/tseemann/abricate) or Kleborate v2.0.1 (for KpSC), requiring a ≥ 90% length and identity match; intrinsic and/or biocide genes for each strain, including *K. variicola*, can be found in Supplementary Data 11. Accession numbers for each strain can be found in detail in Supplementary Table 7.

To characterise the gene content encompassed within and shared between the genomes, a hierarchical orthologue clustering approach was employed (Fig. 2, Supplementary Data 1–5); shared amino acid identity was analysed at thresholds of 50%, 60%, 70%, 80% and 90% for all protein-coding sequences (CDS) in the 20 chromosomes (excluding plasmids). Overall, the Gram-negative genomes displayed greater genetic diversity as indicated by the higher proportion of orthologue clusters that were grouped at lower identity thresholds (Fig. 2). In comparison, the majority of orthologue clusters identified in the Gram-positive genomes shared ≥90% amino acid identity. These findings are consistent with our current understanding of genome-based differences amongst the species investigated. For example, genome sizes are approximately twice as large for *E. coli* and KpSC (4–6 Mb) compared with *S. aureus* and *S. pyogenes* (2–3 Mb). Genome conservation is lower in *E. coli* and KpSC with only ~50% of predicted genes considered as core genome compared with ~80% in *S. aureus* and *S. pyogenes*. Furthermore, gene sharing—with respect to the number, size, and diversity of mobile genetic elements—is greater within and between *E. coli* and KpSC (and other Enterobacterales) populations compared to *S. aureus* and *S. pyogenes* populations^25–28^.

**Fig. 3:**
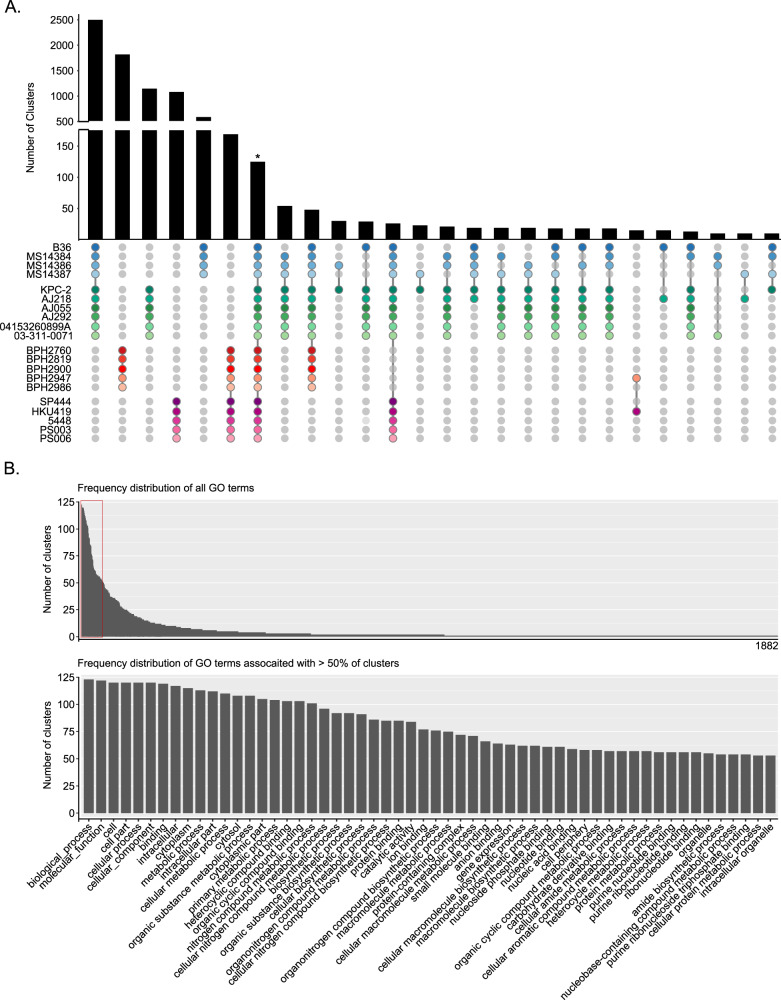
Conserved protein orthologues amongst genomes and associated functional analysis. A comparison of protein orthologue clusters across the 20 genomes investigated is illustrated. **A** The sharing of orthologue clusters across species (in various combinations) or those conserved within a species are shown in the UpSet plot, ordered based on the frequency and coloured to match the genomes as illustrated in the phylogenetic trees in Fig. 2. Combinations with less than ten shared orthologue clusters are not shown. The asterisk (*) indicates the 125 orthologue clusters that were conserved across all 20 genomes. **B** Frequency for all 1882 GO terms associated with the 125 conserved clusters (top barplot). Those associated with >50% of these clusters (outlined in red) are highlighted in the enlarged plot (bottom barplot) with their functional annotations provided. A complete list of all GO and KEGG metabolic pathway terms associated with the 125 core clusters is provided in Supplementary Data 6.

Each species/species complex (here referred to collectively as “species”, unless otherwise defined) contained a large proportion of ortholog clusters that were conserved across all strains within a species, ranging from 58.6% in *E. coli* to 70.3% in *S. aureus* (as illustrated in the UpSet plots in Fig. 2, with lists of core clusters provided in Supplementary Data 1–5). In most cases, the next highest proportion of clusters represented those genes that were unique to a given genome. An exception to this observation was KpSC, in which a high proportion of clusters were shared between the two *K. pneumoniae* genomes and similarly, between the four *K. variicola* genomes. There were 125 protein orthologue clusters that were shared amongst all 20 genomes, representing <1% of all clusters identified (Fig. 3, Supplementary Data 5). More commonly observed clusters were those conserved within and unique to a species, or shared only among the Gram-negative, or Gram-positive genomes, but not across both (Fig. 3A).

We undertook an assignment on Gene Ontology (GO) and Kyoto Encyclopaedia of Genes and Genomes (KEGG) metabolic pathway terms to explore the potential function of the 125 conserved protein orthologue clusters (Supplementary Data 6). There were 1882 non-redundant GO terms and 155 KEGG terms associated with the 125 orthologue clusters, of which only 56 GO terms were associated with more than 50% of the clusters (Fig. 3B, Supplementary Data 6). As expected, the overrepresented GO terms were associated with core cellular functions, consistent with the conservation of these orthologues across diverse genera. A strong evolutionary drive likely exists to maintain the functions of these orthologues in bacteria, and their broad conservation makes them valid targets for the development of broad-spectrum antibiotics.

### Transcriptome analysis

**Fig. 4:**
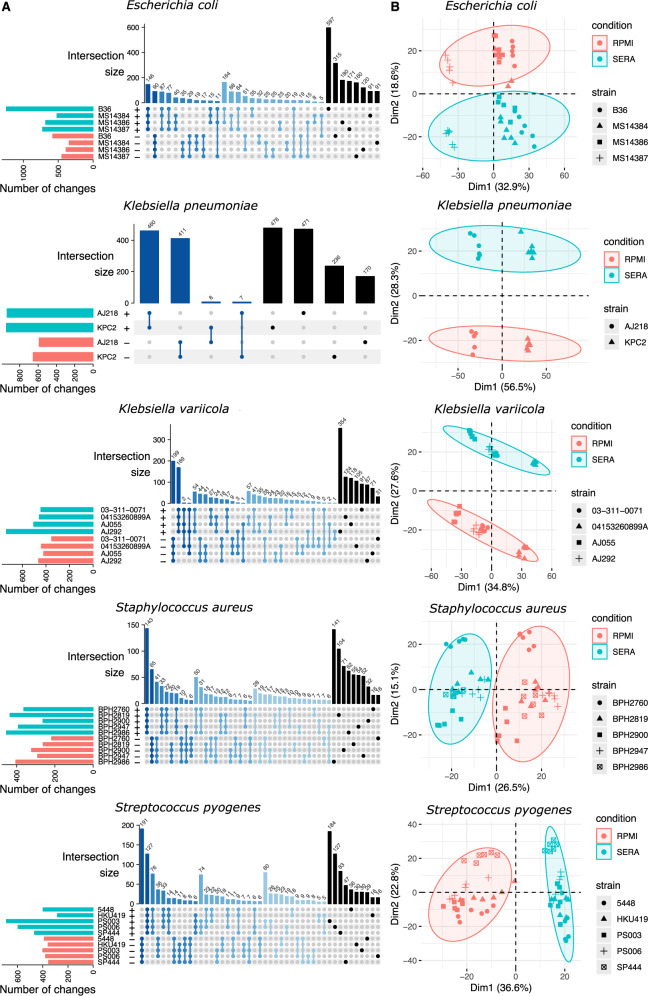
RNA-seq to assess global serum exposure impacts on the transcriptome. **A** UpSet plot representing the shared and distinctive transcriptional responses across strains of the same species. Only genes with significant differential expression after exposure to human serum are represented (FDR < 0.05; |log_2_ fold change | >1). Horizontal bars indicate the total number of significant changes *per* strain (top: up in serum, bottom: down in serum). Vertical bars represent the number of shared up/down genes across strains. Overlapping responses (intersections) are defined below the bars with connected dots. Darker blue bars represent intersections with higher numbers of strains and black bars the distinctive response for each strain. **B** Multidimensional scaling plots of the transcriptional responses of core genes across strains of the same species. Point shapes represent different strains and each point corresponds to an individual biological replicate. The colour of the points and ellipses differentiate cultured in RPMI from serum-exposed samples.

RNA sequencing (RNAseq) was used to assess the impact of exposure to serum for 2 h on the transcriptome for each of the 20 strains compared to growth in RPMI. Data were generated from four *E. coli*, six KpSC, five *S. aureus* and five *S. pyogenes* strains. Outliers were removed from the six biological replicates per strain (Supplementary Fig. 1). Differential gene expression analysis was undertaken by mapping reads to the core genomes defined by comparative genomics (Fig. 3, Supplementary Data 7). Within each species, an intersection analysis was performed to assess the extent of strain-specific or shared expression response to human serum exposure (Fig. 4A). Multidimensional scaling (MDS) was then used to visualise the clustering of gene expression responses for each biological replicate, within a strain, and in response to serum treatment (Fig. 4B). Biological replicates clustered closely together, while serum treatment led to clear separation between the first two dimensions for every species, pointing to the robustness of these data (Fig. 4B). Each species contained shared core transcription responses corresponding to only a fraction of the core genome (only 200–900 genes (dark blue bars, Fig. 4A), from core genomes consisting of 1500–4000 genes (Fig. 2). The numbers of genes responsible for these conserved transcriptional responses were far fewer than the total number of genes forming the distinctive transcriptional responses (Fig. 4A, Supplementary Data 7). This finding suggests that the strain-specific responses to serum exposure within a species are likely to be as consequential as species-wide responses. *Escherichia coli* B36 is an example of the globally dominant ST131 clone and 912 genes were uniquely differentially expressed in response to serum exposure compared to the three other *E. coli* strains (597 enriched and 315 depleted genes, FDR < 0.05; fold change >2; Fig. 4A, Supplementary Data 7). Among these genes, *fepE* (encoding a polysaccharide co-polymerase that regulates LPS O-antigen chain length) was significantly repressed, while genes involved in the uptake of carboxylate-containing organic acids were among the most upregulated. Similarly, the *K. pneumoniae* ST258 clone KPC2 (representative of a globally dominant lineage) had 730 uniquely differentially expressed genes compared with *K. pneumoniae* AJ218. These genes encoded a wide range of biosynthetic functions from enzymes involved in purine metabolism, to factors controlling protein biosynthesis. Additionally, membrane proteins, including a major facilitator superfamily transporter, were repressed in *K. pneumoniae* KPC2 in response to serum exposure (Supplementary Data 7).

In *S. aureus* BPH2986, which represents the globally-distributed ST8 “USA300” community methicillin-resistant *S. aureus* (MRSA) clone, 157 genes were uniquely differentially expressed compared with the four other *S. aureus* strains (Supplementary Data 7). Notably, the BPH2986-specific response included genes involved in nitrite dissimilation. Down-regulation of a putative nitric oxide reductase and glutamine amidotransferase and upregulation of sirohydrochlorin chelatase, involved in the biosynthesis of the cofactor siroheme used by nitrite reductases, suggest specific control of nitrogen metabolism regulatory circuits in USA300 in response to human serum compared to the other four *S. aureus* strains (Supplementary Data 7). Strain specificity was also seen in *S. pyogenes*—in the HKU419 strain associated with the Hong Kong scarlet fever outbreak of 2011, 46 genes were uniquely differentially expressed after serum exposure compared with the other four strains. These changes included the upregulation of phosphotransfer system genes involved in carbohydrate transport (Supplementary Data 7).

We next compared gene expression responses to serum exposure between the genetically distinct species, by conducting gene-set enrichment analysis using GO terms and KEGG metabolic pathways (Supplementary Fig. 2, Supplementary Data 8). There were only a modest number of shared transcriptional responses across all 20 strains compared to within the species groups (Supplementary Fig. 2). Principal component analysis was employed to assess the similarity of functional responses to human serum exposure among the 20 bacterial isolates (Fig. 4A), revealing tight clustering aligned with genetic relatedness, again pointing to the diversity of gene expression responses (Fig. 4B). A strain-based heat map using GO terms and KEGG metabolic pathways was generated; significant enrichment with the same directionality was present across at least 10 strains. While no functional categories were enriched across all strains, a few categories were enriched across a majority of genera and strains. Upregulated genes that were enriched included genes involved in propanoate, pyruvate and arginine metabolism. Notably, arginine is one of the few amino acids found at high concentrations in human serum, as it is transported between the liver and kidneys^29,30^. Enrichment of down-regulated genes was associated with ribosome function (Supplementary Data 7 and 8).

To further explore the role of upregulated arginine metabolism genes in response to growth in serum, we mutated the GAS strain 5448 *arcC* gene and complemented this mutant. The *arcC* gene encodes for carbamate kinase which is part of the arginine deiminase system, converting arginine to ornithine + 2NH_3_ + CO_2_ and generating ATP^31^. Mutagenesis of *arcC* in M1 serotype strain *S. pyogenes* 5448 (*S. pyogenes 5448*Δ*arcC*) reduced bacterial growth and the phenotype was restored to wildtype in the complemented mutant *S. pyogenes* 5448Δ*arcC::arc* (Supplementary Fig. 3). Two genes significantly upregulated in *E. coli* in response to serum were also examined further, the *carB* gene encoding the large subunit of the carbamoyl phosphate synthetase that plays a role in arginine and pyrimidine biosynthesis^32^, and *wcaF* gene encoding an acetyltransferase involved in the synthesis of the exopolysaccharide colanic acid^33^. Mutation of either *carB* or *wcaF* in *E. coli* B36 (*E. coli* B36Δ*carB* or *E. coli* B36Δ*wcaF*) reduced survival in serum (Supplementary Fig. 4A). Furthermore, independent mutation of both genes in the *E. coli* ST131 reference strain EC958 led to similar attenuated survival phenotypes (Supplementary Fig. 4B), thus validating the role of these genes in serum resistance.

### Proteome analysis

**Fig. 5:**
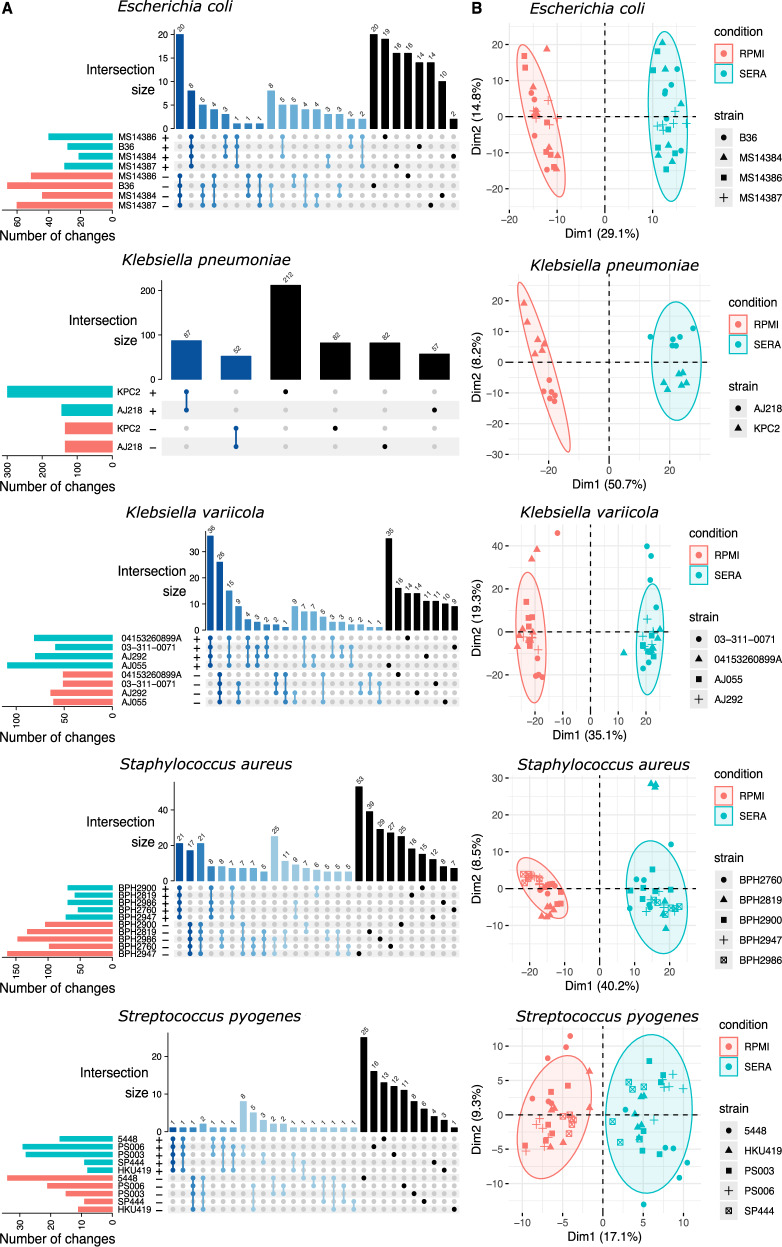
DDA mass spectrometry to assess the impact of serum exposure on the proteome within the different species. **A** UpSet plot representing the shared and distinctive proteome responses across strains of the same species. Only proteins with significant differential abundance after exposure to human serum are represented (FDR < 0.05; |log_2_ fold change | >1). **B** Multidimensional scaling plots of the core protein responses across strains of the same species demonstrating a clear separation of serum exposed samples for all species. See Fig. 4 legend for a detailed explanation.

To determine the differences in the proteomic landscape upon transition of the 20 selected strains from RPMI into human serum, we subjected all samples to high-resolution mass spectrometry using both data-dependent acquisition (DDA) and data-independent acquisition/sequential window acquisition of all theoretical mass spectra (DIA/SWATH). Differential abundance analyses were performed on both datasets, and comparative analyses of the resulting fold-changes revealed a strong correlation between these two orthogonal methodologies accentuating data quality and quantitative accuracy (Fig. 5 and Supplementary Fig. 6).

Intersection analyses revealed that, within each species, there was a shared core protein response to human serum ranging from as few as two proteins in the case of *S. pyogenes* to 139 proteins in the case of *K. pneumoniae* (Supplementary Fig. 7A). Strain-specific responses typically encompassed a similar number of proteins, confirming the notion that bacteria employ both strain-specific and species-wide responses to adapt to human serum. MDS plots were used to visualise similarities of abundance changes across biological replicates and strains within a given species (Supplementary Fig. 7B). In line with the transcriptomic data, exposure to human serum was observed to be the main driver of a clear separation in samples for each species in the first dimension, while biological replicates of the various strains clustered loosely along the second dimension (Supplementary Fig. 7B).

GO enrichment of protein classes significantly altered in abundance after serum exposure did not yield any commonly shared pathways across all strains and species (Supplementary Figs. 8 and 9). Proteins involved in response to stress were unexpectedly, and significantly, altered in certain species and strains, suggesting a differential response of individual pathogens to serum exposure. For example, the abundance of the universal stress protein family (Usp) proteins in *S. aureus* and *S. pyogenes* were not significantly altered, whereas the abundance of proteins UspF and UspG from *E. coli* and KpSC strains were only modestly altered despite their genes showing significant change at the transcript level.

GO terms relating to iron acquisition in *S. aureus* were significantly enriched across all strains, and many of the largest increases in protein abundances were associated with iron acquisition. In particular, we noticed that the Isd system, required for host haem-associated iron scavenging, was significantly upregulated in the presence of serum across all *S. aureus* strains. To demonstrate the power of these data to identify bacterial loci involved in critical host responses we assessed the impact of the loss of *isdI* on *S. aureus* growth in serum. This gene encodes an oxygenase that takes captured host haem by oxidative degradation of the porphyrin ring imported by other members of the Isd system to generate staphylobilin and then release free iron within the bacterium^34^. We used a defined *S. aureus* JE2 *isdI* transposon mutant (NE137), confirmed by complete genome sequencing and assembly to contain only the inactivating transposon in *isdI*, and conducted growth assays in the presence of human serum. We observed a growth advantage for the *isdI* mutant in RPMI alone, but in the presence of 50% heat-inactivated serum, the mutant had a significant growth defect compared to the wild type as revealed by comparison of AUC values (*p* = 0.03, Supplementary Fig. 10).

While iron acquisition proteins were more abundant upon serum exposure, many proteins involved in purine metabolism decreased across the six *S. aureus* strains. *E.coli* strains were observed to have increased levels of iron acquisition, most notably siderophore systems. Strains of KpSC had unique metabolic proteotypes relative to other species. Proteins associated with pyruvate and propanoate metabolism were increased in KpSC and *E. coli*, whereas proteins associated with nitrate and nitrite reductase function were decreased in both KpSC and *E. coli*, along with several enriched GO terms associated with organonitrogen metabolism pathways. A decrease in the abundance of proteins associated with purine synthesis was relatively widespread across all species and strains, indicative of decreased rates of cell division^35^ (Supplementary Figs. 8 & 9; Supplementary Data 9).

### Metabolome analysis

To parallel the transcriptome and proteome analyses, the metabolomes of the 20 strains were profiled after transition from RPMI into the human serum. To gain the broadest coverage of the polar metabolites, the samples were analysed on both GC–MS and LC–HILIC–MS platforms. Univariate analyses were performed on both datasets to identify significantly changed metabolites within each strain upon serum treatment (Supplementary Data 10), and then compared within species to determine whether there was a core metabolic shift in response to serum exposure.

**Fig. 6:**
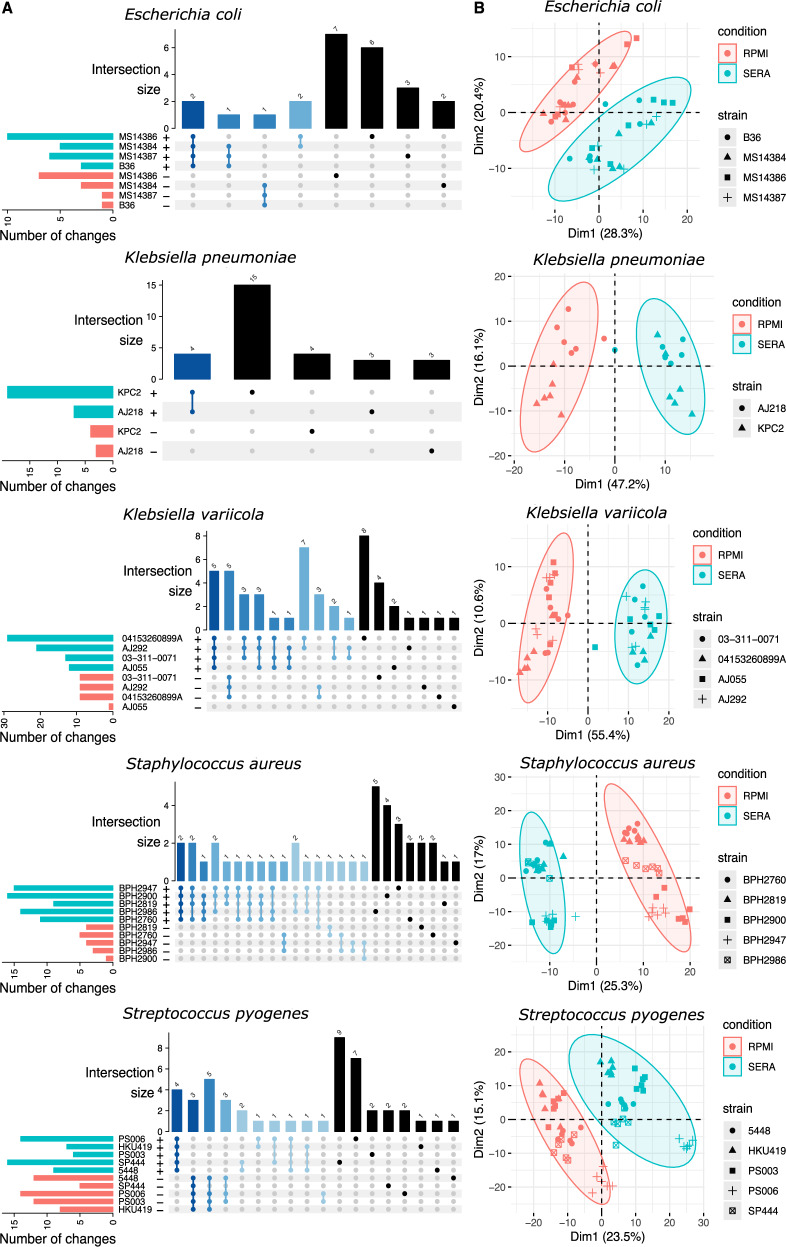
GC–MS analysis to assess the impact of serum exposure on the metabolome across the different species. **A** UpSet plot represents the shared and distinctive metabolome responses across strains of the same species. Only metabolites with significant differential abundance after exposure to human serum are represented (FDR < 0.05; |log_2_ fold change | >1). **B** Multidimensional scaling plots of the core-metabolites responses across strains of the same species demonstrating a clear separation of serum exposed samples for all species. See Fig. 4 legend for a detailed explanation.

Intersection analyses showed that there was a shared response within species. In considering both GC–MS and LC–HILIC–MS data, just 4 shared metabolic changes were identified in the *E. coli* strains, while there were 15 shared metabolic changes in the *S. pyogenes* strains (Fig. 6). Overall, there was a consistent number of metabolite changes within each strain, suggesting that while there is some level of species-wide response, there is also an individual adaptive response to serum exposure. Multi-dimensional scaling analyses were performed to observe the global metabolic effect of exposure to serum. Using data derived from both mass spectrometry platforms, we observed a consistent separation between media-grown and serum-exposed replicates and strains within a given species suggesting a marked metabolic response to serum exposure (Fig. 6B). This observation continues the trend seen in the transcriptome and proteome analyses, where exposure to serum causes the greatest variance in the first dimension, which was followed by more subtle differences observed between strains in the second dimension (Supplementary Fig. 2).

Metabolic pathway enrichment analysis of significantly altered metabolites following growth in human serum failed to reveal any commonly shared pathways across all strains and species (Supplementary Figs. 8 and 9). Furthermore, only a few pathways were enriched across all strains of a given species, such as fatty acid biosynthesis/metabolism in *S. aureus* strains and taurine/hypotaurine metabolism in KpSC strains. We next examined if there were commonalities amongst individual metabolite changes across the species. A number of metabolites involved in osmoprotectant response^36,37^ were significantly increased in several strains, these included betaine, dimethylglycine and taurine (Supplementary Fig. 11). While these changes were not consistent across all strains and species, this correlated with changes in proteins involved in osmotolerance.

The most striking observation was that the level of intracellular cholesterol increased between 200 and 1400 fold across all strains and species upon serum exposure (Supplementary Fig. 11). As these organisms are unable to synthesise cholesterol, this suggested that the increased cholesterol was incorporated from the serum either due to passive adsorption into the peripheral membrane or active uptake and accumulation in the bacterial cells.

### Multi-omics response to serum assessed by functional and metabolic pathway enrichment

**Fig. 7:**
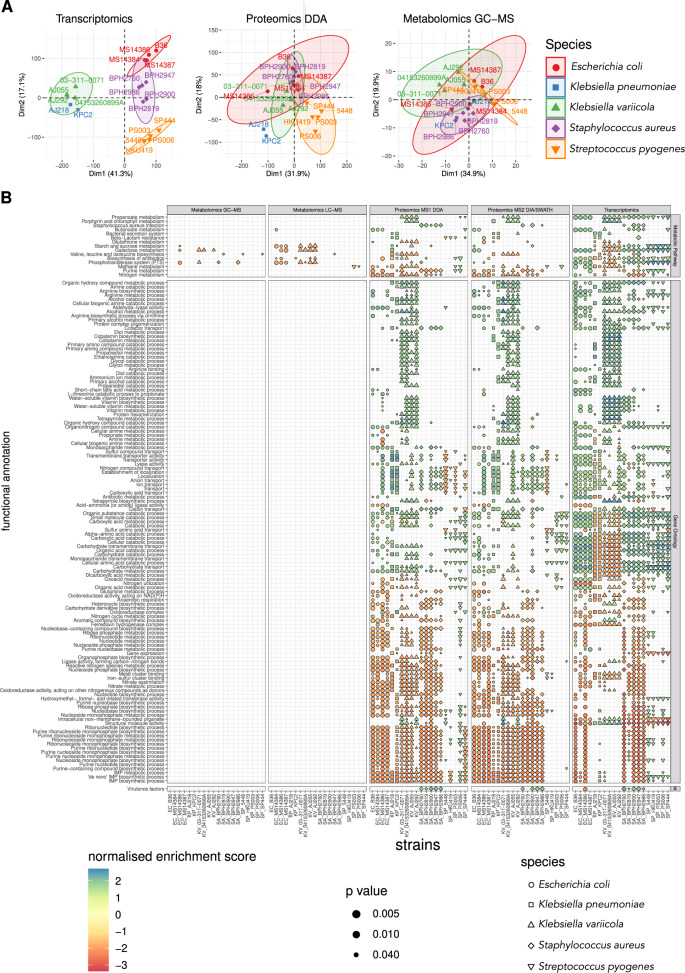
Functional and metabolic pathway enrichment analysis to assess the multi-omics response to serum. **A** Principal component analysis representing the proximity of the functional responses to human serum exposure. The data compile the response of the 20 bacterial strains by transcriptomics, proteomics and metabolomics. **B** Dot heatmap representing the shared enrichment of GO terms and KEGG pathways across all strains and the different omics. Shapes and colours represent normalised enrichment scores as calculated using Gene Set Enrichment Analysis and adjusting for multiple hypothesis testing, and indicate up (blue) and down (red) regulated functions or pathways in serum. Only enriched GO terms and KEGG metabolic pathways significantly enriched in 50% of all strains and across at least two omics datasets are represented.

Analysis of data across the multiple omic platforms showed both conserved and divergent molecular omics signatures across bacterial species in serum (Fig. 7A). Enriched GO biological pathway terms across strains from the majority of species focused largely on positive changes to fatty acid and lipid biosynthesis and metabolism, which correlated with the metabolomics changes described above. Other GO terms were related to ion transport (largely iron and haem acquisition) and amino acid metabolism. Enriched arginine biosynthesis and metabolism pathways correlated with elevated polyamines associated with osmoprotection; catabolism of other amino acids and fatty acids generates additional osmoprotectants including betaine, dimethylglycine and taurine, consistent with the metabolomics data. Conserved omics signatures from serum-derived cells showed enrichment of terms associated with adaptation to osmotic stress, oxidative stress and iron limitation. To validate observations of changes in osmotic stress response, oxidative stress response and iron starvation response, we tested whether cells pre-incubated in serum survived under stress differently from those pre-incubated in RPMI. Three of four species (*K. pneumoniae*, *E. coli* and *S. pyogenes*) showed significantly increased survival post-osmotic stress conferred by 150 mM NaCl following prior exposure to serum compared to RPMI medium. Two species also showed significantly increased survival under each of oxidative stress (*K. pneumoniae* and *E. coli*) and prolonged iron limitation (*E. coli* and *S. aureus*) (Supplementary Fig. 12). These data show that serum exposure confers increased stress resistance in these bacteria; this is most evident for osmotic stress and aligns well with the proteomics and metabolomics observations showing increases in abundance of enzymes responsible for a range of similarly elevated small molecules that are involved in osmoadaptation and tolerance.

Many species exhibited proteomic and transcriptomic evidence of heightened carbohydrate, organic acid and dicarboxylate metabolism, although the converse was observed for KpSC at the transcript level. Almost all species demonstrated a reduced commitment to nucleotide (predominantly purine) biosynthesis and metabolism (Fig. 7B). The global multi-omics profile indicated almost all strains responded to the shift into serum with metabolic adaptations associated with redirecting carbohydrate, amino acid, organic acid, fatty acid and lipid biosynthesis and metabolism towards membrane and cell wall remodelling in serum. Increased lipid biosynthesis requires sufficient pyrimidine pools, to condense CDP-lipid intermediates, and carbohydrate synthesis also requires pyrimidine pools to condense UDP-sugar intermediates. While purine biosynthetic pathways were suppressed in many strains, pyrimidine pathways were not. To survive in adverse environments, many bacteria are known to modify the lipid composition of their membranes^38^. Fatty acid incorporation into membrane phospholipids alters membrane fluidity^39^. We observed increased cell-associated fatty acid abundances across almost all species after exposure to serum (Fig. 6). Bacteria may also use fatty acids as a carbon source. We observed significant transcript increases for members of the *fad* regulon (encoding enzymes committed to fatty acid degradation), consistent with both of these fates. Strikingly, an elevated intracellular abundance of the human fatty acids palmitic acid and oleic acid, as well as the sterol cholesterol were observed. While these lipids are made by humans, they are not known to be made by bacteria^40^. We hypothesise that these sepsis pathogens were able to incorporate human cholesterol from serum, thereby remodelling their membranes.

### Interaction of human sepsis pathogens with cholesterol

**Fig. 8:**
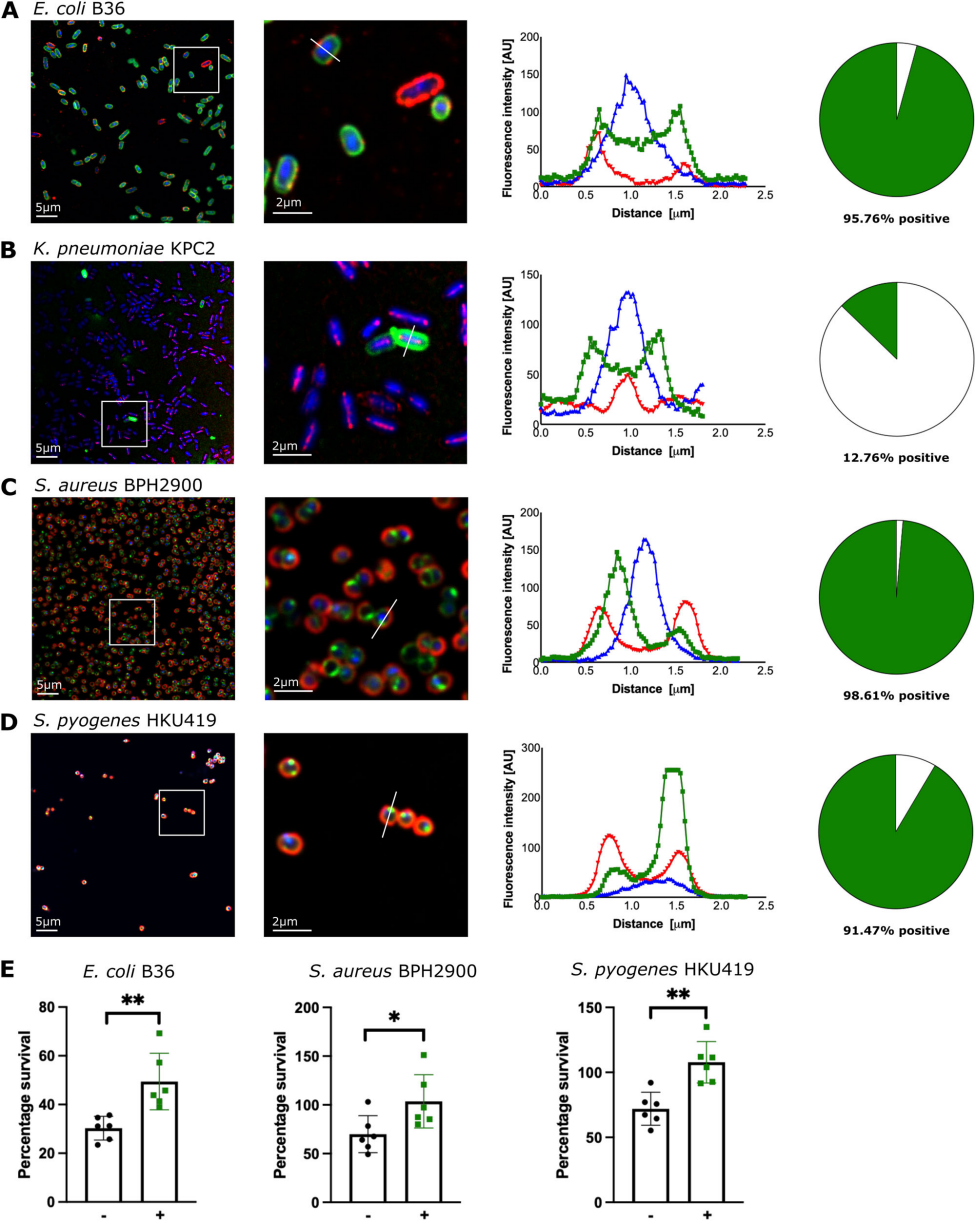
Quantification of the interaction of bacterial sepsis strains with cholesterol and protection from antimicrobial peptide killing. Each of the indicated bacterial strains was grown for 2 h in RPMI in the presence of 10 μM TopFluor-cholesterol (a fluorescent cholesterol analogue (**A**–**D**) or 10 μM cholesterol (**E**). **A**–**D** show an overview (left panel) and magnified view (adjacent panel) of *E. coli* B36 (first row), *K. pneumoniae* KPC2 (second row), *S. aureus* BPH2900 (third row) and *S. pyogenes* HKU419 (fourth row). TopFluor-cholesterol is shown in green, the bacteria are stained using different antibodies and alexa555 (red) and the nuclei are stained using DAPI (blue). The histogram of the fluorescence intensity of one representative bacterium is shown (third column). A representative cross-section for fluorescence analysis is provided from the magnified view panel. Pie graphs showing the percentage of the respective bacteria associated with cholesterol (green area, right column) from three independent experiments with the following total number of individual bacteria: *E. coli* B36 (*n* = 1045), *K. pneumoniae* KPC2 (*n* = 1169), *S. aureus* BPH2900 (*n* = 1140) and *S. pyogenes* HKU419 (*n* = 1047). **E** LL-37-mediated killing of *E. coli* B36, *S. aureus* BPH2900 and *S. pyogenes* HKU419 cells post cholesterol (10 µM) exposure. Strains were challenged at the LL-37 MIC (B36 = 32 µg/mL; BPH2900 = 512 µg/mL; HKU419 = 32 µg/mL) for 1 h in CA-MHB and percentage survival was determined by CFU enumeration. Error bars indicate the standard deviation of the mean from six biological replicates, *P < 0.05; **P < 0.01, two-sided Mann–Whitney test.

Our metabolomic data show increased cholesterol association with sepsis pathogens exposed to human serum. We speculated that this may be an important adaptation to growth in serum. Thus, to test this hypothesis, confocal microscopy was used with the fluorescently labelled cholesterol analogue TopFluor-cholesterol (TF-chol) (Fig. 8). For the Gram-negative bacteria *E. coli* B36 and *K. pneumoniae* KPC2, an incubation period of 2 h resulted in the close association of TF-chol with the bacterial cell wall (Fig. 8A, B). While we observed a >90% association of TF-chol with *E. coli* B36, we found that only 12% of *K. pneumoniae* KPC2 cells were associated with TF-chol (Fig. 8A and B). We also observed comparably high TF-chol association with the Gram-positive pathogens *S. aureus* BPH2900 and *S. pyogenes* HKU419 (>95% of cells, Fig. 8C, D). Time course experiments of bacterial growth in RPMI with supplemented TF-chol demonstrated that, over time, TF-chol enters the Gram-positive bacterial cell (Supplementary Fig. 13).

An alternative explanation for our finding of cholesterol in the pathogens exposed to human serum would be that the bacteria are actively taking up cholesterol as a carbon source. To rule out this possibility, growth was measured in the presence of 0.25 mM cholesterol and in the absence of glucose. No growth was detected for any of the four strains. While the adsorption of cholesterol onto the bacterial surface may be a happenstance of bacteria becoming exposed to human serum, it reflects a physiologically relevant scenario in sepsis. Any such remodelling of the bacterial membranes, even by passive adsorption, may have consequences in terms of the antimicrobial sensitivity of a pathogen that enters the blood of a patient. To address this concept, we investigated whether cholesterol associated with the strains affected susceptibility (as measured by minimal inhibitory concentration, MIC), to the human antimicrobial peptide LL-37. The MIC of LL-37 for *K. pneumoniae* KPC2 could not be determined (it was >1024 µg/mL). For the other three species, we observed that exposure of *E. coli* B36, *S. aureus* BPH2900, and *S. pyogenes* HKU419 to cholesterol significantly increased resistance to LL-37 (Fig. 8E).

The rise of acquired AMR in bacterial pathogens has been identified as a major human threat^41^. The poor economics for antibiotic development by pharmaceutical companies, combined with difficulties in identifying new drug targets, has limited the development of novel therapies for bacterial infections. While there is an attraction in blocking specific bacterial virulence factors to reduce collateral damage to the microbiome, such approaches tend to be pathogen-specific. Here, we took a systematic approach to identify the common transcriptomic, proteomic and metabolomic responses of four distinct sepsis pathogens as they entered a human serum environment. We observed that the four distinct pathogens acquired cholesterol from the serum and we show how this may augment survival against antimicrobial compounds produced by host innate immune cells, such as LL-37, providing a possible mechanism linked to enhanced innate immune resistance. This catalogue of serum-induced responses will provide a rich dataset for future mining as the search for novel targets intensifies to meet the challenges of AMR. Genes upregulated in sepsis pathogens following exposure to serum represent targets for future therapeutic development. Upregulated genes encoding surface proteins, such as the GAS arginine deiminase system, represent validated vaccine targets^42^. Furthermore, upregulated secreted proteins may find future utility as diagnostic targets if the sensitivity of antigen-capture systems can be sufficiently improved. In considering our findings, we note that sepsis involves a heterogeneous and dysregulated systemic host inflammatory response. Thus, future work should explore our findings in the context of human infection.

While the COVID-19 pandemic has focused the spotlight on infections as a major cause of morbidity and mortality, the most recent global analysis shows that in the 2019 pre-pandemic year approximately 5 million people died worldwide due to AMR-associated infections^43^. The problem of AMR and diseases such as sepsis will persist in an increasing background of fatal infections beyond the current COVID-19 pandemic, making the search for new bacterial therapeutic targets a continuing priority.

## Methods

### Bacterial culture

This research complies with all relevant ethical regulations. The University of Queensland Human Research Ethics Committee approved the use of human sera in this study (Approval Number: 2016000221). Bacterial strains were sourced in accordance with Westmead Hospital Human Research Ethics committee (HREC approval 2013/10/5.2(3835) and AU RED LNR/13/wmead/333). All bacterial strains were sourced from clinical specimens as outlined in Table 1 and prepared as glycerol stocks, stored at −80 °C, for use in all in vitro assays. *E. coli* strain MS14386 (ST224) was isolated in 2013 from the blood of a patient suffering from sepsis. *K. variicola* strain 04153260899A (ST355) was isolated from the blood of a patient suffering from urosepsis. All other strains have been described previously (Table 1). Biological replicates were generated starting from glycerol stocks of each strain. Pure colonies were obtained by incubating agar streak plates at 37 °C overnight, followed by an additional overnight preparatory broth culture at 37 °C in rich media with shaking at 200 rpm. Horse blood agar (*S. aureus, S. pyogenes*) or Lysogeny Broth agar (KpSC*, E. coli*) were used for streak plates, and Brain heart infusion (*S. aureus, S. pyogenes*) or Lysogeny Broth (KpSC*, E. coli*) were used for broth cultures. Bacterial cells were pelleted by centrifugation from 25 mL of overnight preparatory culture at 14,000×*g* at 4 °C for 10 min. Cells were resuspended in (i) 25 mL pooled human sera and incubated at 37 °C with shaking for 2 h, and (ii) 25 mL of Rosewell Park Memorial Institute (RPMI) 1640 medium at 37 °C with shaking for 2 h. RPMI was selected as the media as it produced characteristic growth curves for all four species and allowed standardisation of batched sample generation across the three omic platforms (i.e. transcriptomics, proteomics, and metabolomics). A total of 10 L of human serum was sourced from the Australian Red Cross Bloodbank. Sera samples were from multiple donors selected at random. Batched culture material for each strain and media combination were transferred to three separate 50 mL centrifuge tubes for the respective omic extractions; 5 mL culture for total RNA extraction, and 10 mL culture each for metabolite and protein extractions. For growth under osmotic, oxidative and iron starvation stress, bacteria (*E. coli B36*, *K. pneumoniae* KPC2, *S. aureus* BPH2900, *S. pyogenes* HKU419) were grown in nutrient broth (LB, *E*. coli and *K. pneumoniae*; BHI, *S. pyogenes* and *S. aureus*) to late exponential phase. Bacterial cells were collected by centrifugation and identical cell numbers used to inoculate fresh RPMI or serum (5 mL each) to a starting OD of 1.0. Bacterial cells were incubated at 37 °C for 2 h (as *per* the growth conditions described above). Cells were collected by centrifugation and cell numbers were determined by optical density. 4.0 × 10^8^ cells were used to inoculate (i) PBS control; (ii) PBS + 150 mM NaCl; (iii) PBS + 5 mM H_2_O_2_; and (iv) PBS + 5 mM deferoxamine (DF; iron chelator). Cells were incubated at 37 °C for 1 h. Viable cells were determined by serial dilution and plate count (LB plates for *E. coli* and *K. pneumoniae*, HBA plates for *S. pyogenes* and *S. aureus*). Assays were *n* = 6 biological replicates.

### Genomic DNA extraction, genome reconstruction and comparison

High molecular weight (HMW) genomic DNA (gDNA) was extracted using a phenol/chloroform protocol. Bacterial cells from 5 mL of overnight cultures were centrifuged at 2830×*g* for 2 min and resuspended in 2 mL PBS (pH 7.4). The resuspended cell pellets were incubated with added sodium dodecyl sulfate (20%) and RNase in a 57 °C water bath for 30 min. Proteinase K at 20 mg/mL was added to the solution and incubated at 57 °C for a further 30 min until the suspension cleared, indicating the bacterial cells were lysed. Eppendorf branded 15 mL Phase Lock tubes (5 PRIME) and an equal volume of phenol:chloroform was used to separate HMW gDNA by centrifugation at 2830×*g* for 2 min. The aqueous phase was transferred to a fresh Phase Lock tube and mixed with an equal volume of chloroform:isoamyl alcohol at a ratio of 24:1. A 1/10 volume of 3 M sodium acetate (pH 5.2) and 2.5 × volume of 100% (v/v) DNA-grade ethanol was added to precipitate gDNA. HMW gDNA was eluted in a final volume of 250 µL cross-linked 18 Ω H_2_O (MilliQ). A Gram-positive solution—consisting of Qiagen’s Gram-positive solution, lysozyme, and lysostaphin (10 mg/mL)—was included for the extraction of *S. aureus* and *S. pyogenes* gDNA. The Gram-positive solution was used prior to the addition of SDS and RNase solutions and incubated at 37 °C for 15 min. HMW gDNA was assessed for quality using pulse field gel electrophoresis, and sent to the Ramaciotti Centre for Genomics (Sydney, Australia) for long-read (PacBio RSII) and short-read sequencing (Illumina MiSeq). The 20 kb Template Preparation Using BluePippin™ Size-Selection System was used to select HMW gDNA fragments ~15–50 kb in size, and one sequencing cell *per* strain was used for sequencing on the PacBio RSII with P6C4 chemistry. Short-read sequencing libraries were prepared with the Illumina Nextera XT library preparation kit, and samples were sequenced using 2 × 150 bp paired-end sequencing on the Illumina MiSeq.

Complete genome assembly was undertaken using the approach outlined in Baines et al., for accurate and reproducible genome reconstruction^24^. Briefly, all genomic sequence data underwent quality control to ensure sufficient depth, basecall quality, and that the sequence data represented the expected organism based on a kraken2 analysis^44^. Using the consensus circular subreads, all genomes were assembled using four different approaches: (i) hybrid assembly with Unicycler v0.4.6^45^; (ii) a long-read-only assembly with Unicycler v0.4.6; (iii) a long-read-only assembly with Canu v1.8.0^46^ and; (iv) a long-read only assembly with HGAP3 pipeline, SMRTPortal v2.3.0^47^ using the parameters and settings outlined in Baines et al.^24^ Following assembly, all draft genomes were compared for structural consistency and a single assembly selected. The final assemblies were assessed for orientation to an appropriate start replicon and adjusted if required, then underwent a final error correction with the short-read sequence data using Snippy v4.3, run in an iterative manner until no variants were detected. Sequences representing plasmids were additionally checked for similarity to published sequences deposited in NCBI (https://www.ncbi.nlm.nih.gov/). Genome sequences were deposited in the European Nucleotide Archive using the accession numbers provided in Supplementary Data 11. As part of the upload process, genomes were transferred to GenBank and subject to the NCBI Prokaryotic Genome Annotation Pipeline for inclusion in Refseq (https://www.ncbi.nlm.nih.gov/genome/annotation_prok/).

In silico multi-locus sequence typing (MLST) was performed using mlst v2.19.0 (https://github.com/tseemann/mlst), employing the pubMLST schemes^48^. Antimicrobial resistance genes were detected using abriTAMR v0.2.2^49^ (https://github.com/MDU-PHL/abritamr), outputs filtered with minimum gene length and minimum nucleotide identify cut-off of 90%. In silico typing/serotyping was performed using emmtyper v0.1.0 (https://github.com/MDU-PHL/emmtyper) for *S. pyogenes*, Kleborate v2.0.1^50^ for KpSC, and the Center for Genomic Epidemiology’s (http://www.genomicepidemiology.org/) spaTyper and SeroTypeFinder for *S. aureus* and *E. coli*, respectively. Virulence genes were detected with ABRIcate (https://github.com/tseemann/abricate) or Kleborate v2.0.1 (for KpSC), requiring a ≥90% length and identity match.

Phylogenetic analysis was performed using Mashtree 1.1.2^51^ with sketch sizes of 50,000 kmers. For context, all “complete” or “chromosome” level assemblies in GenBank (as of June 2020) matching the investigation species were included. The resulting trees were visualised in FigTree 1.4.4 (https://github.com/rambaut/figtree/).

Hierarchical orthologue clustering was performed using pirate v1.0.2^52^ using the chromosome protein-coding sequences (CDS) for all 20 genomes (available in the respective Refseq records, Supplementary Data 11), and clustering at steps of 50%, 60%, 70%, 80%, and 90% amino acid identity. The output was filtered based on presence/absence to identify those clusters conserved across a single species (Supplementary Data 1–4), or across all 20 genomes (Supplementary Data 5). The intersection of orthologue clusters across species was visualised using UpSet plots, generated in R v3.6.3 with the programme “UpSetR” (https://github.com/hms-dbmi/UpSetR).

### Functional annotations of genomes and enrichment analysis

Protein sequences derived from the 20 bacterial isolates were matched to their closest homologue in the Uniprot database using BLASTP, keeping only matches with *E*-value < 1e−05. GO terms for every protein were annotated by mapping the UniProt ID to the GO database with Blast2GO^53^, by querying the eggNOG database v5.0 (downloaded 6/11/2020) with eggnog-mapper^54^ and using the PANNZER2 webserver^55^. The following parameters were used for querying the eggNOG database: seed ortholog evalue 0.001, seed ortholog score 60, query coverage 80, and subject coverage 80. Only annotations with Positive Predictive Value (PPV) > 0.7 were kept from PANNZER2 annotation results. KEGG terms were retrieved from eggNOG and KEGG Automatic Annotation Server (KAAS)^56^. Genes and proteins corresponding to the core or accessory genomes were annotated from pirate v1.0.2 results. To obtain a comprehensive annotation, all functional terms retrieved were combined and redundant annotations from different databases were deduplicated. The resulting 3,342,890 annotations are available in Supplementary Data 8. Gene and protein set enrichment analysis was performed with package clusterProfiler v3.16^57^ and visualisation with the package ggplot2 in R^58^.

### Total RNA extraction and sequencing

Total RNA was extracted from *S. aureus*, *S. pyogenes* and *E. coli* using a trizol/chloroform approach and commercially available extraction kits with key modifications to manufacturer instructions. The commercial kits used were RNeasy mini kit (Qiagen), RNeasy miniElute Kit (Qiagen), RNase-Free DNase set (Qiagen), and Fast RNA Pro Blue Kit (QBiogen). Briefly, modifications to the manufacturer’s protocol included the use of 5 mL of culture (RPMI or pooled human sera) which was stabilised in 10 mL RNAprotect Bacteria reagent (Qiagen) and incubated at room temperature (RT) for 10 min prior to centrifugation at 3220×*g* for 20 min (RT). Bacterial cells were transferred to a tube containing Lysing Matrix B (Fast RNA Pro Blue Kit, QBiogen) and mechanically lysed at 6000 rpm for 40 s (Precellys 24 homogeniser, Bertin Instruments). The aqueous phase of the post-mechanically lysed solution was incubated with 1:10 volume 3 M sodium acetate (pH 5.2), and 2.5× volume of 100% ice-cold ethanol at −20 °C overnight to precipitate total RNA. The overnight solution was treated with DNase to remove contaminating DNA, and RNA was eluted using the RNeasy miniElute kit (Qiagen). Total RNA was eluted in a final volume of 34 µL. A modified trizol/chloroform extraction protocol was used to extract total RNA from KpSC strains. Briefly, 1 mL of culture material was stabilised in 2 mL of RNA stabilisation solution (95% ethanol:5% phenol at pH 4.3) containing 2.5 M guanidine thiocyanate salt (GTC; Sigma, Reference G6639). Two millilitre screw-top centrifuge tubes containing 100 mL of 0.1 mm diameter zirconia/silica beads (BioSpec, Reference 11079101z) were used for mechanical lysis by vortex for 10 min at 4 °C. Total RNA was eluted in 50 µL of DEPC-treated water. Extracted total RNA samples were sent to the Australian Genome Research Facility (Melbourne, Australia) for ribosomal RNA depletion using the Ribo-Zero rRNA Removal Kit for bacteria (Illumina), and library preparation using the TruSeq Stranded mRNA Library Preparation Kit (Illumina) following manufacturer’s protocol; note the poly(A) RNA purification step in the TruSeq kit was excluded in our workflow. The quality of RNA samples was assessed using an Agilent 2100 Bioanalyzer following the manufacturer’s protocol. Transcriptomic sequencing was performed on a single Illumina HiSeq2500 lane with 100 bp paired-end chemistry and dual indexing.

### RNAseq analysis

Read abundance for each strain and condition (growth in RPMI versus serum exposure) was quantified using the resulting RNAseq Illumina fastq files, the finished genome assembly and annotation for each strain as a reference, the read aligner minimap2 v2.17-r94^59^, and strand-specific read counting onto every gene using featureCounts v2.0.1^60^. Quality control was performed by assessing total mapped reads, count *per* million distribution pre- and post-normalisation, MDS plots, hierarchical clustering of the count correlation matrix and Cook’s distance distributions of counts (Supplementary Fig. 1). From this, one biological replicate (among the six replicates *per* condition) was discarded as an outlier from four *E. coli* and two *S. aureus* RNAseq experiments (the replicate samples discarded were: *E. coli* B36 RPMI #5, *E. coli* MS14384 RPMI #1, *E. coli* MS14386 SERA #1, *E. coli* MS14387 RPMI #1, *S. aureus* BPH2760 SERA #4, *S. aureus* BPH2947 SERA #4). Differential gene expression analysis was performed with the R package edgeR using generalised linear model^61^. Gene expression was considered statistically significant by applying threshold limits of 2× fold change and a *P* value of <0.05 (adjusted for false discovery).

### Protein extraction and proteomic analysis

Bacterial cells were collected by centrifugation at 14,000×*g* at 4 °C for 15 min and resuspended in 1 mL ice-cold 1× PBS. Wash steps were repeated twice before storing at −80 °C until further extraction. Cells were resuspended in 150 mM Tris–HCl, 125 mM NaCl and 0.1 mm acid-washed glass beads (Sigma) and lysed by 4–6 rounds of bead-beating (5 m/s, 1 min) with 1 min rest periods on ice. Insoluble material was removed by centrifugation for 3 min at 4 °C at 4000×*g*. 250 µL was mixed with ice-cold dH_2_O:methanol:chloroform (ratio of 3:4:1) to precipitate proteins. Proteins were collected by centrifugation and washed twice with methanol prior to solubilisation in 8 M guanidine–HCl, 100 mM HEPES, pH 7.6. Reduction and alkylation were performed with 10 mM dithiothreitol (DTT) and 20 mM iodoacetamide (IAA) for 1 h each, respectively. Proteins were digested with sequencing grade modified trypsin (Promega, Madison, WI), at a protein:protease ratio of 30:1 overnight at 37 °C. The resulting peptides were desalted by solid phase extraction using hydrophilic-lipophilic balance (HLB) cartridges (Waters, Bedford, MA) according to the manufacturer’s instructions. Extractions were split and lyophilised, with one aliquot shipped to the Australian Proteome Analysis Facility (Sydney, Australia), and the second to Monash Proteomics & Metabolomics Facility (Melbourne, Australia) for data-independent acquisition (DIA/SWATH) and data-dependent acquisition (DDA) mass spectrometry, respectively.

For DDA analysis, the lyophilised peptides were resuspended at 2 μg/μL in 2% MeCN, 0.1% TFA with spiked iRT peptides using a PCV-3000 (5 s spin 500×*g*, 10 s vortex, 30 cycles). Samples were centrifuged at 16,100×*g* for 5 min (Centrifuge 5415D, Eppendorf) and supernatants vialled for MS analysis. One μL of each sample was analysed on a Thermo LC–MS system comprised of an Ultimate 3000 RSLC system coupled to an Orbitrap QExactive Plus mass spectrometer. The samples were loaded onto a trap column (Acclaim PepMap 100, 100 μm × 2 cm, Thermo) at 15 μL/min followed by separation on an Acclaim PepMap RSLC column (75 μm × 50 cm, Thermo) at 250 μL/min using a 2 h gradient from 6% to 30% MeCN in 0.1% formic acid. MS analysis consisted of a Top12 method (MS scans: 375–1575*m/z*, 70k resolution; MSMS scans: 17.5k resolution, 1.8*m*/*z* isolation window, 27 normalised collision energy (NCE), charge state 2–5, peptide match preferred, 15 s dynamic exclusion). Strain-based samples were acquired in a randomised order with interleaved wash cycles. The open-source software package MaxQuant (v1.5.5.1)^62^ was used to identify and quantify proteins within each sample batch using strain-specific databases. MaxQuant outputs were visualised and analysed using the Perseus software suite (v1.5.8.5)^63^ and LFQ-Analyst^64^. Supplementary Data 12 highlights the percentage of proteins accurately quantified per strain.

For DIA/SWATH analysis, the lyophilised peptides were resuspended in the loading buffer (2% MeCN, 0.1% formic) to a final concentration of 1 µg/µL. Probe sonication (2 × 30 s) was performed with rest periods on ice between rounds, followed by centrifugation at 14,100×*g* for 5 min. Two µL of supernatant (5.5 µL for *S. aureus*) was taken for analysis by DIA-SWATH. Samples were further diluted with the loading buffer to a final volume of 10 µL, and analysed on a 6600 TripleTOF mass spectrometer (Sciex, Framingham, MA) coupled to an Eksigent Ultra-nanoLC-1D system (Eksigent Technologies, Dublin, CA). Peptides were loaded onto a trap column packed in-house (Solidcore Halo® 2.7 µm 160 Å ES-C18 (Advanced Materials Technology), 150 µm × 3.5 cm at 4 µL/min for 10 min, followed by separation on an analytical column packed in-house (Solidcore Halo® 2.7 µm 160 Å ES-C18 (Advanced Materials Technology), 200 µm × 15 cm) for 120 min at 600 nL/min using the mobile phase buffer containing 99% MeCN in 0.1% formic acid, with its gradient increased from 2% to 33%. For SWATH-MS experiments, first, a TOFMS survey scan was acquired (*m/z* 350–1500; 0.05 s) then the 100 predefined *m/z* ranges were sequentially subjected to MS/MS analysis. MS/MS spectra were accumulated for 30 ms in the mass range *m/z* 350–1500.

### Metabolite extraction and metabolomic analysis

Metabolite profiles for the 20 strains investigated were collected on two MS platforms. Comprehensive targeted profiling was performed for trimethylsilylated metabolites using a GC-QQQ instrument, where multiple reaction monitoring (MRM) transitions were targeted for ~350 metabolites. Samples were also analysed by hydrophilic interaction liquid chromatography (HILIC)–LC–QTOF MS, to enable the broadest coverage of polar metabolites. For the LC–MS analysis, ~600 authentic metabolite standards were run concurrently to enable absolute identification. Each methodology was able to annotate 120–200 metabolites within each species dataset, for which the integrated area responses were recorded into data matrices^65^.

Bacterial cells from RPMI and pooled human sera samples were metabolically arrested by infusing 3× volume cold PBS in 50 mL Eppendorf-branded centrifuge tubes and incubated in an ice/water slurry for 15 min. Cell pellets were resuspended in cold PBS after centrifugation at 805×*g* for 10 min and at 1 °C, and transferred to 1.5 mL Eppendorf-branded centrifuge tubes. Cells were washed 3× with cold PBS and sent to Metabolomics Australia (Melbourne, Australia) on dry ice for further extraction and processing.

### Metabolomics sample extraction

All samples were randomised prior to metabolite extraction. Cells were resuspended in 1 mL extraction solution containing internal standards consisting of methanol/water at 3:1 v/v ratio with 3 nmol^13^C_5_^15^N-Valine, 5 nmol^13^C_6_-Sorbitol, 2 nmol 1,2-^13^C_2_-Myristic acid (Cambridge Isotopes). Resuspended cells were subjected to freeze–thaw cycles of 15 s in liquid nitrogen, followed by 15 s in a dry ice/ethanol bath for 10 cycles to facilitate cell lysis. Samples were centrifuged at 17,600×*g* for 5 min at 1 °C to remove cell debris and cell lysate was transferred into fresh tubes. A further aliquot of cell lysate was aggregated into a tube to create a pooled biological quality control (PBQC) for monitoring the stability and reproducibility of metabolites during data acquisition.

### Gas chromatography-triple quadrupole mass spectrometry (GC–QqQ–MS)

Aliquots of 400 µL cell lysates and PBQC were transferred into glass inserts and dried completely in a rotational vacuum concentrator (RVC 2–33, John Morris Scientific) at 20 °C. To completely remove residual water, all samples were washed with 3 × 50 μL methanol. Glass inserts were transferred into 2 mL autosampler vials, followed by online derivatization with an autosampler robot (PAL RTC). All samples were methoximated with 25 μL of methoxylamine hydrochloride solution (30 mg/mL in pyridine, Sigma) for 2 h at 37 °C, followed by trimethylsilylation in 25 μL of N,O-Bis(trimethylsilyl)trifluoroacetamide containing trimethylchlorosilane (BSTFA + 1% TMCS) for 1 h at 37 °C with continuous mixing, and incubated in room temperature for 1 h prior to GC–QqQ–MS analysis. All samples were analysed using a GCMS-TQ8040 (Shimadzu) equipped with a J&W DB-5 capillary column (30 m × 0.25 mm, 1.00 μm film thickness, Agilent Technologies). The inlet temperature was kept at 280 °C and helium was used as a carrier gas (purge flow = 5.0 mL/min, column flow = 1.1 mL/min). An aliquot of 1 μL derivatized sample was injected into the GC–QqQ–MS with an injection split ratio of 1:10. The GC oven temperature was ramped from 100 °C, at which it was initially held for 4 min, to 320 °C at 10 °C/min, and then held for 11 min at 320 °C. The transfer line and ion source temperatures were 280 and 200 °C, respectively. Argon was used as the collision-induced dissociation gas. Shimadzu GCMSsolutions Realtime Analysis (version 4.42) enabled target metabolite detection through the utilisation of the Smart Metabolites Database v3 (Shimadzu), which contains up to 467 targets with MRM transitions including precursor ion, product ion, collision energy, retention index and time, with a minimal dwell time of 2 ms setup for the acquisition method. The automatic adjustment of retention time (AART) in GCMSsolution software (version 4.42, Shimadzu) and a standard alkane series mixture (C7-C33, Restek) were used to correct retention time shifts in the acquisition method when the column is cut or replaced. Peak area integration and curated data matrix generation were performed using Shimadzu LabSolutions GC–MS browser software (version 4.42).

### High-performance liquid chromatography–quadrupole time of flight mass spectrometry (HPLC–Q-TOF–MS)

Aliquots of 50 μL cell extracts and PBQC samples were transferred into 2 mL autosampler vials with glass inserts. A mixture of 550 authentic standards (in 13 separate mixes), covering a range of metabolic pathways was run with each batch of bacterial species to aid metabolite identification (MSI level 1). Analysis was performed on the Agilent 1200 series HPLC system coupled with a 6545 Q-TOF MS using Agilent MassHunter Workstation LC/MS Data Acquisition for 6200 series TOF/6500 series Q-TOF (version 10.1) as follows^66^: Samples were maintained at 4 °C in the autosampler and metabolite separation was performed by injecting 7 µL of sample into a SeQuant® ZIC-pHILIC PEEK coated column (150 mm × 4.6 mm, 5 µm polymer, Merck) maintained at 25 °C, with a binary gradient made up of solvent A (20 mM ammonium carbonate, pH 9.0, Sigma-Aldrich) and solvent B (100% MeCN, Hypergrade for LCMS LiChrosolv, Merck) at a flow rate of 300 µL/min. A 33.0 min gradient was set up with time (*t*) = 0 min, 80% B; *t* = 0.5 min, 80% B; *t* = 15.5 min, 50% B; *t* = 17.5 min, 30% B; *t* = 18.5 min, 5% B; *t* = 21.0 min, 5% B; *t* = 23.0 min, 80% B.

Metabolites were ionised in an electrospray ionisation source (ESI) with a capillary voltage of 2.5 kV, drying nitrogen gas flow at 10 L/min, with the temperature and nebuliser pressure of 225 °C and 20 psi, respectively. The voltages were set at −125 V for fragmentor and at −45 V for skimmer. The acquisition was conducted with a scan range of 85–1200*m*/*z* at a rate of 1.5 spectra/s in negative all-ion fragmentation (AIF) mode, which included three collision energies (0, 10, 20 V). A QTOF reference mass solution with 2.5 µM Hexakis(1H, 1H, 3H-tetrafluoropropoxy)phosphazine and 5 µM purine in 95:5 acetonitrile: water (API-TOF Reference Mass Solution Kit, Agilent Technologies) was continuously infused into the ESI source for internal mass calibration during data acquisition. In accordance with the metabolomics standards initiative (MSI), metabolite identification (MSI level 1) was based on the retention time and molecular masses matching to an authentic standard^67^. Peak area integration was performed using MassHunter TOF Quantitative Analysis software (version B.07.00, Agilent Technologies) using the Metabolomics Australia In-house Data processing pipeline.

### Confocal microscopy

Microscopy of *E. coli* B36, *K. pneumoniae* KPC2, *S. aureus* BPH2900 and *S. pyogenes* HKU419 was performed as previously described^68^. Briefly, bacteria were cultured overnight at 37 °C on horse blood agar plates, then diluted in 10 mL RPMI to an OD_600nm_ = 0.1 and incubated for the indicated times with a final concentration of 10 μM TopFluor-Cholesterol (Cat. No. 810255P, Sigma; from a 10 mM stock in 100% DMSO) at 37 °C under stationary conditions. Samples were centrifuged, washed twice with PBS, resuspended in 100 μL PBS, and seeded onto sterile glass coverslips coated with poly‐l‐lysine in 24‐well cell culture plates. After air-drying for 30 min protected from light, excess liquid was discarded, and bacteria were fixed at RT with 4% paraformaldehyde (PFA; Electron Microscopy Sciences; diluted in PBS). Bacteria were washed three times with 0.1% BSA in PBS and subsequently incubated with the following primary antibodies for 1 h, protected from light at RT: *E. coli*: O25 monospecific O rabbit antiserum (85022, SSI Diagnostica); *K. pneumoniae*: monoclonal antibody raised in BALB/c mice against the sarkosyl-insoluble outer membrane fraction of a non-mucoid strain of *Klebsiella pneumoniae* B5055. Fab fragments of this antibody were produced by digestion on immobilised papain (Pierce) and purification on a protein A Sepharose column (Pierce)^69^; *S. aureus*: polyclonal antibody serum were raised in rabbits against whole fixed cells of *Staphylococcus aureus* USA300 strains, BPH2919 and BPH3672 (WEHI antibody technology platform, https://www.wehi.edu.au/research/research-technologies/antibody-technologies); *S. pyogenes*: rabbit *Streptococcus* Group A polyclonal (Cat. No. PAB13831, Abnova). Antibodies were used at 1:1000 dilution in 3% bovine serum albumin (BSA) in PBS for *K*. *pneumoniae*, or 0.1% BSA in PBS for *E. coli*, *S. aureus* and *S. pyogenes*. Samples were washed three times with 0.1% BSA in PBS and incubated for 1 h at RT protected from light with a goat anti-mouse Alexa Fluor555 (Cat. No. A28180, Thermo Fisher Scientific), or a goat anti-rabbit Alexa Fluor555 (Cat. No. 4413s, Cell signalling), secondary antibody at 1:5000 dilution in 0.1% BSA in PBS. Samples were washed three times with 0.1% BSA in PBS, stained for 10 min with DAPI (1:1000 in PBS), washed three times (0.1% BSA in PBS) and subsequently mounted in DAKO fluorescence mounting medium (Cat. No. CS70330-2, DAKO North America Inc.). The samples were analysed with a Leica DMi8 inverted confocal laser scanning microscope (HC PL APO CS2, objective ×100/1.4 oil; Leica Microsystems). Images were processed and exported with Fiji/ImageJ (https://imagej.nih.gov/ij/) or Imaris Viewer software (https://imaris.oxinst.com/imaris-viewer).

### Determination of LL-37 MIC and cell killing

Direct colony suspensions of *E. coli* B36, *S. aureus* BPH2900 and *K. pneumoniae* KPC2 in RPMI, and *S. pyogenes* HKU419 in a chemically defined medium were pre-incubated for 2 h in the presence or absence of 10 µM cholesterol (cholesterol stock dissolved in ethanol to 10 mM). Bacterial cells were harvested by centrifugation at 4000×*g* for 10 min, washed in 1 mL Cation-Adjusted Mueller Hinton broth (CAMHB) (4000×*g*, 2 min), and diluted in CAMHB to OD_600_ 0.001. LL-37 MICs were then undertaken by broth microdilution as per Clinical and Laboratory Standards Institute guidelines^70^. For determination of LL-37 killing at the MIC, aliquots (10 µL) of bacterial suspensions were taken at 0 and 1 h, serially diluted in PBS and plated on LB agar (*E. coli* B36, *S. aureus* BPH2900 and *K. pneumoniae* KPC2) or Thayer–Martin agar (*S. pyogenes* HKU419) for colony forming unit (CFU)/mL determination and calculation of percentage survival. Assays were repeated for six biological replicates.

### Construction and testing of GAS mutants

GAS mutant 5448Δ*arcC* was generated by deletion replacement, as previously described^71^. Briefly, to construct the 5448Δ*arcC* insertional mutant, the central 300 bp region of the *arcC* gene was PCR amplified and recovered by TA cloning into pCR2.1-TOPO (Invitrogen), and subsequently cloned into the temperature–sensitive erythromycin (Erm) resistant plasmid pHY304^71^. The resulting plasmid was transformed into GAS 5448 and plated on THY agar-Erm (5 μg/mL) for 2 days at 30 °C. Single crossover recombination events were identified by shifting to the non-permissive temperature (37 °C) while maintaining Erm selection. Complementation of 5448Δ*arcC* with wild-type *arcC* was undertaken using the highly efficient plasmid (plZts) for creating markerless isogenic mutants^72^. *arcC* gene deletion replacement and *arcC* gene complementation was confirmed by DNA sequence analysis (Australian Equine Genome Research Centre, University of Queensland, Brisbane, Australia). All PCR primer sequences are provided in Supplementary Table 1. To investigate the growth of GAS wildtype and mutants in human serum, overnight cultures of wild-type GAS strain 5448, carbamate kinase deficient 5448Δ*arcC*, and 5448Δ*arcC::arcC* were washed in 1 × PBS and standardised to OD600 nm = 0.0025 in human serum. Bacteria were grown in a 96-well plate at a final volume of 200 μL and measured at 600 nm using a FLUOstar Omega microplate reader (BMG Labtech) at 37 °C with orbital shaking for 30 sec prior to each measurement. Growth assays were performed in biological triplicates and measured in technical triplicates. Area-under-the-curve (AUC) was calculated using the R package *Growthcurver* to compute AUC. Significance testing was performed using Student’s unpaired *t*-test, with the null hypothesis (no difference between mean AUC values) rejected for *p* < 0.05.

### Construction and testing of *E. coli* mutants

*E. coli* mutants were constructed using λ-Red recombineering with some modifications as previously described^73,74^. Primers used to construct these mutants are listed in Supplementary Table 1. To investigate *E. coli* survival in human serum, overnight cultures of *E. coli* B36, B36Δ*carB*, B36Δ*wcaF*, EC958, EC958Δ*carB* and EC958Δ*wcaF* were standardised to OD600 nm = 2.0 in LB medium. Standardised cultures were then pelleted and resuspended in an equal volume of human serum (final OD600 nm = 2.0). Bacteria were then incubated in a 96-well plate at a final volume of 100 μL and measured at 600 nm using a FLUOstar Omega microplate reader (BMG Labtech) at 37 °C with shaking and orbital reading settings. Non-inoculated serum was used as a reference blank. Assays were performed in biological triplicates and measured in technical triplicates.

### Genome sequence confirmation of *S. aureus* transposon mutants and growth in human serum

To confirm that the *S. aureus* NE137 transposon mutant contained only the insertion in *isdI* and no additional mutations compared to *S. aureus* JE2 wild type, genomic DNA was extracted and subjected to Oxford Nanopore Sequencing on a gridION platform using R10.4 (FLO-MIN112) chemistry. On-board base-calling was performed using Guppy v.6.1.5 in super-accurate mode and yielded 364,182 reads with a total length of 1,012,245,822 bp resulting in 352× coverage. The NE137 genome was assembled using the Trycycler^75^  recommended workflow (https://github.com/rrwick/Trycycler/wiki) to generate a high-quality consensus long-read assembly. The resulting assembly was compared to the JE2 reference genome (NZ_CP020619.1) using the Mauve whole-genome aligner^76^ implemented in *Geneious Prime* (v. 2022.2.2). Prior to growth assessment, human serum (Male AB plasma, Sigma Aldrich) was clarified by centrifugation at 3000×*g* for 3 min and heat-inactivated at 56 °C for 30 min. One mL of overnight cultures of *S. aureus* USA300 JE2 WT and JE2::*isdI* transposon mutant^77^ grown under agitation in BHI broth (Bacto BD) were centrifuged at 5000×g for 5 min and the bacterial pellets washed twice with sterile PBS. Bacteria were resuspended in 1 mL of RPMI 1640 medium (without glutamine) and standardised to OD_600nm_ = 0.5 in RPMI 1640. Bacterial inocula corresponding to OD_600nm_ = 0.05 were then incubated in RPMI 1640 in 50% treated serum (v/v), or in RPMI 1640 only, at a final volume of 200 μL and their growth was measured every 10 min at 600 nm using a ClariostarPLUS microplate reader (BMG Labtech) at 37 °C with double orbital shaking at 300 rpm, 20 s prior every measurement. Growth assays in RPMI 1640 with 50% treated serum (v/v) and in RPMI 1640 only were performed within the same plate in technical triplicates and the experiments were performed in independent biological triplicates. Area-under-the-curve (AUC) was calculated using the R package *Growthcurver* to compute AUC. Significance testing was performed using Student’s unpaired *t*-test, with the null hypothesis (no difference between mean AUC values) rejected for *p* < 0.05.

### Reporting summary

Further information on research design is available in the Nature Portfolio Reporting Summary linked to this article.

## Supplementary information

Supplementary Information

Peer Review File

Description of Additional Supplementary Files

Supplementary Data 1

Supplementary Data 2

Supplementary Data 3

Supplementary Data 4

Supplementary Data 5

Supplementary Data 6

Supplementary Data 7

Supplementary Data 8

Supplementary Data 9

Supplementary Data 10

Supplementary Data 11

Supplementary Data 12

Reporting Summary

## Data Availability

All omic datasets generated during this study are publicly available. The primary accession codes for genomics, transcriptomics, proteomics, and metabolomics are listed in parentheses for each bacterial species: *Escherichia coli* (PRJEB29930; GSE152966; GSE152967; GSE152968; GSE152969; PXD016840; PXD020545; MTBLS2015), *Klebsiella pneumoniae* species complex (PRJEB29928; GSE152839; GSE1528340; GSE152843; GSE152844; GSE152847; GSE152964; PXD016672; PXD020839; MTBLS2322), *Staphylococcus aureus* (PRJEB29881; GSE152833; GSE152834; GSE152835; GSE152837; GSE152838; PXD016504; PXD020791; MTBLS1898), and *Streptococcus pyogenes* (PRJEB29800; GSE152821; GSE152822; GSE152823; GSE152824, GSE152826; PXD016913; PXD020863; MTBLS2324). Data were deposited to the following public repositories: RefSeq, Gene Expression Omnibus, Proteomics Identifications Database, and MetaobLights. Details of the corresponding accession numbers for each strain can be found in Supplementary Data 11. Source data are provided with this paper.

## Source data

Source Data
