# Supplementary material for: Integrative omics identifies conserved and pathogen-specific responses of sepsis-causing bacteria
Source: Nat Commun. 2023 Mar 18;14:1530. doi: 10.1038/s41467-023-37200-w (PMC10024524; doi:10.1038/s41467-023-37200-w)
Supplement: Supplementary file 1 — Supplementary Information [file 41467_2023_37200_MOESM1_ESM.pdf]

Integrative omics identifies conserved and pathogen-specific responses of sepsis-causing bacteria

Supplementary Figures and Tables

Andre Mu[^,1,2], William P. Klare[^,3], Sarah L. Baines[^,1], C.N. Ignatius Pang[^,4,5], Romain Guerillot[^,1], Nichaela Harbison-Price[^,6,7], Nadia Keller[6], Jonathan Wilksch[1], Nguyen Thi Khanh Nhu[6,7], Minh-Duy Phan[6,7], Bernhard Keller[6,7], Brunda Nijagal[8,9], Dedreia Tull[8], Saravanan Dayalan[8], Hwa Huat Charlie Chua[8], Dominik Skoneczny[8], Jason Koval[4], Abderrahman Hachani[1], Anup D. Shah[10], Nitika Neha[10], Snehal Jadhav[10], Sally R. Partridge[11], Amanda J. Cork[6,7], Kate Peters[6,7], Olivia Bertolla[6,7], Stephan Brouwer[6,7], Steven J. Hancock[6], Laura Álvarez-Fraga[6], David M.P. De Oliveira[6,7], Brian Forde[6], Ashleigh Dale[3], Warasinee Mujchariyakul[1], Calum Walsh[1], Ian Monk[1], Anna Fitzgerald[12], Mabel Lum[12], Carolina Correa-Ospina[4], Piklu Roy Chowdhury[13], Robert G. Parton[7,14], James De Voss[6], James Beckett[6], Francois Monty[15], Jessica McKinnon[13], Xiaomin Song[16], John R. Stephen[17], Marie Everest[17], Matt I. Bellgard[18], Matthew Tinning[17], Michael Leeming[8], Dianna Hocking[1], Leila Jebeli[1], Nancy Wang[1], Nouri Ben Zakour[11], Serhat A. Yasar[4], Stefano Vecchiarelli[4], Tonia Russell[4], Thiri Zaw[16], Tyrone Chen[19], Don Teng[8,9], Zena Kassir[4], Trevor Lithgow[20], Adam Jenney[20], Jason N. Cole[21], Victor Nizet[21], Tania C. Sorrell[11], Anton Y. Peleg[20], David L. Paterson[22], Scott A. Beatson[6], Jemma Wu[16], Mark P. Molloy[16], Anna E. Syme[23], Robert J.A. Goode[10,24], Adam A. Hunter[18], Grahame Bowland[18], Nicholas P. West[*,6], Marc R. Wilkins[*,4], Steven P. Djordjevic[*,13], Mark R. Davies[*,1], Torsten Seemann[*,1], Benjamin P. Howden[*,1], Dana Pascovici[*,16], Sonika Tyagi[*,19], Ralf B. Schittenhelm[*,10], David P. De Souza[*,8], Malcolm J. McConville[*,8,9], Jonathan Iredell[*,11], Stuart J. Cordwell[*,3], Richard A. Strugnell[*,1], Timothy P. Stinear[*,1], Mark A. Schembri[*,6,7], Mark J. Walker[*,+,6,7]

[^,*] **Contributed equally to this work**

[+]**Corresponding author:** Professor Mark J. Walker, Institute for Molecular Bioscience, The University of Queensland, Brisbane, Queensland, Australia; mark.walker@uq.edu.au

Author addresses:

[1]Department of Microbiology and Immunology, The University of Melbourne at the Peter Doherty Institute for Infection and Immunity, Melbourne, Victoria, Australia

[2]Wellcome Sanger Institute, Hinxton, United Kingdom

[3]Charles Perkins Centre and School of Life and Environmental Sciences, The University of Sydney, Sydney, New South Wales , Australia

[4]Ramaciotti Centre for Genomics, School of Biotechnology and Biomolecular Sciences, University of New South Wales, Sydney, New South Wales, Australia

[5]Bioinformatics Group, Children's Medical Research Institute, Faculty of Medicine and Health, The University of Sydney, New South Wales, Australia

[6]Australian Infectious Diseases Research Centre and School of Chemistry and Molecular Biosciences, The University of Queensland, Brisbane, Queensland, Australia

[7]Institute for Molecular Bioscience, The University of Queensland, Brisbane, Queensland, Australia

[8]Department of Biochemistry and Molecular Biology, Bio21 Molecular Science and Biotechnology Institute, University of Melbourne, Melbourne, Victoria, Australia

[9]Metabolomics Australia, Bio21 Institute, The University of Melbourne, Melbourne, Australia

[10]Monash Proteomics and Metabolomics Facility, Monash Biomedicine Discovery Institute, Monash University, Melbourne, Victoria, Australia

[11]Centre for Infectious Diseases and Microbiology, Westmead Hospital/ Westmead Institute, and Sydney ID, University of Sydney, New South Wales, Australia

[12]Bioplatforms Australia Ltd., Sydney, New South Wales, Australia

[13]Australian Institute for Microbiology and Infection, University of Technology Sydney, New South Wales, Australia

[14]Centre for Microscopy and Microanalysis, The University of Queensland, Brisbane, Queensland, Australia

[15]Australian Biocommons, The University of Melbourne, Melbourne, Victoria, Australia

[16]Australian Proteome Analysis Facility, Macquarie University, Sydney, Australia

[17]Australian Genome Research Facility Ltd., Melbourne, Victoria, Australia

[18]Center for Comparative Genomics, Murdoch University, Perth, Western Australia, Australia

[19]Department of Infectious Diseases, Monash University, Melbourne, Victoria, Australia

[20]Centre to Impact AMR and Infection Program, Monash Biomedicine Discovery Institute and Department of Microbiology, Monash University, Melbourne, Victoria, Australia

[21]Department of Pediatrics, University of California at San Diego School of Medicine, La Jolla, CA 92093, USA; Skaggs School of Pharmaceutical Sciences, University of California at San Diego, La Jolla, CA 92093, USA.

[22]Centre for Clinical Research, The University of Queensland, Brisbane, Queensland, Australia

[23]Melbourne Bioinformatics, The University of Melbourne, Melbourne, Australia

[24]Present Address: Commonwealth Scientific and Industrial Research Organisation, Clayton, Australia

Supplementary Figures and Tables

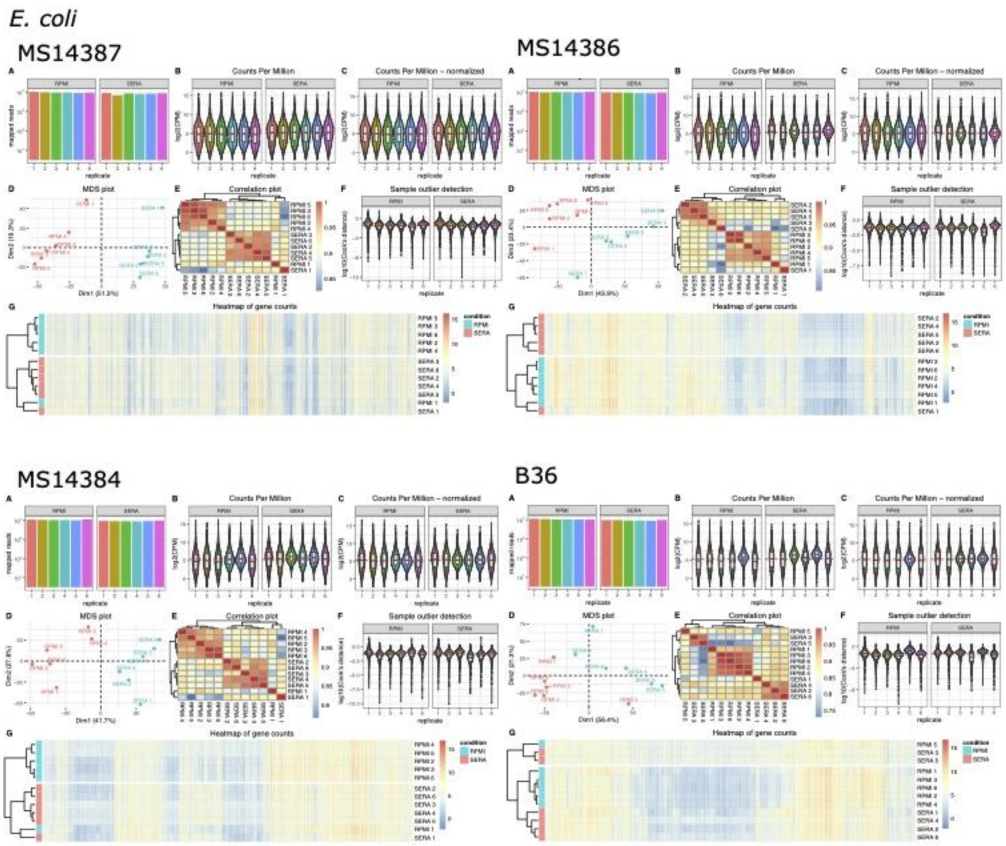

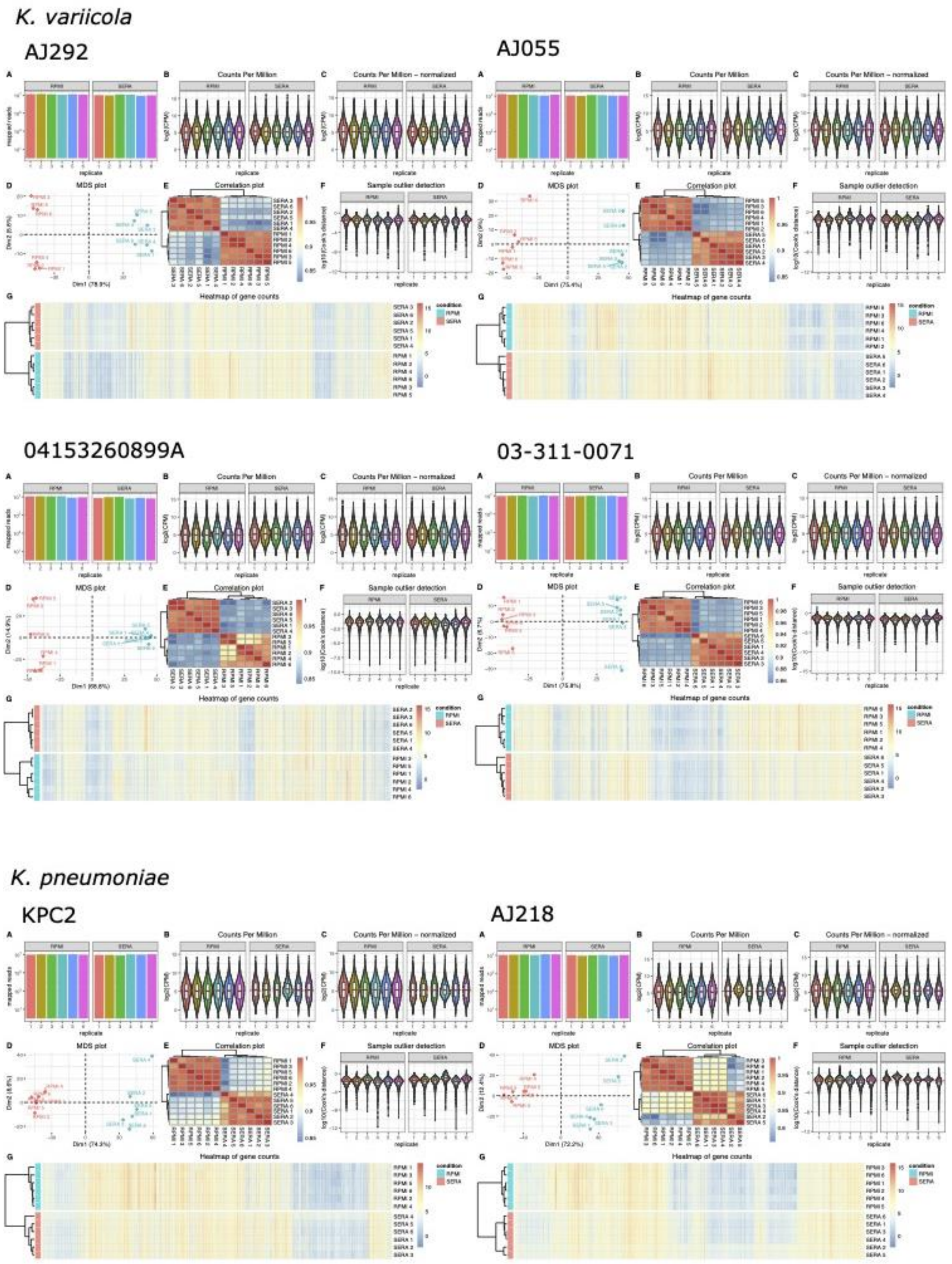
K. variicola
AJ292
AJ055
04153260899A
03-311-0071
K. pneumoniae
KPC2
AJ218
Counts Per Million
Counts Per Million – normalized
MDS plot
Correlation plot
Sample outlier detection
Heatmap of gene counts

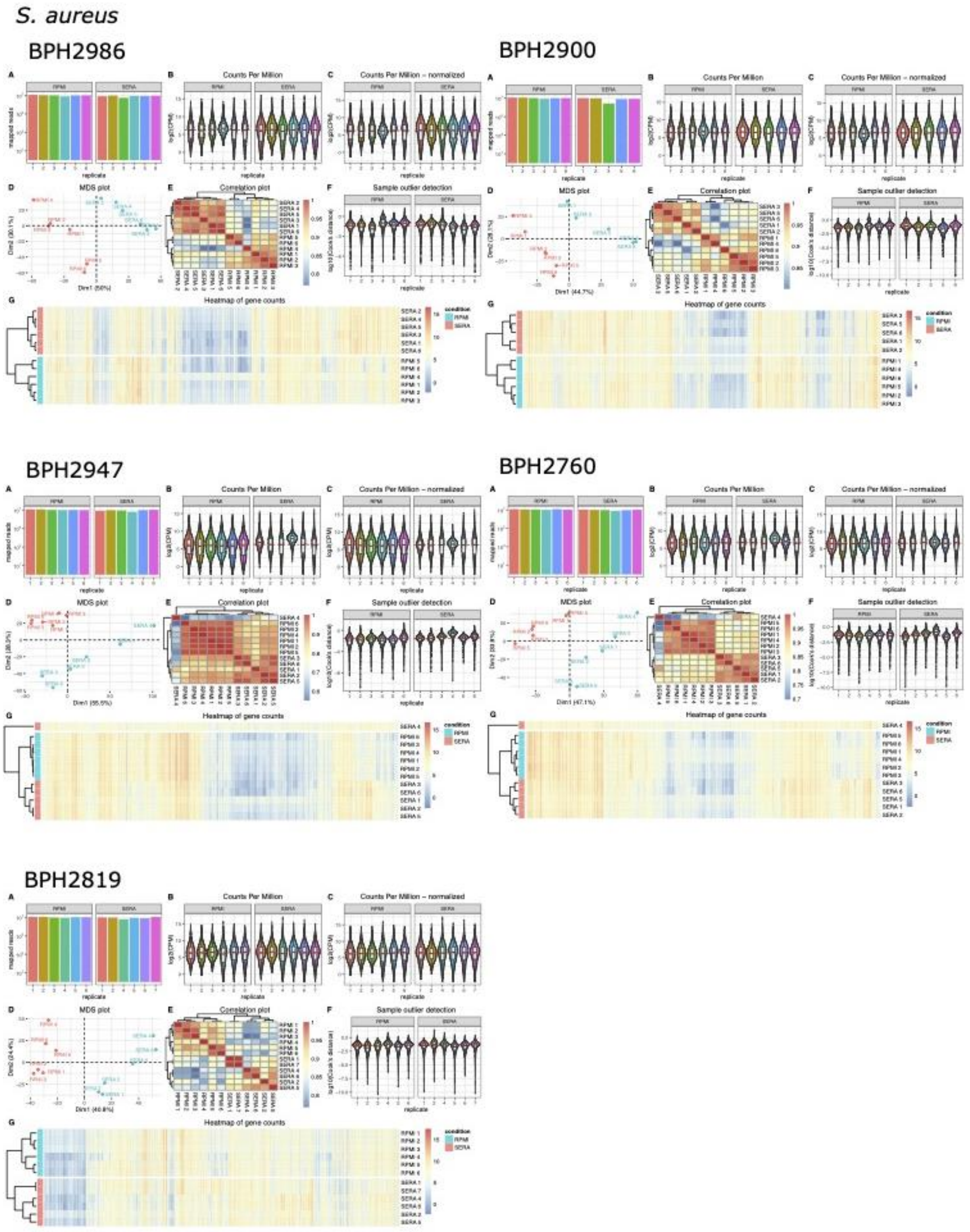
S. aureus
BPH2986
BPH2900
BPH2947
BPH2760
BPH2819
Counts Per Million
Counts Per Million – normalized
MDS plot
Correlation plot
Sample outlier detection
Heatmap of gene counts

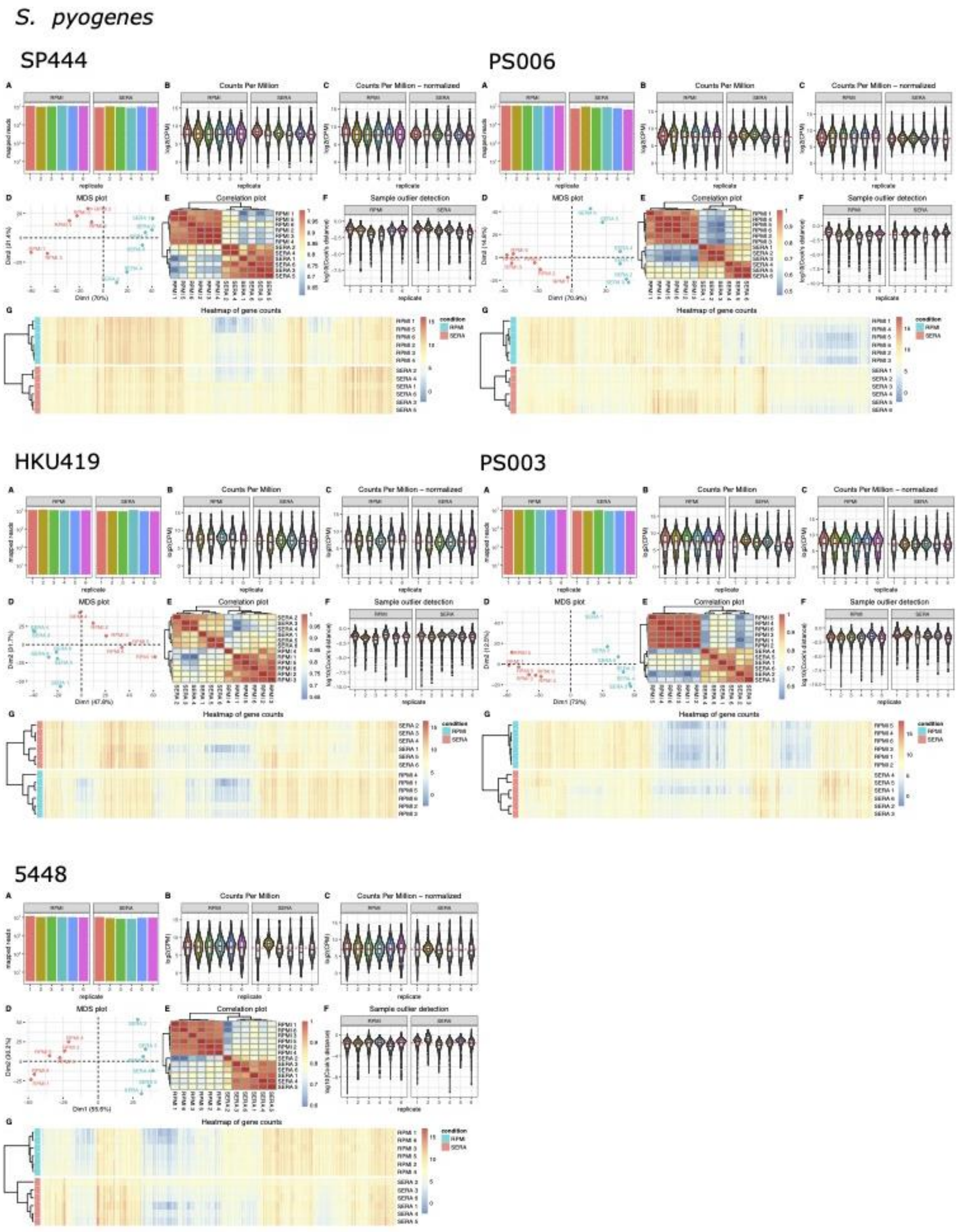

Supplementary Figure 1. Quality control analysis of RNAseq data for each strain included in this study. The following is reported across the six biological replicates per strain for each species when grown in RPMI and exposed to human sera: (A) the number of mapped reads; (B) read counts per million and (C) normalised read counts per million with boxplot markers indicating the median of the data, a box indicating the interquartile ranges, whiskers indicating the minimum and maximum values, and outliers highlighted by individual dots; (D) multi-

dimensional scaling plot visualising the separation in samples by growth in RPMI vs., exposure to human sera along the first dimension; (E) correlation plot of transcriptomic data; (F) detection of outlier samples with boxplot markers indicating the median of the data, a box indicating the interquartile ranges, whiskers indicating the minimum and maximum values, and outliers highlighted by individual dots; and (G) heatmap distribution of gene counts.

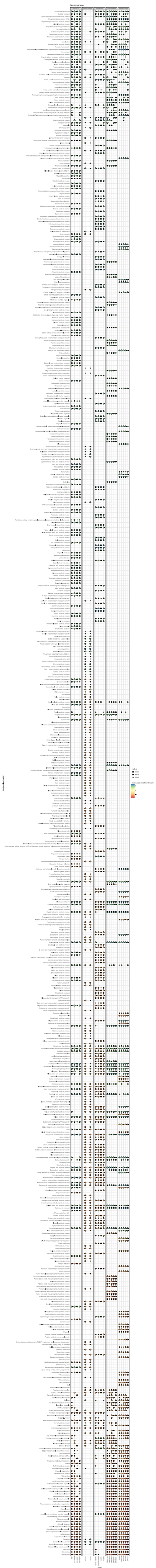

Supplementary Figure 2. Functional and metabolic pathway enrichment analysis to assess the shared transcriptome response to serum. Shapes and colours represent normalised enrichment scores and indicate up (blue) and down (red) regulated functions or pathways in serum. Only enriched Gene Ontology terms and KEGG metabolic pathways found to be significantly enriched in all strains of a species or 50% of all strains are represented.

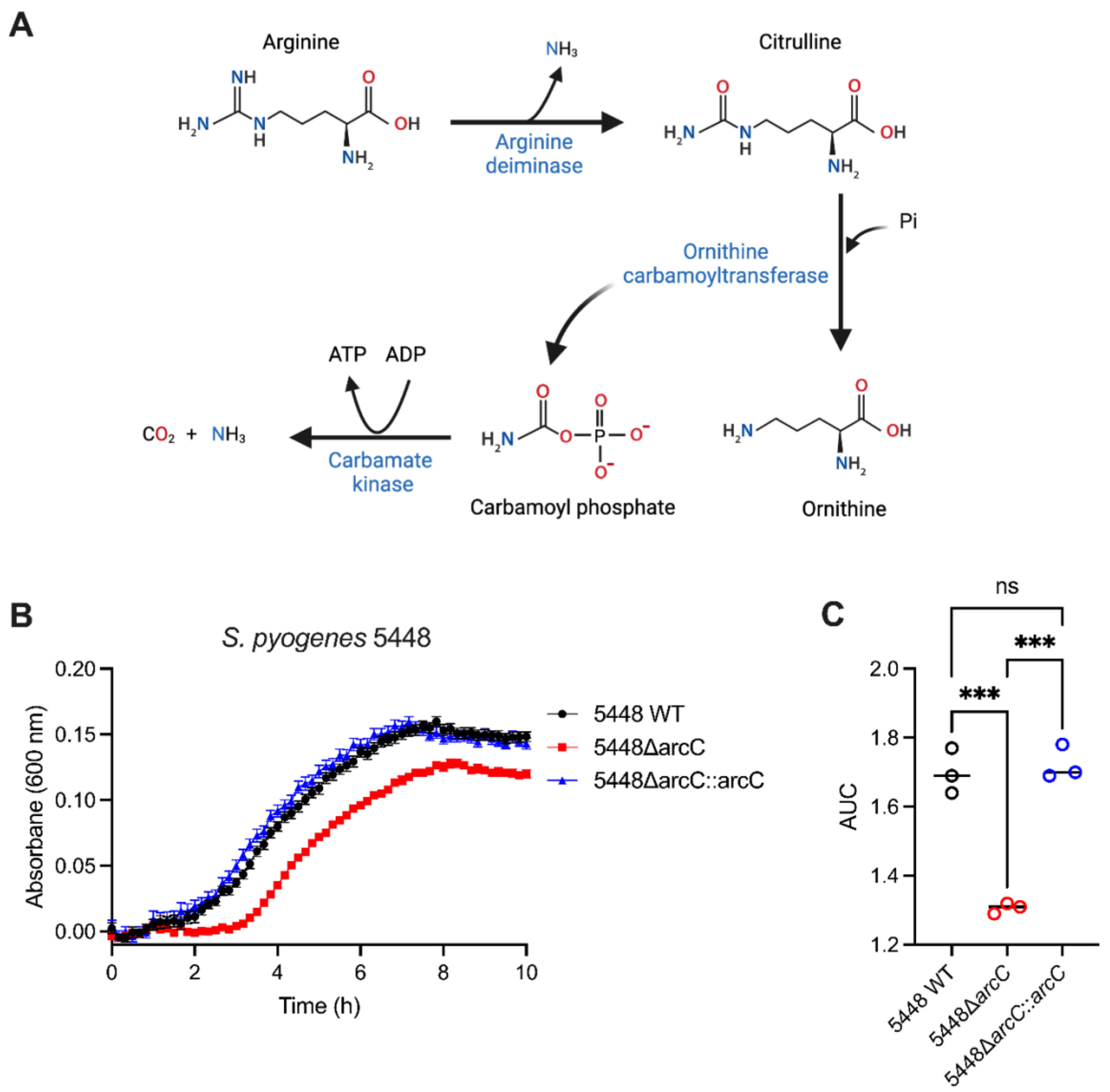

Supplementary Figure 3. Carbamate kinase augments growth of S. pyogenes in human serum. (A) The arginine deiminase pathway catalyses the conversion of arginine to ornithine and carbamoyl phosphate, enabling carbamate kinase mediated ATP, carbon dioxide and ammonia production. (B) Growth rates of GAS 5448 wild type, GAS 5448Δ*arcC*, and GAS 5448Δ*arcC* complemented with wild type *arcC* in human serum. The data corresponds with mean (± SEM) absorbance at 600 nm from three independent biological experiments undertaken in technical triplicate. (C) Area-under-the-curve (AUC) for each growth curve was calculated using the R package Growthcurver to compute AUC. Significance testing was

performed using Student's unpaired, two-sided t-test, with the null with the null hypothesis (no difference between mean AUC values) rejected for $p < 0.05$ (*** $p<0.001$) (n=3).

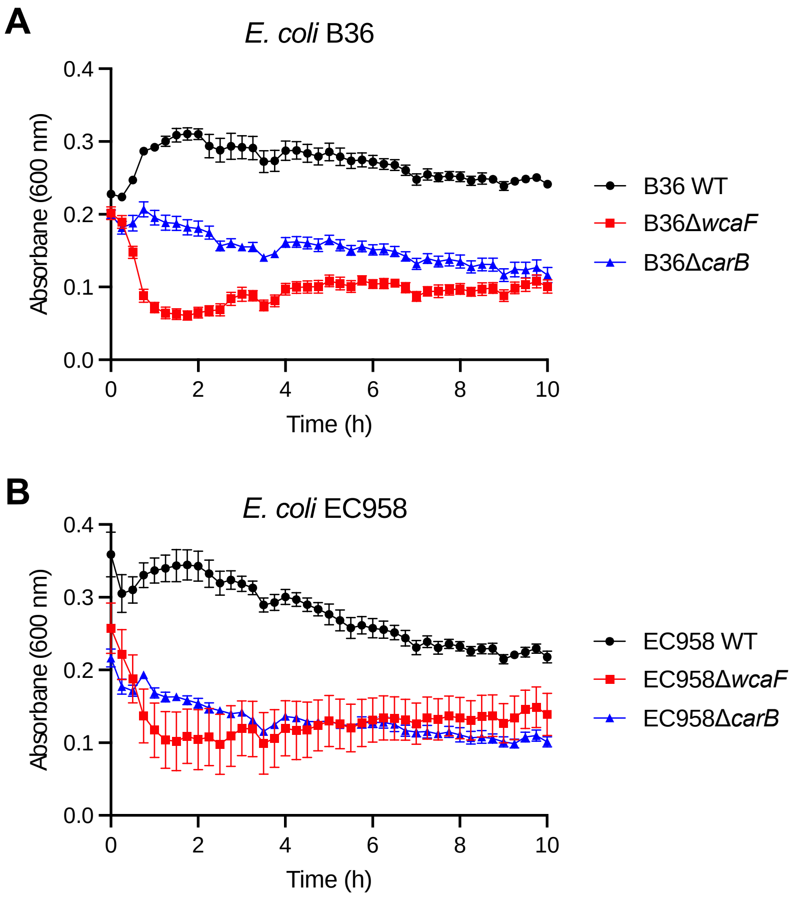

Supplementary Figure 4. wcaF and carB gene expression enhances E. coli survival in human serum. Survival of (A) *E. coli* B36, B36Δ*carB*, B36Δ*wcaF*, and (B) *E. coli* EC958, EC958Δ*carB*, EC958Δ*wcaF* in human serum. The data represents the mean (± SEM) absorbance at 600 nm from three independent biological experiments undertaken in technical triplicate.

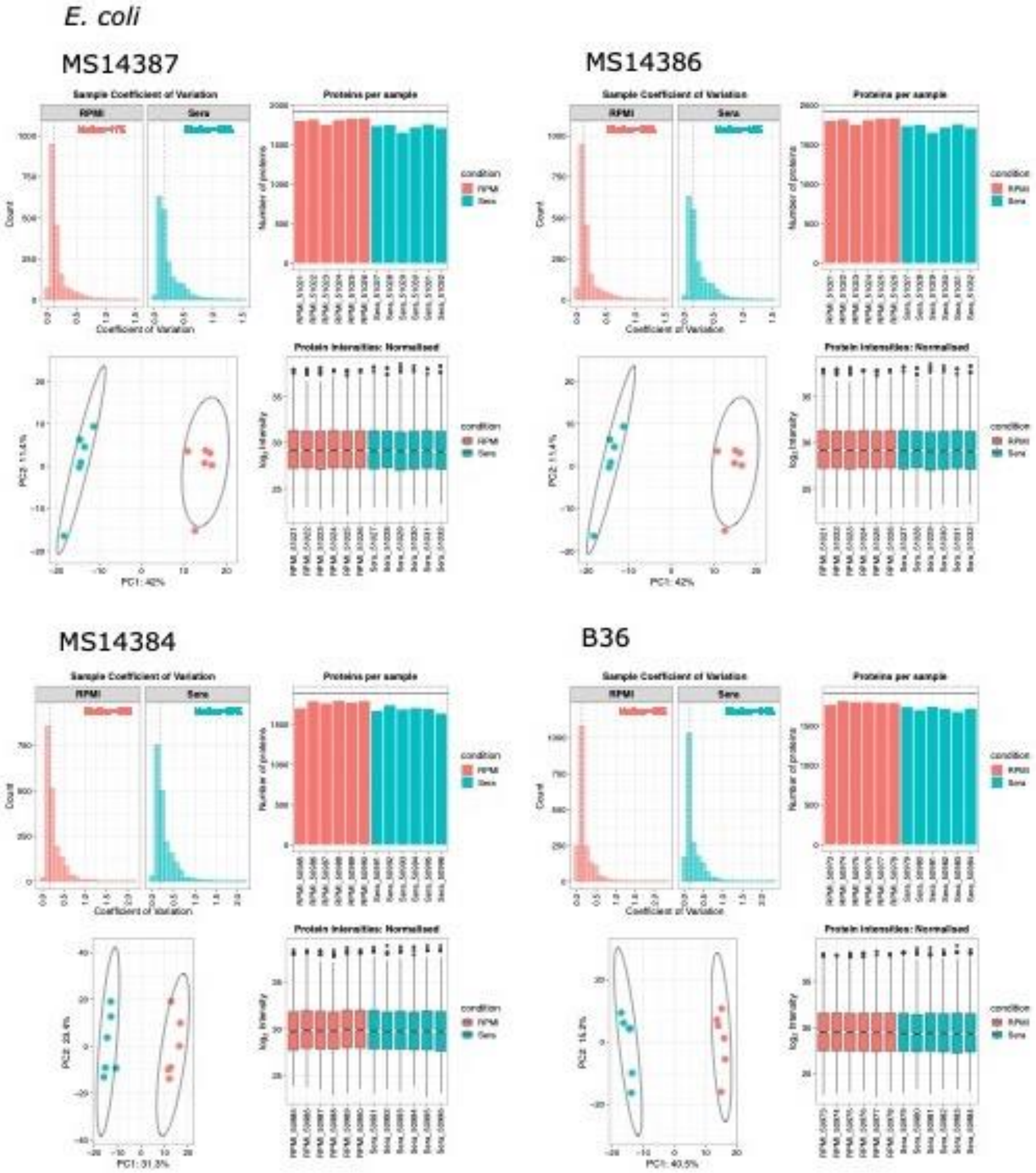

E. coli
MS14387
MS14386
MS14384
B36
Sample Coefficient of Variation
Proteins per sample
Protein intensities: Normalised
RPMI
Sera
condition
Count
Coefficient of Variation
Number of proteins
PC1: 42%
PC2: 11.4%
PC1: 31.3%
PC1: 40.5%

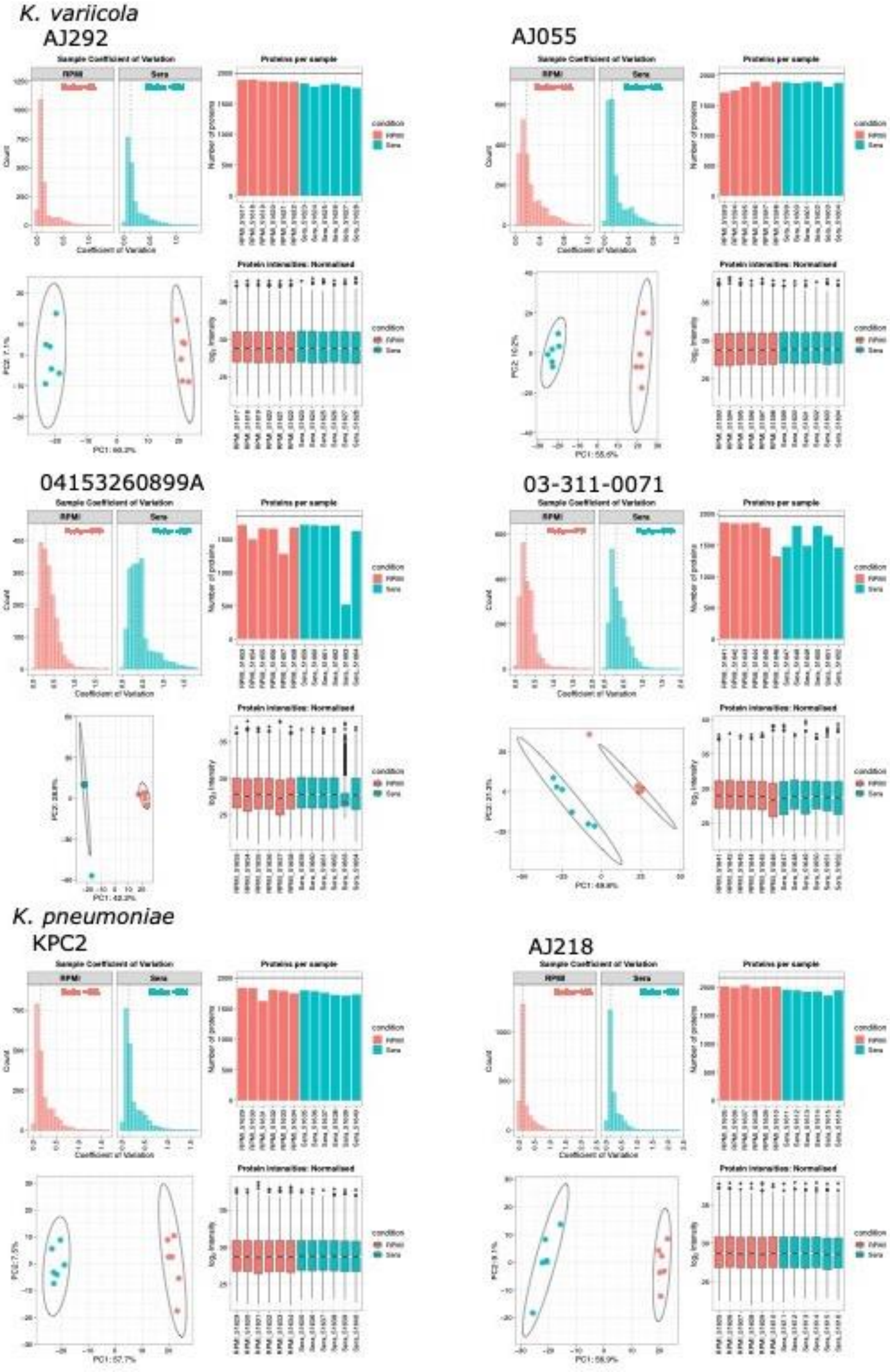
K. variicola
AJ292
AJ055
04153260899A
03-311-0071
K. pneumoniae
KPC2
AJ218
Sample Coefficient of Variation
Proteins per sample
Protein intensities: Normalised
RPMI
Sera
condition

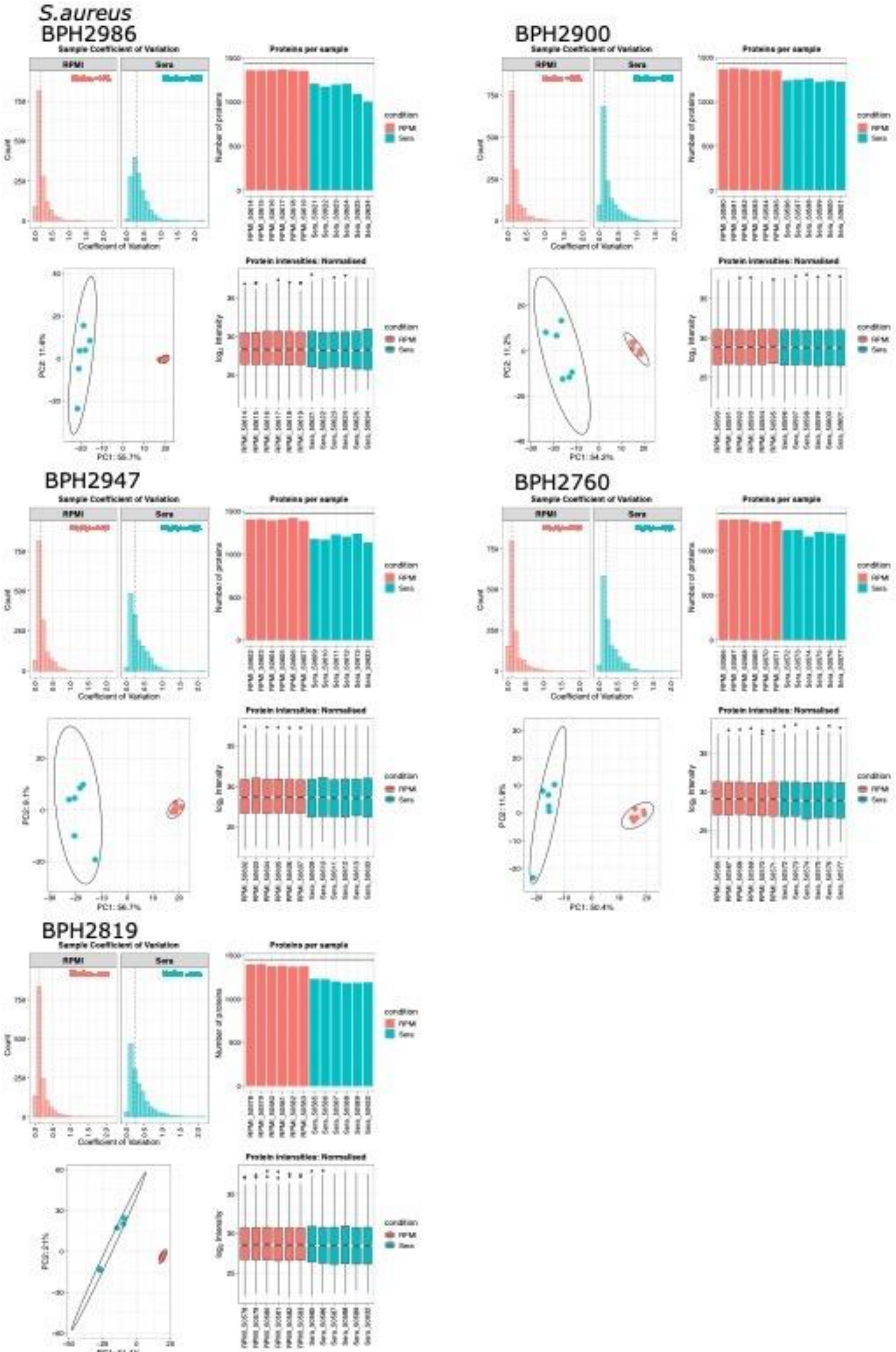
S.aureus
BPH2986
BPH2900
BPH2947
BPH2760
BPH2819
Sample Coefficient of Variation
Proteins per sample
Protein intensities: Normalised
RPMI
Sera
condition
Count
Coefficient of Variation
Number of proteins

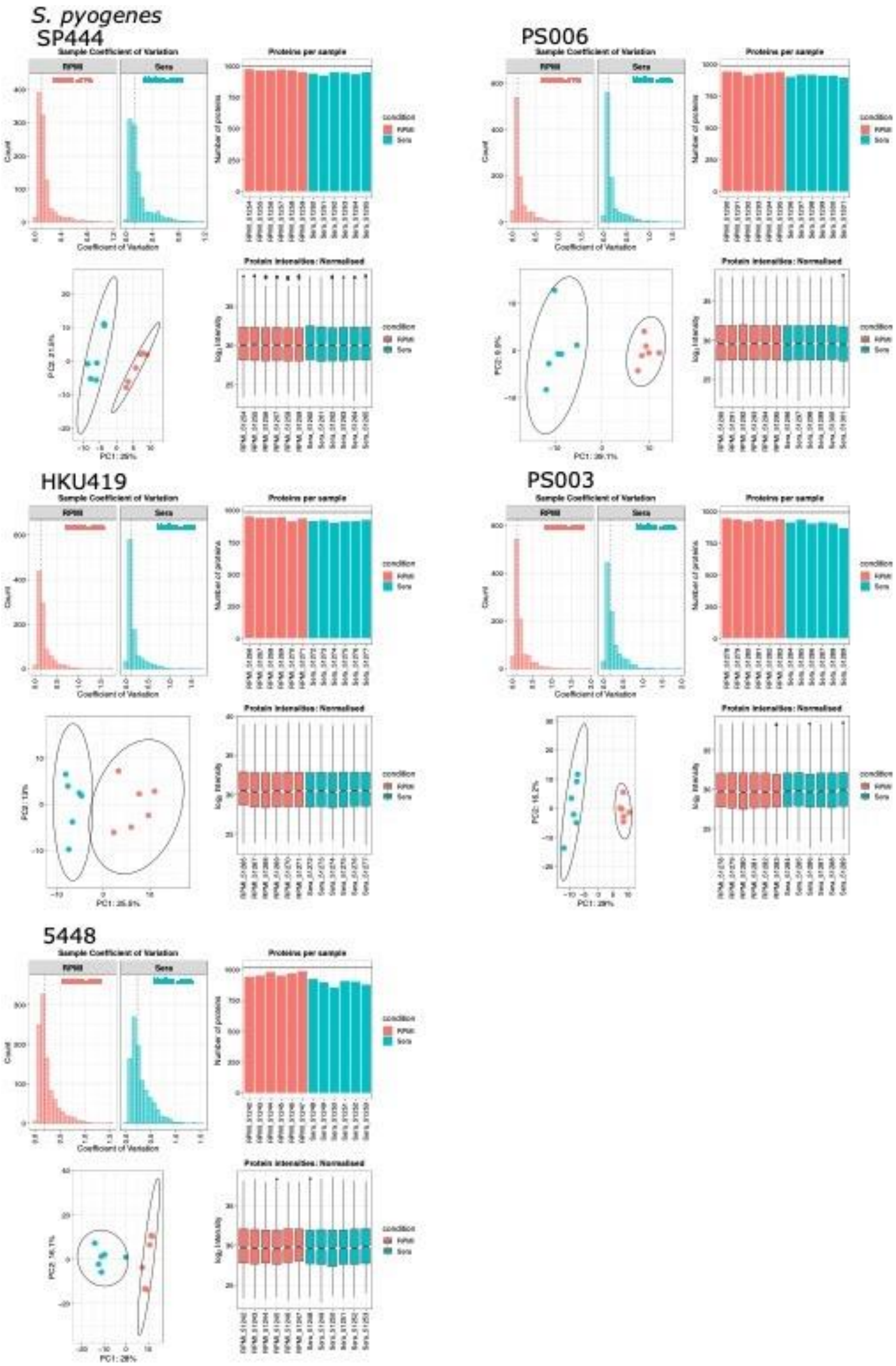

Supplementary Figure 5. **Quality control analysis of metabolomic data for each strain included in this study.** The following is reported across the six biological replicates per strain

for each species when grown in RPMI and exposed to human sera: sample coefficient of variation, number of proteins per sample, samples plotted on PCA sample space, and normalised protein intensities with boxplot markers indicating the median of the data, a box indicating the interquartile ranges, whiskers indicating the minimum and maximum values, and outliers highlighted by individual dots.

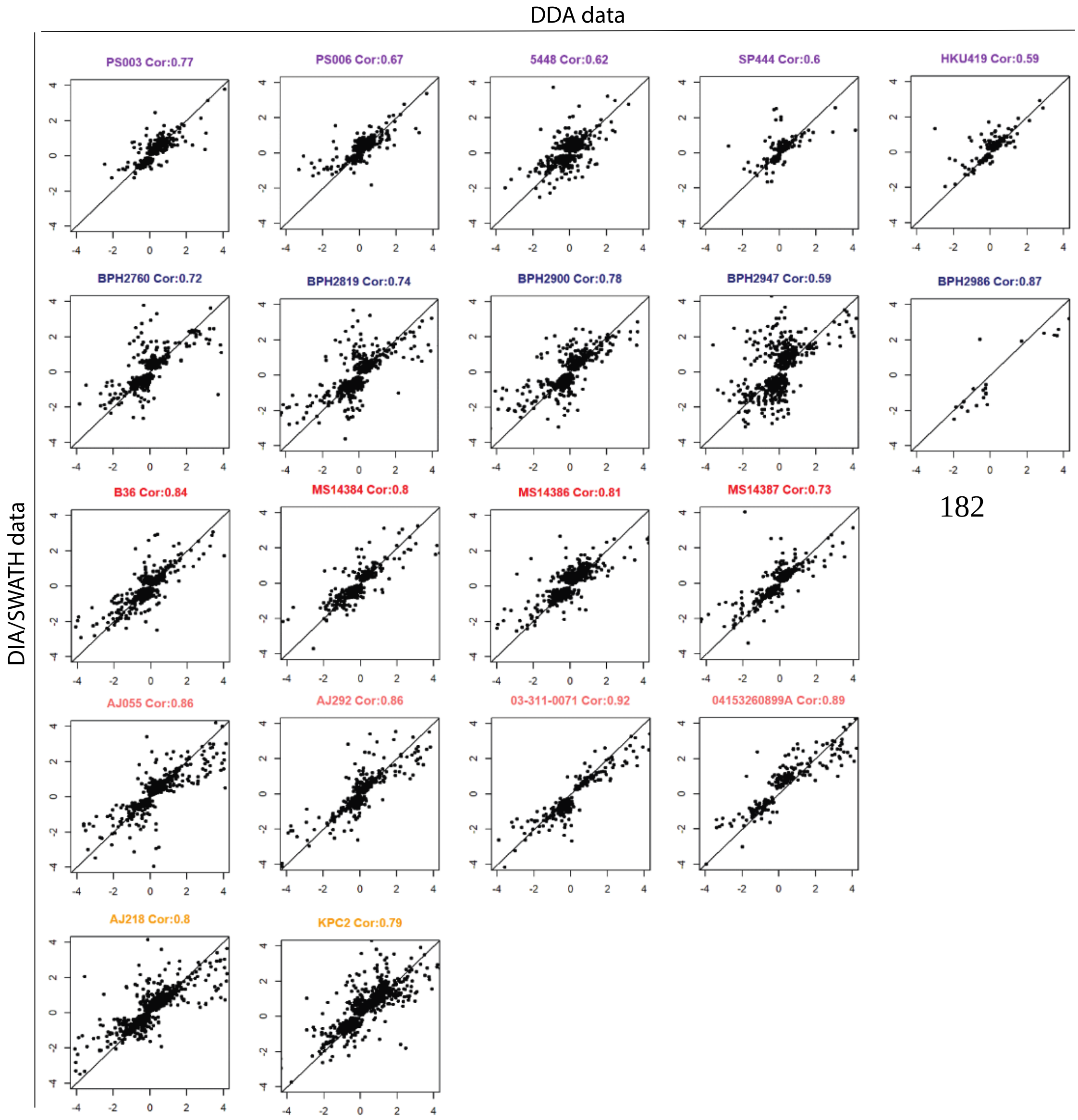

Supplementary Figure 6. Scatter plots showing the correlation between the DDA and DIA/SWATH proteomic datasets of the 20 pathogens. The log2 fold changes of the two datasets are plotted against each other with the DDA datasets shown on the x-axis and the DIA/SWATH datasets on the y-axis.

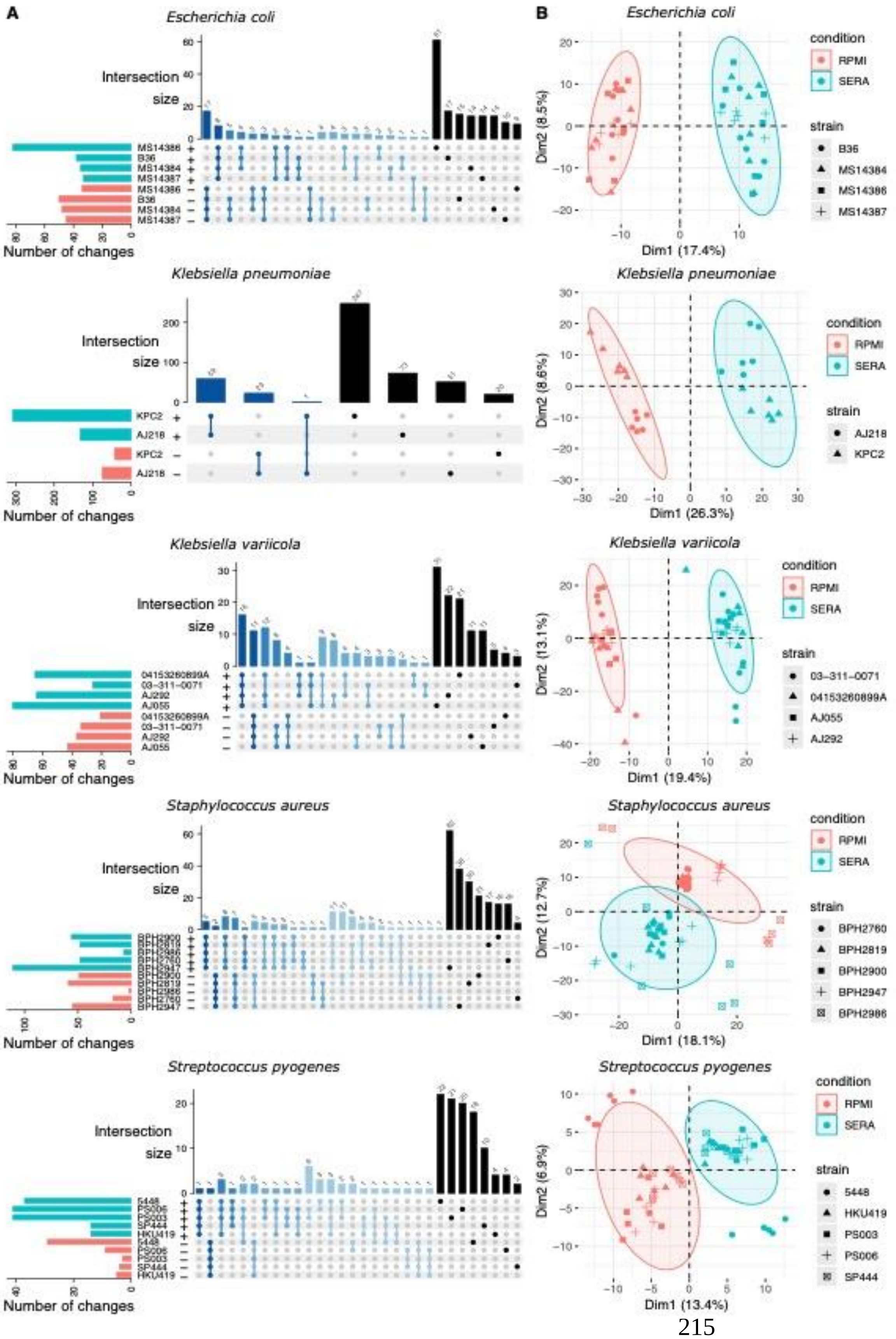
A
B
Escherichia coli
Intersection size
Number of changes
MS14386
B36
MS14384
MS14387
Dim1 (17.4%)
Dim2 (8.5%)
condition
RPMI
SERA
strain
Klebsiella pneumoniae
KPC2
AJ218
Dim1 (26.3%)
Dim2 (8.6%)
Klebsiella variicola
04153260899A
03-311-0071
AJ292
AJ055
Dim1 (19.4%)
Dim2 (13.1%)
Staphylococcus aureus
BPH2900
BPH2819
BPH2986
BPH2760
BPH2947
Dim1 (18.1%)
Dim2 (12.7%)
Streptococcus pyogenes
5448
PS006
PS003
SP444
HKU419
Dim1 (13.4%)
Dim2 (6.9%)

Supplementary Figure 7. Data-independent acquisition/sequential window acquisition of all theoretical mass spectra (DIA/SWATH) mass spectrometry to assess the impact of serum exposure on proteome within the different species. (A) UpSet plots representing the shared and distinctive proteome responses across strains of the same species. Only proteins with significant differential expression after exposure to human serum are represented (FDR<0.05; |log2 fold change|>1). (B) Multidimensional scaling plots of the core-proteins responses across strains of the same species demonstrating a clear separation of serum exposed samples for all species. See Fig. 4 legend for more detailed explanation of the figures.

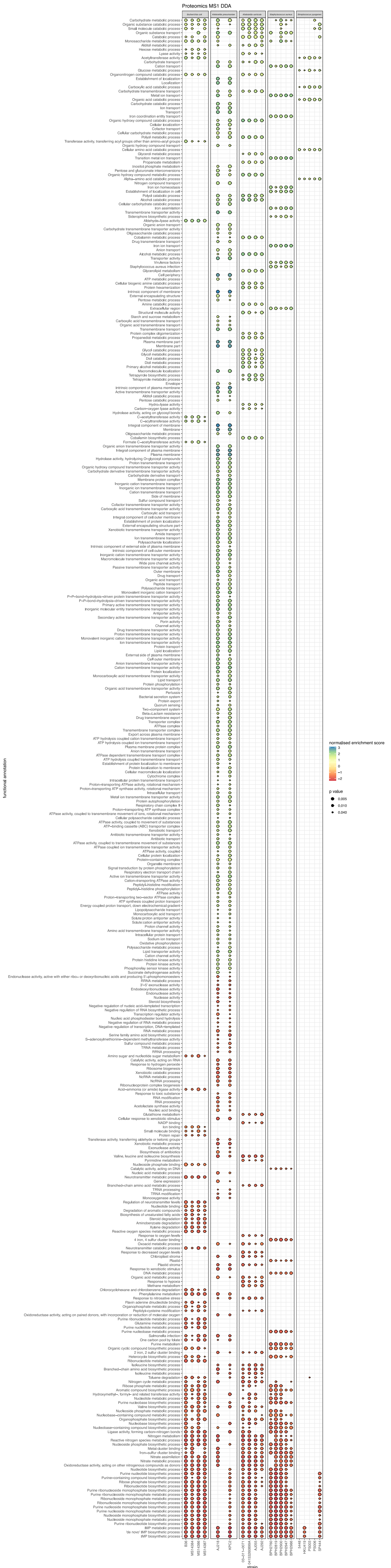
Proteomics MS1 DDA
functional annotation
strain
normalised enrichment score
p value

Supplementary Figure 8. Functional and metabolic pathway enrichment analysis to assess the shared proteome response to serum (DDA mass spectrometry). Shapes and colours represent normalised enrichment scores and indicate up (blue) and down (red) regulated functions or pathways in serum (two-sided Fisher's exact test). Only enriched Gene Ontology terms and KEGG metabolic pathways found to be significantly enriched in all strains of a species or 50% of all strains are represented.

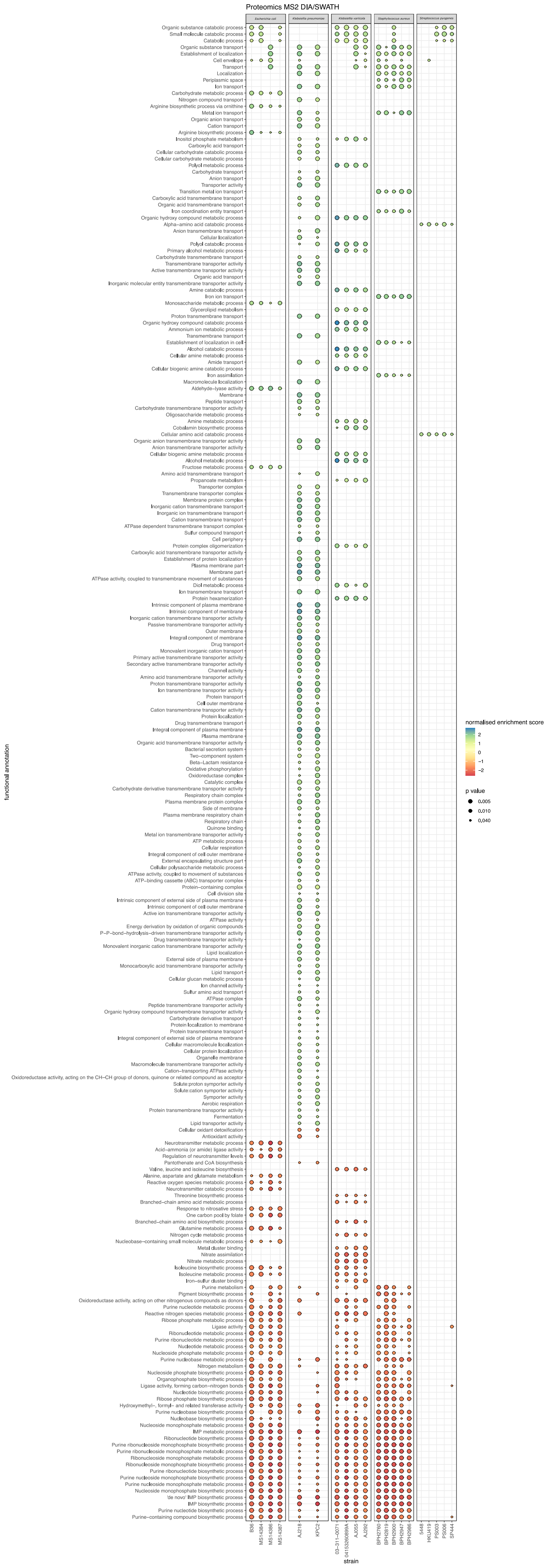

Proteomics MS2 DIA/SWATH
Escherichia coli
Klebsiella pneumoniae
Klebsiella variicola
Staphylococcus aureus
Streptococcus pyogenes
functional annotation
Organic substance catabolic process
Small molecule catabolic process
Catabolic process
Organic substance transport
Establishment of localization
Cell envelope
Transport
Localization
Periplasmic space
Ion transport
Carbohydrate metabolic process
Nitrogen compound transport
Arginine biosynthetic process via ornithine
Metal ion transport
Organic anion transport
Cation transport
Arginine biosynthetic process
Inositol phosphate metabolism
Carboxylic acid transport
Cellular carbohydrate catabolic process
Cellular carbohydrate metabolic process
Polyol metabolic process
Carbohydrate transport
Anion transport
Transporter activity
Transition metal ion transport
Carboxylic acid transmembrane transport
Organic acid transmembrane transport
Iron coordination entity transport
Organic hydroxy compound metabolic process
Alpha−amino acid catabolic process
Anion transmembrane transport
Cellular localization
Polyol catabolic process
Primary alcohol metabolic process
Carbohydrate transmembrane transport
Transmembrane transporter activity
Active transmembrane transporter activity
Organic acid transport
Inorganic molecular entity transmembrane transporter activity
Amine catabolic process
Iron ion transport
Monosaccharide metabolic process
Glycerolipid metabolism
Proton transmembrane transport
Organic hydroxy compound catabolic process
Ammonium ion metabolic process
Transmembrane transport
Establishment of localization in cell
Alcohol catabolic process
Cellular amine metabolic process
Amide transport
Cellular biogenic amine catabolic process
Iron assimilation
Macromolecule localization
Aldehyde−lyase activity
Membrane
Peptide transport
Carbohydrate transmembrane transporter activity
Oligosaccharide metabolic process
Amine metabolic process
Cobalamin biosynthetic process
Cellular amino acid catabolic process
Organic anion transmembrane transporter activity
Anion transmembrane transporter activity
Cellular biogenic amine metabolic process
Alcohol metabolic process
Fructose metabolic process
Amino acid transmembrane transport
Propanoate metabolism
Transporter complex
Transmembrane transporter complex
Membrane protein complex
Inorganic cation transmembrane transport
Inorganic ion transmembrane transport
Cation transmembrane transport
ATPase dependent transmembrane transport complex
Sulfur compound transport
Cell periphery
Protein complex oligomerization
Carboxylic acid transmembrane transporter activity
Establishment of protein localization
Plasma membrane part
Membrane part
ATPase activity, coupled to transmembrane movement of substances
Diol metabolic process
Ion transmembrane transport
Protein hexamerization
Intrinsic component of plasma membrane
Intrinsic component of membrane
Inorganic cation transmembrane transporter activity
Passive transmembrane transporter activity
Outer membrane
Integral component of membrane
Drug transport
Monovalent inorganic cation transport
Primary active transmembrane transporter activity
Secondary active transmembrane transporter activity
Channel activity
Amino acid transmembrane transporter activity
Proton transmembrane transporter activity
Ion transmembrane transporter activity
Protein transport
Cell outer membrane
Cation transmembrane transporter activity
Protein localization
Drug transmembrane transport
Integral component of plasma membrane
Plasma membrane
Organic acid transmembrane transporter activity
Bacterial secretion system
Two−component system
Beta−Lactam resistance
Oxidative phosphorylation
Oxidoreductase complex
Catalytic complex
Carbohydrate derivative transmembrane transporter activity
Respiratory chain complex
Plasma membrane protein complex
Side of membrane
Plasma membrane respiratory chain
Respiratory chain
Quinone binding
Metal ion transmembrane transporter activity
ATP metabolic process
Cellular respiration
Integral component of cell outer membrane
External encapsulating structure part
Cellular polysaccharide metabolic process
ATPase activity, coupled to movement of substances
ATP−binding cassette (ABC) transporter complex
Protein−containing complex
Cell division site
Intrinsic component of external side of plasma membrane
Intrinsic component of cell outer membrane
Active ion transmembrane transporter activity
ATPase activity
Energy derivation by oxidation of organic compounds
P−P−bond−hydrolysis−driven transmembrane transporter activity
Drug transmembrane transporter activity
Monovalent inorganic cation transmembrane transporter activity
Lipid localization
External side of plasma membrane
Monocarboxylic acid transmembrane transporter activity
Lipid transport
Cellular glucan metabolic process
Ion channel activity
Sulfur amino acid transport
ATPase complex
Peptide transmembrane transporter activity
Organic hydroxy compound transmembrane transporter activity
Carbohydrate derivative transport
Protein localization to membrane
Protein transmembrane transport
Integral component of external side of plasma membrane
Cellular macromolecule localization
Cellular protein localization
Organelle membrane
Macromolecule transmembrane transporter activity
Cation−transporting ATPase activity
Oxidoreductase activity, acting on the CH−CH group of donors, quinone or related compound as acceptor
Solute proton symporter activity
Solute cation symporter activity
Symporter activity
Aerobic respiration
Protein transmembrane transporter activity
Fermentation
Lipid transporter activity
Cellular oxidant detoxification
Antioxidant activity
Neurotransmitter metabolic process
Acid−ammonia (or amide) ligase activity
Regulation of neurotransmitter levels
Pantothenate and CoA biosynthesis
Valine, leucine and isoleucine biosynthesis
Alanine, aspartate and glutamate metabolism
Reactive oxygen species metabolic process
Neurotransmitter catabolic process
Threonine biosynthetic process
Branched−chain amino acid metabolic process
Response to nitrosative stress
One carbon pool by folate
Branched−chain amino acid biosynthetic process
Glutamine metabolic process
Nitrogen cycle metabolic process
Nucleobase−containing small molecule metabolic process
Metal cluster binding
Nitrate assimilation
Nitrate metabolic process
Isoleucine biosynthetic process
Isoleucine metabolic process
Iron−sulfur cluster binding
Purine metabolism
Pigment biosynthetic process
Oxidoreductase activity, acting on other nitrogenous compounds as donors
Purine nucleotide metabolic process
Reactive nitrogen species metabolic process
Ribose phosphate metabolic process
Ligase activity
Ribonucleotide metabolic process
Purine ribonucleotide metabolic process
Nucleotide metabolic process
Nucleoside phosphate metabolic process
Purine nucleobase metabolic process
Nitrogen metabolism
Nucleoside phosphate biosynthetic process
Organophosphate biosynthetic process
Ligase activity, forming carbon−nitrogen bonds
Nucleotide biosynthetic process
Ribose phosphate biosynthetic process
Hydroxymethyl−, formyl− and related transferase activity
Purine nucleobase biosynthetic process
Nucleobase biosynthetic process
Nucleoside monophosphate metabolic process
IMP metabolic process
Ribonucleotide biosynthetic process
Purine ribonucleoside monophosphate biosynthetic process
Purine ribonucleoside monophosphate metabolic process
Ribonucleoside monophosphate metabolic process
Ribonucleoside monophosphate biosynthetic process
Purine ribonucleotide biosynthetic process
Purine nucleoside monophosphate biosynthetic process
Purine nucleoside monophosphate metabolic process
Nucleoside monophosphate biosynthetic process
'de novo' IMP biosynthetic process
IMP biosynthetic process
Purine nucleotide biosynthetic process
Purine−containing compound biosynthetic process
B36
MS14384
MS14386
MS14387
AJ218
KPC2
03−311−0071
04153260899A
AJ055
AJ292
BPH2760
BPH2819
BPH2900
BPH2947
BPH2986
5448
HKU419
PS003
PS006
SP444
strain
normalised enrichment score
2
1
0
−1
−2
p value
0,005
0,010
0,040

Supplementary Figure 9. Functional and metabolic pathway enrichment analysis to assess the shared proteome response to serum (DIA/SWATH mass spectrometry). Shapes and colours represent normalised enrichment scores and indicate up (blue) and down (red) regulated functions or pathways in serum (two-sided Fisher's exact test). Only enriched Gene Ontology terms and KEGG metabolic pathways found to be significantly enriched in all strains of a species or 50% of all strains are represented.

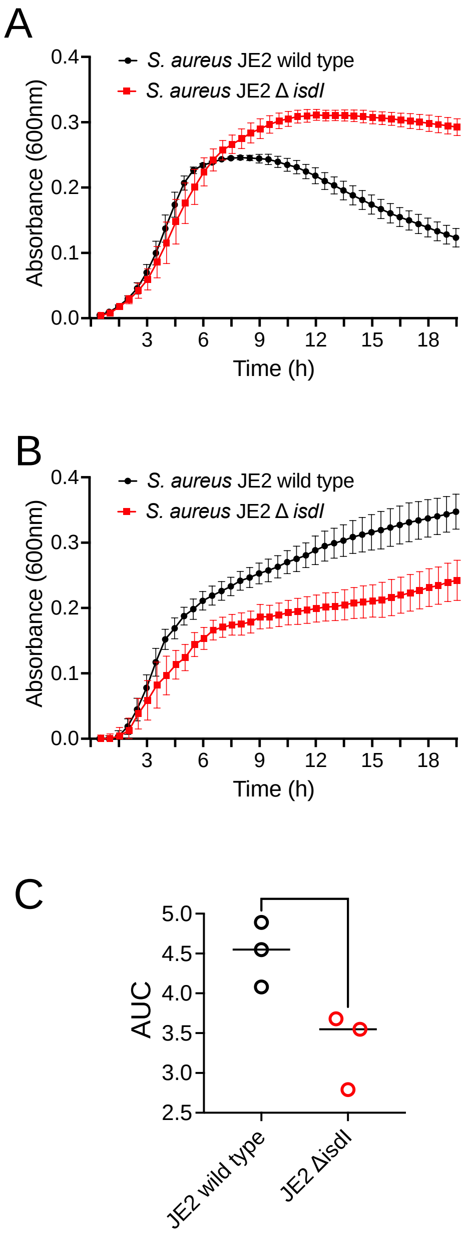

Supplementary Figure 10. Growth kinetics of S. aureus in 50% human serum using S. aureus strain JE2 wild type compared to the JE2 ΔisdI transposon mutant. (A) Growth in RPMI only. (B) Growth in RPMI with 50% heat-treated human serum (v/v). The data in (A) and (B) represents the mean (± SEM) absorbance at 600 nm from three independent biological experiments. (C) Comparison of mean area-under-the-curve (AUC) values for each of the three

biological replicates depicted in (B) showing a significant difference between mutant and wild type (two-sided Student’s unpaired t-test * $p=0.03$).

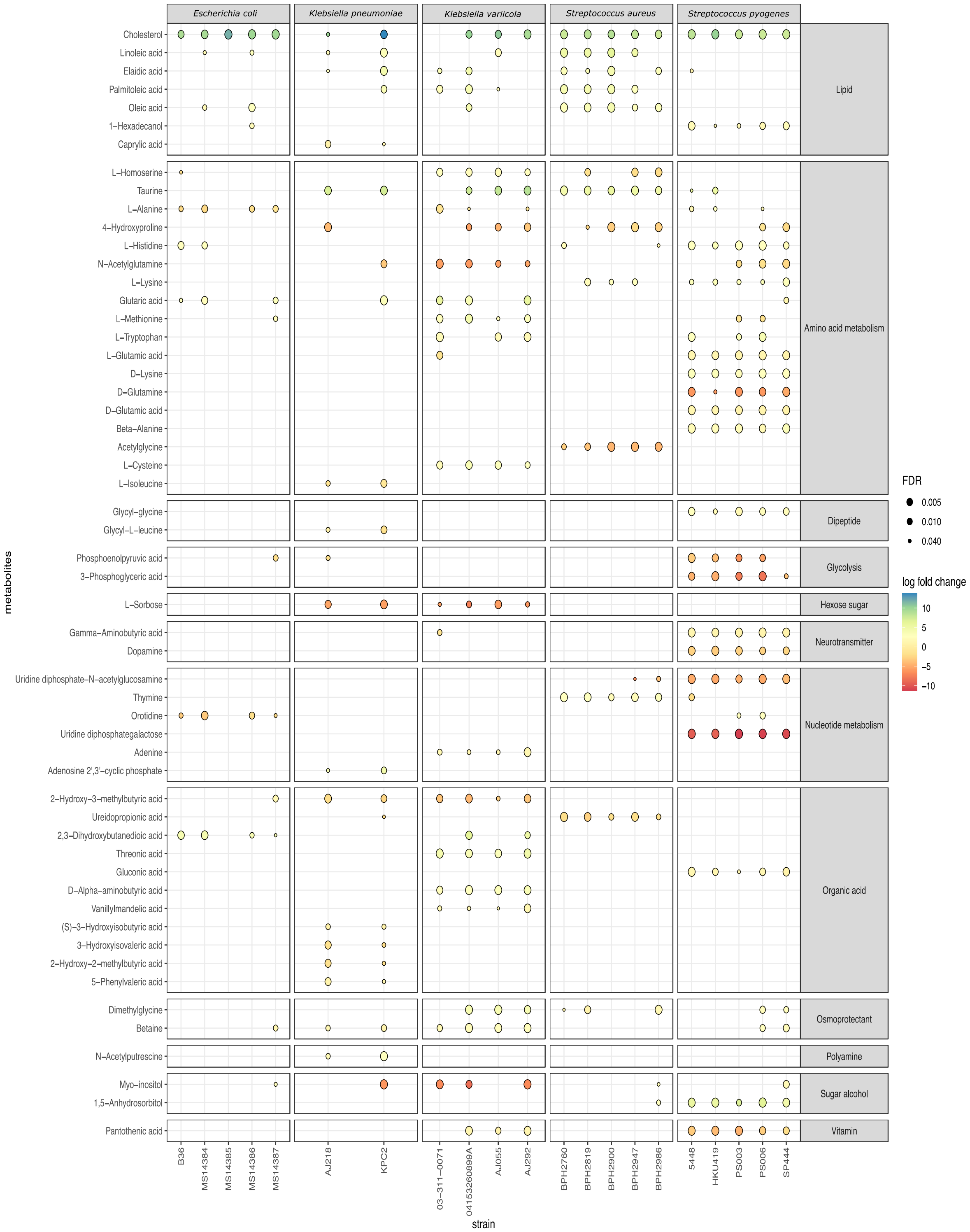

Escherichia coli
Klebsiella pneumoniae
Klebsiella variicola
Streptococcus aureus
Streptococcus pyogenes
Cholesterol
Linoleic acid
Elaidic acid
Palmitoleic acid
Oleic acid
1−Hexadecanol
Caprylic acid
Lipid
L−Homoserine
Taurine
L−Alanine
4−Hydroxyproline
L−Histidine
N−Acetylglutamine
L−Lysine
Glutaric acid
L−Methionine
L−Tryptophan
L−Glutamic acid
D−Lysine
D−Glutamine
D−Glutamic acid
Beta−Alanine
Acetylglycine
L−Cysteine
L−Isoleucine
Amino acid metabolism
Glycyl−glycine
Glycyl−L−leucine
Dipeptide
Phosphoenolpyruvic acid
3−Phosphoglyceric acid
Glycolysis
L−Sorbose
Hexose sugar
Gamma−Aminobutyric acid
Dopamine
Neurotransmitter
Uridine diphosphate−N−acetylglucosamine
Thymine
Orotidine
Uridine diphosphategalactose
Adenine
Adenosine 2',3'−cyclic phosphate
Nucleotide metabolism
2−Hydroxy−3−methylbutyric acid
Ureidopropionic acid
2,3−Dihydroxybutanedioic acid
Threonic acid
Gluconic acid
D−Alpha−aminobutyric acid
Vanillylmandelic acid
(S)−3−Hydroxyisobutyric acid
3−Hydroxyisovaleric acid
2−Hydroxy−2−methylbutyric acid
5−Phenylvaleric acid
Organic acid
Dimethylglycine
Betaine
Osmoprotectant
N−Acetylputrescine
Polyamine
Myo−inositol
1,5−Anhydrosorbitol
Sugar alcohol
Pantothenic acid
Vitamin
metabolites
B36
MS14384
MS14385
MS14386
MS14387
AJ218
KPC2
03−311−0071
04153260899A
AJ055
AJ292
BPH2760
BPH2819
BPH2900
BPH2947
BPH2986
5448
HKU419
PS003
PS006
SP444
strain
FDR
0.005
0.010
0.040
log fold change
10
5
0
−5
−10

Supplementary Figure 11. Functional and metabolic pathway enrichment analysis to assess the shared metabolome response to serum (GC-MS). Shapes and colours represent normalised enrichment scores and indicate up (blue) and down (red) regulated functions or pathways in serum. Only enriched Gene Ontology terms and KEGG metabolic pathways found to be significantly enriched in all strains of a species or 50% of all strains are represented.

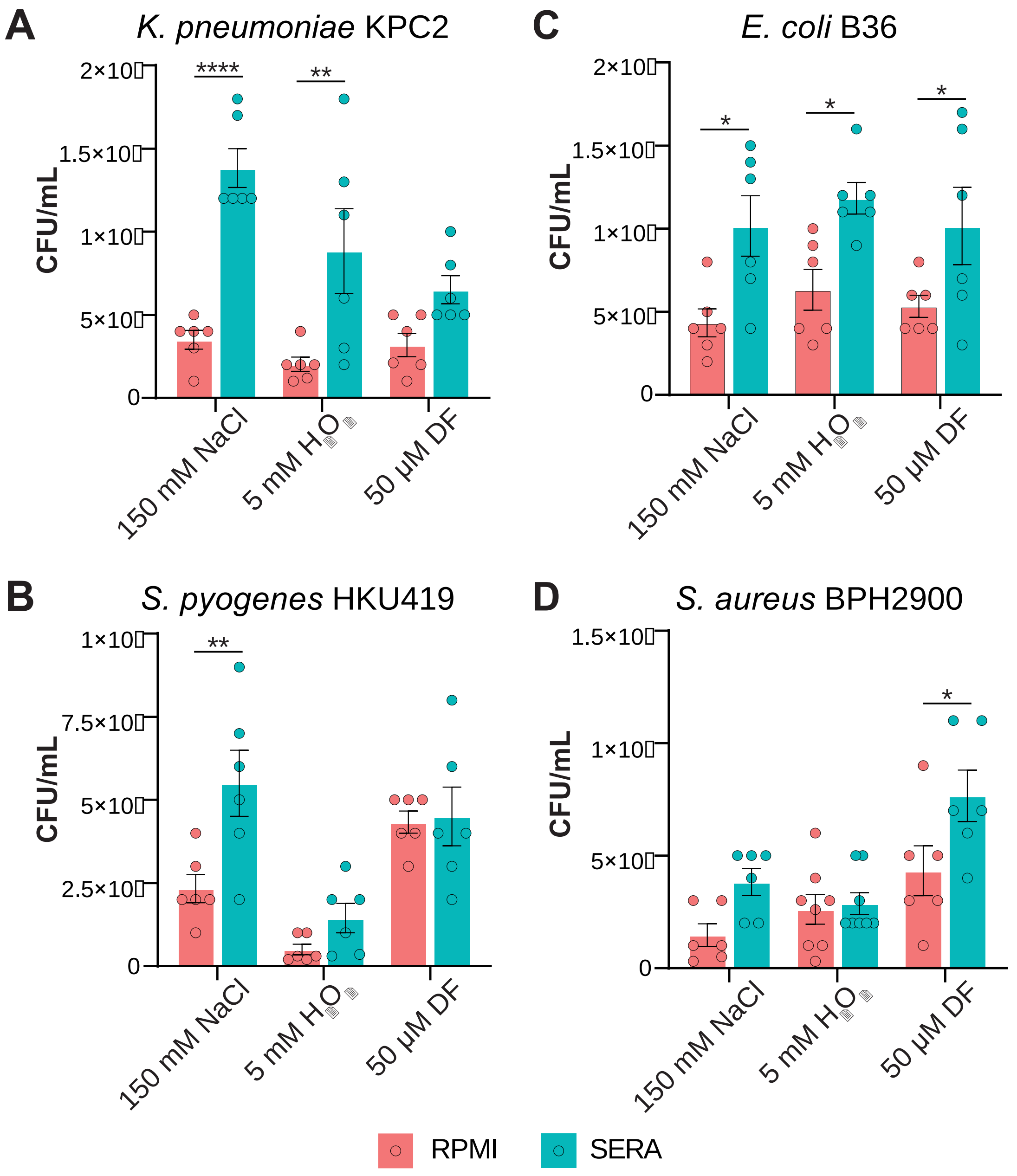

Supplementary Figure 12. Stress survival assays following exposure to serum. (A) *K. pneumoniae* KPC2; (B) *S. pyogenes* HKU419; (C) *E. coli* B36; (D) *S. aureus* BPH2900 were incubated in RPMI or serum (5 mL each) for 2 hrs. Cells were collected and then exposed to either (each graph, left) 150 mM NaCl (osmotic stress), (middle) 5 mM H_2O_2 (oxidative stress), or (right) 50 µM deferoxamine (DF; iron limitation stress) for 1 hr at 37°C, and survival

determined by enumeration of CFU. Error bars indicate the mean standard error from 6 biological replicates for all strains and conditions test – except for *S. aureus* BPH2900 tested at 5 mM H_2O_2 in sera and RPMI which had 8 biological replicates – and statistical significance was determined using two-way ANOVA, * p <0.05; ** p <0.01, ****p <0.0001; Holm-*Šídák test.*

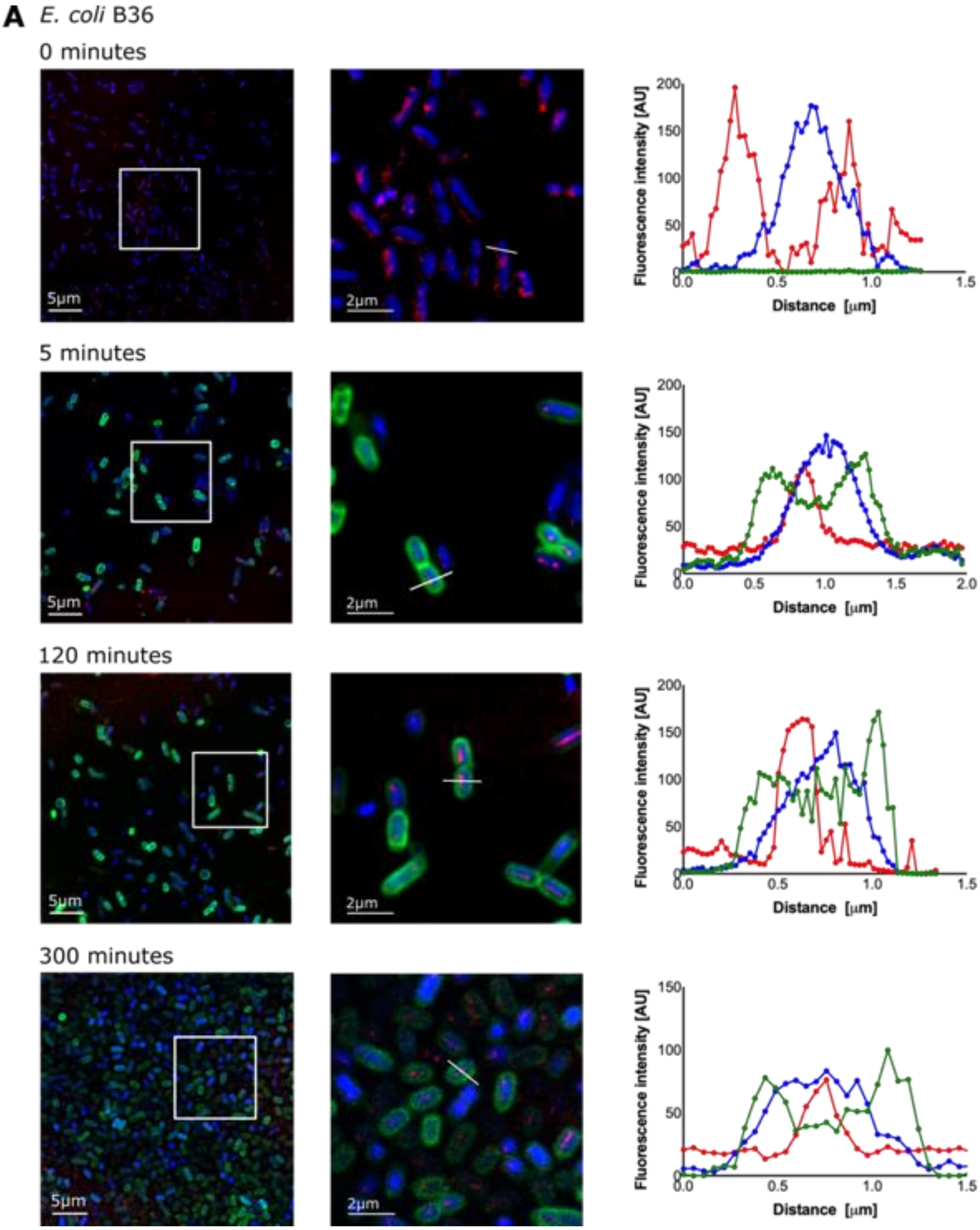
A E. coli B36
0 minutes
5 minutes
120 minutes
300 minutes
5µm
2µm
Fluorescence intensity [AU]
Distance [µm]

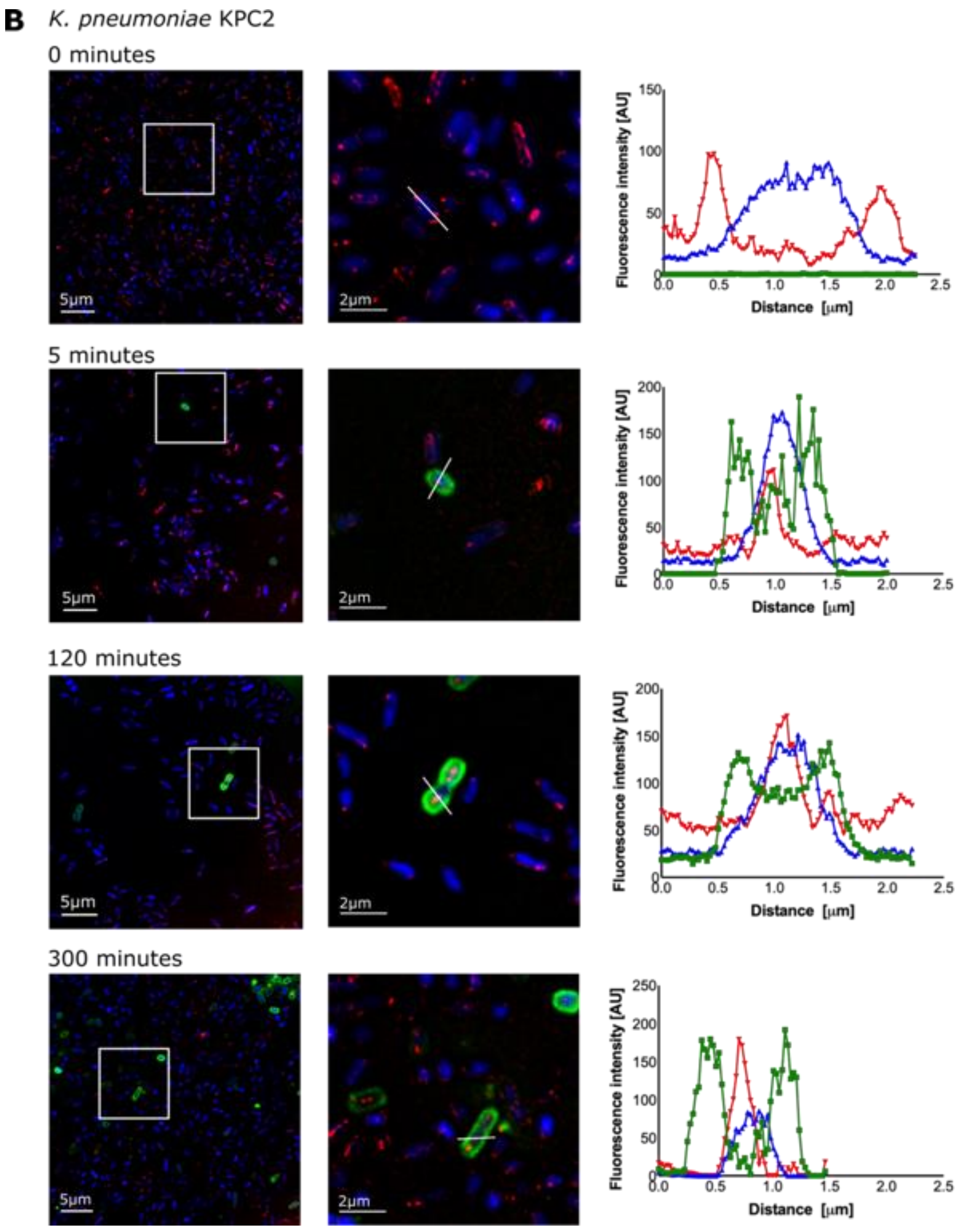
B
K. pneumoniae KPC2
0 minutes
5μm
2μm
Fluorescence intensity [AU]
Distance [μm]
5 minutes
120 minutes
300 minutes

318

C *S. aureus* BPH2900

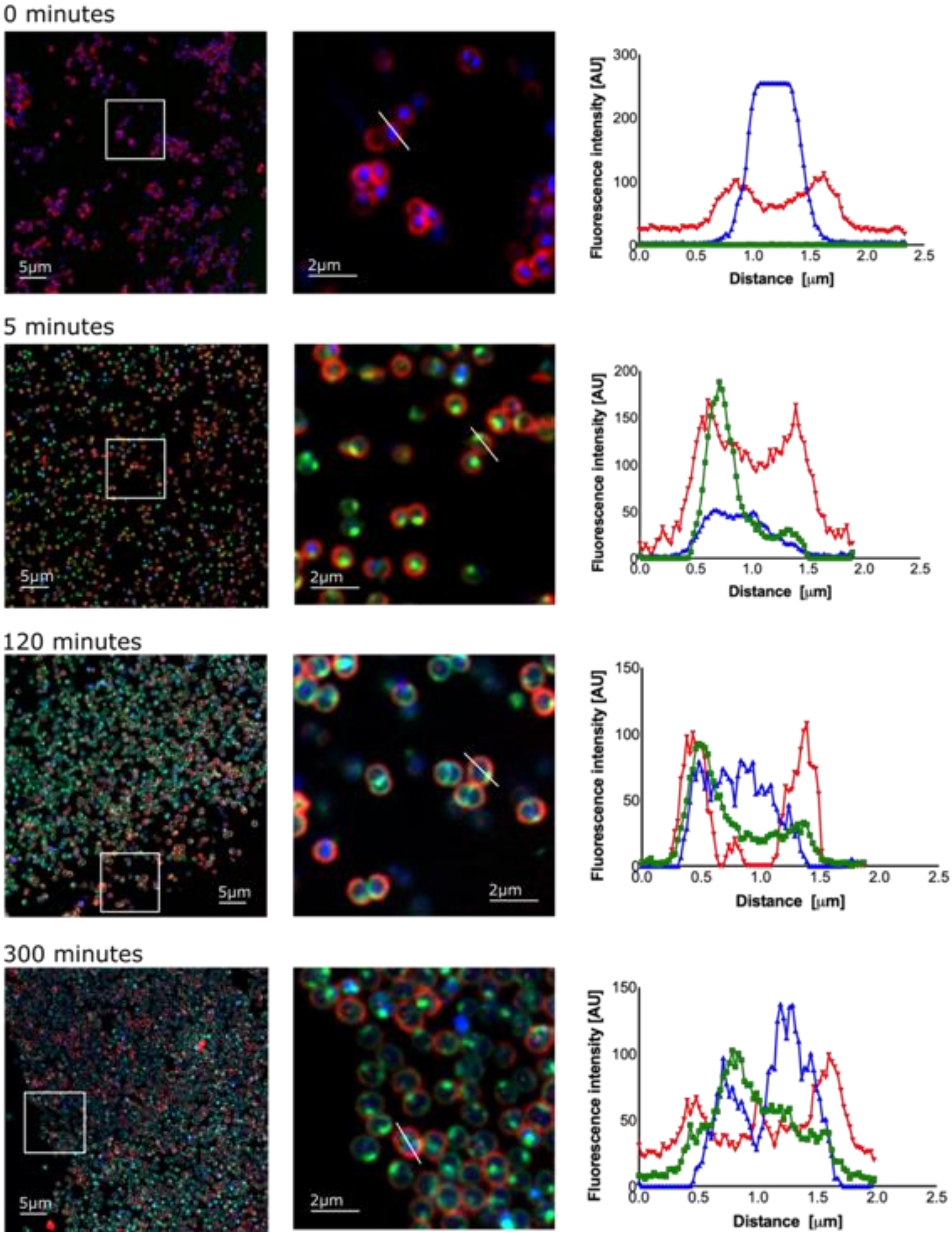

319

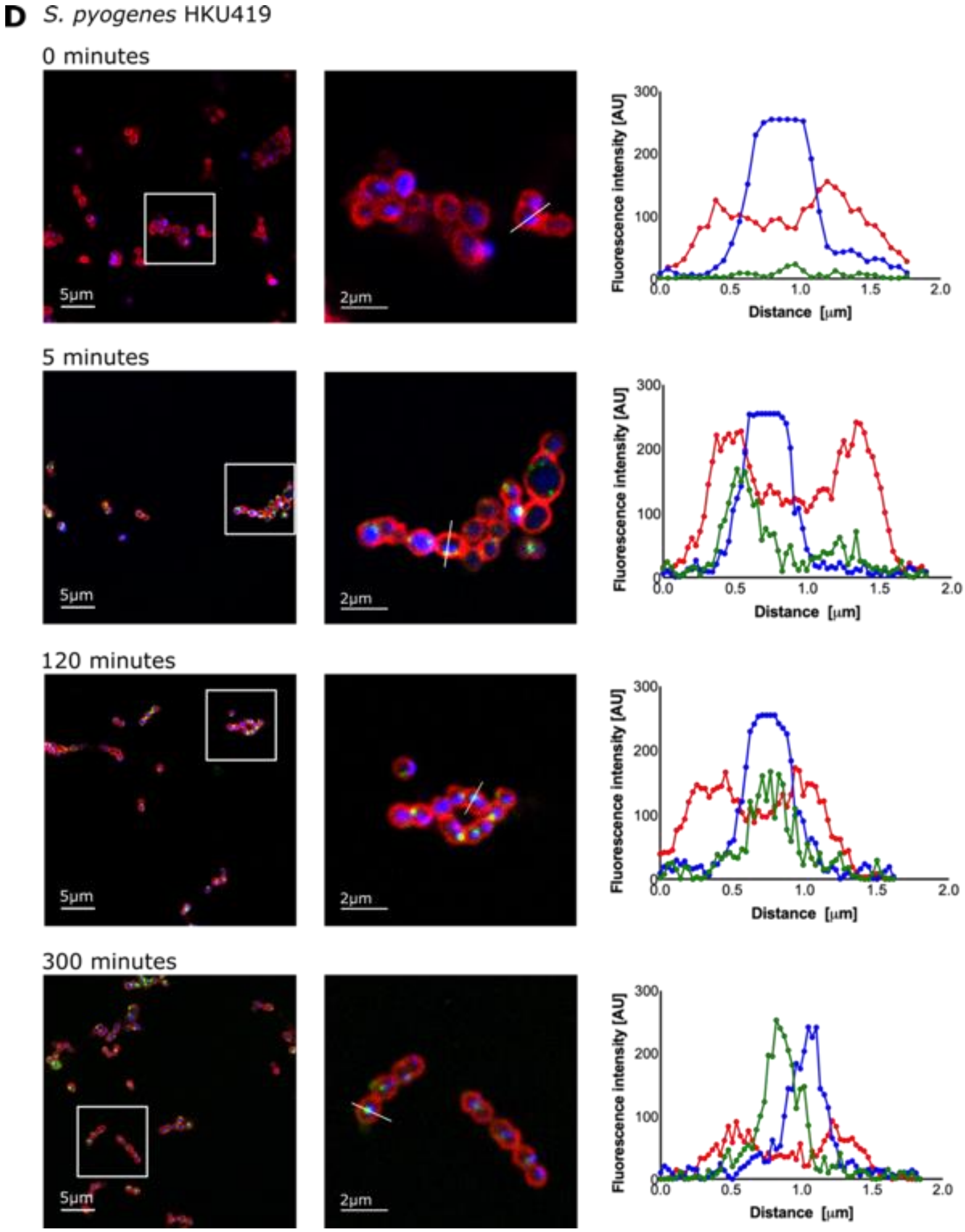

Supplementary Figure 13. Quantification of the interaction of bacterial sepsis strains with cholesterol over time. Indicated bacterial strains were grown for 0 min, 5 min, 120 min or 300 min in RPMI in the presence of 10µM TopFluor-cholesterol (a fluorescent cholesterol analogue). (A-D) Overview (left panel) and close-ups (centre panel) of clinical strains *E. coli*

B36 (A), *K. pneumoniae* KPC2 (B), *S. aureus* BPH2900 (C) and *S. pyogenes* HKU419 (D). Experiments were done at least in three independent biological replicates for time points at 120 min for all strains. For the other time points (i.e., 0 min, 5 min, and 300 min) the experiment as performed with at least one independent biological replicate for *K. pneumoniae* KPC2, *E. coli* B36, and *S. aureus* BPH2900, and two independent biological replicates for *S. pyogenes* HKU419. TopFluor-cholesterol is shown in green (GFP), bacteria in red (alexa555) and nuclei in blue (DAPI). Right panel shows the histogram of the fluorescence intensity of one representative bacterium with the cross-section marked in the close-up image. Colours in the histogram are adjusted to microscope pictures with bacteria in red, nuclei in blue and TopFluor-cholesterol in green.

Supplementary Table 1. Primers used in this study.

**GAS 5448**	
***arcC* deletion replacement**	
**Primers**	**Sequence 5'→ 3'**
CKF	GGGAATTCCAGCTGTTGTCACTCAAGT
CKR	GGGGATCCACACCAGTCAGGG
CKF-Up	ATGACGAAACAAAAAATCGTAGTCGCA
CKR-Down	TTACCCTGCGATAATTTGTGTTCCAG
ErmF	ATGAACAAAAATATAAAATATTCTCAAAAC
ErmR	TTATTTCCTCCCGTTAAATAATAGATA
***arcC* complementation**	
**Primers**	**Sequence 5'→ 3'**
arcC_F-Up	GTCGTCAGACTGATGGGCCCCTAAAGATGCTCCCGATG
arcC_R-Down	CATAACCTGAAGGAAGATCTCATATTAACAACAAGGCCTTC
arcC_F	AGGAGTAATTATGACGAAACAAAAAATCG
arcC_R	ATCCTCTTGATTACCCTGCGATAATTTG
***E. coli* B36 and *E. coli* EC958**	
**Primers**	**Sequence 5'→ 3'**
3518-2394wcaF-Fwsc	AGCGAACCAGATAACGGTA
3519-2394wcaF-Fwup	ACTCGGGCGATATTTTTCAT
3520-2394wcaF-Rvup	GGAATAGGAACTAAGGAGGACGGCACCGAGAATCCACTTA
3521-2394wcaF-Fwdn	CCTACACAATCGCTCAAGACTAAATTCAAAAAATACAGAG
3522-2394wcaF-Rvdn	AACGGCGTGGTCTCTTTCTG
3523-2394wcaF-Rvsc	CCGTAGGATTCGCGGTAGTT
11474_carB_Fwup	GTCGCCTGACCATCGTTC
11475_carB_Rvup	GGAATAGGAACTAAGGAGGATGGCATGGCTCTTTTACTCC
11476_carB_Fwdn	CCTACACAATCGCTCAAGACGCGCAGATCAAATAATAGCG
11477_carB_Rvdn	CTGCTCGTAAGGCATCAGACT
11478_carB_Fwsc	CAAGGGAGCTGGACACTG
11479_carB_Rvsc	CAACTTCGTTACTTACGGCC
3746-Cm.3a	TCCTCCTTAGTTCCTATTCC
3747-Cm.4a	GTCTTGAGCGATTGTGTAGG
