# Supplementary material for: Integrative omics identifies conserved and pathogen-specific responses of sepsis-causing bacteria
Source: Nat Commun. 2023 Mar 18;14:1530. doi: 10.1038/s41467-023-37200-w (PMC10024524; doi:10.1038/s41467-023-37200-w)
Supplement: Supplementary file 3 — Description of Additional Supplementary Files [file 41467_2023_37200_MOESM3_ESM.pdf]

File name: Supplementary Data 1
Description: Orthologue clusters conserved across all four E. coli genomes

File name: Supplementary Data 2
Description: Orthologue clusters conserved across all four Klebsiella pneumoniae species complex genomes

File name: Supplementary Data 3
Description: Orthologue clusters conserved across all four S. aureus genomes

File name: Supplementary Data 4
Description: Orthologue clusters conserved across all four S. pyogenes genomes

File name: Supplementary Data 5
Description: Orthologue clusters conserved across all 20 genomes

File name: Supplementary Data 6
Description: Non-redundant list of gene ontology terms associated with 125 conserved orthologue clusters

File name: Supplementary Data 7
Description: Differential expression analysis of transcriptomics data. This data can also be viewed as an interactive webpage here.

File name: Supplementary Data 8
Description: Gene-set enrichment analysis using GO terms and KEGG metabolic pathways. This data can also be viewed as an interactive webpage here.

File name: Supplementary Data 9
Description: Fold-change values for all features detected in proteomics data across all strains.

File name: Supplementary Data 10
Description: Fold-change values for all features detected in metabolomics data across all strains.

File name: Supplementary Data 11
Description: Demographic, assembly and genome characteristic information, and accession numbers for omic datasets for isolates investigated in this study

File name: Supplementary Data 12
Description: Percentage of the proteins accurately quantified for isolates investigated in this study
