# Supplementary material for: Integrative omics identifies conserved and pathogen-specific responses of sepsis-causing bacteria
Source: Nat Commun. 2023 Mar 18;14:1530. doi: 10.1038/s41467-023-37200-w (PMC10024524; doi:10.1038/s41467-023-37200-w)
Supplement: Supplementary file 16 — Reporting Summary [file 41467_2023_37200_MOESM16_ESM.pdf]

nature portfolio

Corresponding author(s): Mark Walker

Last updated by author(s): 2023/02/25

Reporting Summary

Nature Portfolio wishes to improve the reproducibility of the work that we publish. This form provides structure for consistency and transparency in reporting. For further information on Nature Portfolio policies, see our Editorial Policies and the Editorial Policy Checklist.

Statistics

For all statistical analyses, confirm that the following items are present in the figure legend, table legend, main text, or Methods section.

n/a	Confirmed	
☐	☒	The exact sample size (*n*) for each experimental group/condition, given as a discrete number and unit of measurement
☐	☒	A statement on whether measurements were taken from distinct samples or whether the same sample was measured repeatedly
☐	☒	The statistical test(s) used AND whether they are one- or two-sided<br>*Only common tests should be described solely by name; describe more complex techniques in the Methods section.*
☐	☒	A description of all covariates tested
☐	☒	A description of any assumptions or corrections, such as tests of normality and adjustment for multiple comparisons
☐	☒	A full description of the statistical parameters including central tendency (e.g. means) or other basic estimates (e.g. regression coefficient) AND variation (e.g. standard deviation) or associated estimates of uncertainty (e.g. confidence intervals)
☐	☒	For null hypothesis testing, the test statistic (e.g. *F*, *t*, *r*) with confidence intervals, effect sizes, degrees of freedom and *P* value noted<br>*Give P values as exact values whenever suitable.*
☒	☐	For Bayesian analysis, information on the choice of priors and Markov chain Monte Carlo settings
☐	☒	For hierarchical and complex designs, identification of the appropriate level for tests and full reporting of outcomes
☒	☐	Estimates of effect sizes (e.g. Cohen's *d*, Pearson's *r*), indicating how they were calculated

Our web collection on statistics for biologists contains articles on many of the points above.

Software and code

Policy information about availability of computer code

Data collection	Primary DNA sequencing, RNA sequencing, proteomic, and metabolomic data were generated using commercial software relevant for each analytical machine. That is, Illumina, Pacific Biosciences (PacBio), Thermo LC-MS systems with XCalibur v3.1, MaxQuant (v1.5.5.1), and metabolite detection using the Smart Metabolites Database v3 (Shimadzu) and
Data analysis	The following programs were used in this study: Unicycler v0.4.6; Canu v1.8.0; HGAP v3 pipeline, SMRTPortal v2.3.0; Snippy v4.3; mlst v2.19.0 (https://github.com/tseemann/mlst); abriTAMR v0.2.2 (https://github.com/MDU-PHL/abritamr); emmtyper v0.1.0 (https://github.com/MDU-PHL/emmtyper); Kleborate v2.0.1; spaTyper and SeroTypeFinder; ABRIcate (https://github.com/tseemann/abricate); Mashtree 1.1.2; FigTree 1.4.4; pirate v1.0.2; R v3.6.3; Blast2GO; eggNOG database v5.0; PANNZER2 webserver; minimap2 v2.17-r94; featureCounts v2.0.1; R package edgeR; MaxQuant (v1.5.5.1); The Automatic Adjustment of Retention Time (AART) in GCMSsolution software (version 4.42, Shimadzu); Shimadzu LabSolutions GC-MS browser software software v4.42; MassHunter TOF Quantitative Analysis software (version B.07.00, Agilent Technologies); Fiji/ImageJ (https://imagej.nih.gov/ij/) or Imaris Viewer software (https://imaris.oxinst.com/imaris-viewer); Guppy v.6.1.5; clusterProfiler v3.16; Agilent 1200 series HPLC system coupled with a 6545 Q-TOF MS using Agilent MassHunter Workstation LC/MS Data Acquisition for 6200 series TOF/6500 series Q-TOF (version 10.1).

For manuscripts utilizing custom algorithms or software that are central to the research but not yet described in published literature, software must be made available to editors and reviewers. We strongly encourage code deposition in a community repository (e.g. GitHub). See the Nature Portfolio guidelines for submitting code & software for further information.

Data

Policy information about availability of data

All manuscripts must include a data availability statement. This statement should provide the following information, where applicable:

- Accession codes, unique identifiers, or web links for publicly available datasets
- A description of any restrictions on data availability
- For clinical datasets or third party data, please ensure that the statement adheres to our policy

Details of accession numbers for all strains across genomics, transcriptomics, proteomics, and metabolomics can be found in Supplementary Table 7.

Escherichia coli (PRJEB29930 [https://www.ncbi.nlm.nih.gov/bioproject/PRJEB29930]; GSE152966 [https://www.ncbi.nlm.nih.gov/geo/query/acc.cgi?acc=GSE152966]; GSE152967 [https://www.ncbi.nlm.nih.gov/geo/query/acc.cgi?acc=GSE152967]; GSE152968 https://www.ncbi.nlm.nih.gov/geo/query/acc.cgi?acc=GSE152968]; GSE152969 [https://www.ncbi.nlm.nih.gov/geo/query/acc.cgi?acc=GSE152969]; PXD016840 [https://www.ebi.ac.uk/pride/archive/projects/PXD016840]; PXD020545 [https://www.ebi.ac.uk/pride/archive/projects/PXD020545]; MTBLS2015 [https://www.ebi.ac.uk/metabolights/MTBLS2015/samples]), Klebsiella pneumoniae species complex (PRJEB29928 [https://www.ncbi.nlm.nih.gov/bioproject/PRJEB29928]; GSE152839 [https://www.ncbi.nlm.nih.gov/geo/query/acc.cgi?acc=GSE152839]; GSE1528340 [https://www.ncbi.nlm.nih.gov/geo/query/acc.cgi?acc=GSE152840]; GSE152843 [https://www.ncbi.nlm.nih.gov/geo/query/acc.cgi?acc=GSE152843]; GSE152844 [https://www.ncbi.nlm.nih.gov/geo/query/acc.cgi?acc=GSE152844]; GSE152847 [https://www.ncbi.nlm.nih.gov/geo/query/acc.cgi?acc=GSE152847]; GSE152964 [https://www.ncbi.nlm.nih.gov/geo/query/acc.cgi?acc=GSE152964]; PXD016672 [https://www.ebi.ac.uk/pride/archive/projects/PXD016672]; PXD020839 [https://www.ebi.ac.uk/pride/archive/projects/PXD020839]; MTBLS2322 [https://www.ebi.ac.uk/metabolights/MTBLS2322/samples]), Staphylococcus aureus (PRJEB29881 [https://www.ncbi.nlm.nih.gov/bioproject/?term=PRJEB29881]; GSE152833[https://www.ncbi.nlm.nih.gov/geo/query/acc.cgi?acc=GSE152833]; GSE152834 [https://www.ncbi.nlm.nih.gov/geo/query/acc.cgi?acc=GSE152834]; GSE152835 [https://www.ncbi.nlm.nih.gov/geo/query/acc.cgi?acc=GSE152835]; GSE152837 [https://www.ncbi.nlm.nih.gov/geo/query/acc.cgi?acc=GSE152837]; GSE152838 [https://www.ncbi.nlm.nih.gov/geo/query/acc.cgi?acc=GSE152838]; PXD016504 [https://www.ebi.ac.uk/pride/archive/projects/PXD016504]; PXD020791 [https://www.ebi.ac.uk/pride/archive/projects/PXD020791]; MTBLS1898 [https://www.ebi.ac.uk/metabolights/MTBLS1898/samples]), and Streptococcus pyogenes (PRJEB29800 [https://www.ncbi.nlm.nih.gov/bioproject/?term=PRJEB29800]; GSE152821 [https://www.ncbi.nlm.nih.gov/geo/query/acc.cgi?acc=GSE152821]; GSE152822 [https://www.ncbi.nlm.nih.gov/geo/query/acc.cgi?acc=GSE152822]; GSE152823 [https://www.ncbi.nlm.nih.gov/geo/query/acc.cgi?acc=GSE152823]; GSE152824 [https://www.ncbi.nlm.nih.gov/geo/query/acc.cgi?acc=GSE152824], GSE152826 [https://www.ncbi.nlm.nih.gov/geo/query/acc.cgi?acc=GSE152826]; PXD016913 [https://www.ebi.ac.uk/pride/archive/projects/PXD016913]; PXD020863 [https://www.ebi.ac.uk/pride/archive/projects/PXD020863]; MTBLS2324 [https://www.ebi.ac.uk/metabolights/MTBLS2324/samples]).

Field-specific reporting

Please select the one below that is the best fit for your research. If you are not sure, read the appropriate sections before making your selection.

☒ Life sciences ☐ Behavioural & social sciences ☐ Ecological, evolutionary & environmental sciences

For a reference copy of the document with all sections, see nature.com/documents/nr-reporting-summary-flat.pdf

Life sciences study design

All studies must disclose on these points even when the disclosure is negative.

Sample size	The strains selected for each species was in an effort to capture the phylogenetic diversity. The number of biological replicates was based on standard procedures for each omic platform. The number of replicates were validated with empirical data collected through extensive pilot assays in this current study.
Data exclusions	One biological replicate (among the 6 replicates per condition) was discarded as an outlier from RNAseq experiments for Escherichia coli and Stahylococcus aureus. The replicate samples discarded were E. coli B36 RPMI #5, E. coli MS14384 RPMI #1, E. coli MS14386 sera #1, E. coli MS14387 RPMI #1, S. aureus BPH2760 sera #4, and S. aureus BPH2947 sera #4. These samples were excluded after analysis of quality control metrics including total mapped reads, count per million distribution pre- and post-normalisation, multi-dimensional scaling plots, hierarchal clustering of the count correlation matrix and Cook's distance distribution of counts.
Replication	Six biological replicates of each bacterial strain were assayed in both conditions (i.e., growth in RPMI and exposure to human sera). Standard quality control metrics (including multi-dimensional scaling) were assessed for each omic platform to determine reproducibility of each strain.
Randomization	Primary derived samples were randomised for mass spectrometry analysis. Other co-variates - such as blood type for human sera samples - were also controlled by randomisation of the pooling from different donors. The remaining assays (e.g., DNA and RNA sequencing) did not require randomization in sample collection.
Blinding	Researchers were not blinded by way of knowing which samples were derived from strains grown in RPMI media or exposed to human sera. However, researchers from the respective omic platforms were blinded by way of not knowing which features were enriched or depleted. For example, researchers working on the metabolomics dataset did not initially know which transcripts or proteins were enriched when the strains were exposed to human sera.

Reporting for specific materials, systems and methods

We require information from authors about some types of materials, experimental systems and methods used in many studies. Here, indicate whether each material, system or method listed is relevant to your study. If you are not sure if a list item applies to your research, read the appropriate section before selecting a response.

Materials & experimental systems

n/a	Involved in the study
☐	☒ Antibodies
☒	☐ Eukaryotic cell lines
☒	☐ Palaeontology and archaeology
☒	☐ Animals and other organisms
☒	☐ Human research participants
☒	☐ Clinical data
☒	☐ Dual use research of concern

Methods

n/a	Involved in the study
☒	☐ ChIP-seq
☒	☐ Flow cytometry
☒	☐ MRI-based neuroimaging

Antibodies

Antibodies used

E. coli: O25 monospecific O rabbit antiserum (85022, SSI Diagnostica); K. pneumoniae: monoclonal antibody raised in BALB/c mice against the sarkosyl-insoluble outer membrane fraction of a non-mucoid strain of Klebsiella pneumoniae B5055. Fab fragments of this antibody were produced by digestion on immobilised papain (Pierce) and purification on a protein A Sepharose column (Pierce)59; S. aureus: polyclonal antibody serum were raised in rabbits against whole fixed cells of Staphylococcus aureus USA300 strains, BPH2919 and BPH3672 (Custom antibody produced by WEHI antibody technology platform, https://www.wehi.edu.au/research/research-technologies/antibody-technologies); S. pyogenes: rabbit Streptococcus Group A polyclonal (Cat. No. PAB13831, Abnova). Goat anti-mouse Alexa Fluor555 (Cat. No. A28180, Thermo Fisher Scientific)

Validation

E. coli Ab: validation through commercial supplier SSI Diagnostica. SSI Diagnostica's development, production and sales of in vitro diagnostics are quality assured and certified in accordance with ISO 13485. Certificate of analysis can be downloaded from our website: ssidiagnostica.com; KpSC: testing by Jenney, A. W. J. The use of monoclonal antibodies to investigate vaccine antigens of klebsiella pneumoniae. (2006).; S. aureus: WEHI antibody technology platform, https://www.wehi.edu.au/research/research-technologies/antibody-technologies; S. pyogenes: validation through commercial supplier Cat. No. PAB13831, Abnova.
